# Specific ion effects on ion transport in charged polymer membranes

**DOI:** 10.1126/sciadv.adx1214

**Published:** 2026-03-11

**Authors:** David Kitto, Gregory Reimonn, Riley Vickers, Carolina Espinoza, Aeva G. Silverman, Bryan R. Goldsmith, Jovan Kamcev

**Affiliations:** ^1^ Department of Chemical Engineering, University of Michigan, Ann Arbor, MI 48105, USA.; ^2^ Macromolecular Science and Engineering, University of Michigan, Ann Arbor, MI 48105, USA.

## Abstract

Selective separation of like-charged ions is a central challenge in applications such as critical mineral recovery. Electrochemical membrane–based separations offer promising pathways to address this need, but a limited fundamental understanding of ion transport in charged polymer membranes hampers the development of highly selective materials. This study elucidates the role of specific ion effects (SIEs) in ion transport through charged polymer membranes by integrating experimental measurements of ion mobility and in situ ion/ion and ion/water interactions in model charged polymer membranes with results from molecular dynamics simulations on analogous systems. We demonstrate that solvent-mediated ion interactions drive pronounced SIEs within the studied membranes, with the ion softness, i.e., the malleability of ion hydration shells, from hard/soft acid/base (HSAB) theory emerging as a key predictor of transport properties. HSAB theory also explains the observed mechanism of solvent-mediated ion interactions. Our findings offer a mechanistic framework for designing membranes with tailored ion selectivity, potentially enabling efficient separations of chemically similar ions.

## Teaser

Specific ion effects in charged polymers are governed by ion softness, i.e., the malleability of the ion hydration shell.

## INTRODUCTION

The ability to selectively separate ions of similar charge is becoming increasingly valuable in various applications, including water treatment and critical mineral recovery (*1*–*13*). Membrane-based technologies offer an energy-efficient solution for these like-charge ion separations, but their effectiveness depends on the ions of interest. Typically, separations involving ions of different valences are more readily achieved than those involving ions of the same valence. For example, monovalent-selective ion-exchange membranes (IEMs) leverage electrostatic forces to exclude divalent ions more effectively than monovalent ions, thereby achieving like-charge separation (*2*, *14*, *15*). Furthermore, ions of higher valence exhibit greater hydrated sizes than ions of lower valence, enabling nanofiltration membranes to achieve charge and size-based separation of like-charged ions (*1*, *3*). Membranes containing specific complex-forming moieties can leverage the larger electron shells of multivalent ions to form coordination interactions, accomplishing targeted adsorptive removal of higher-valent ions (*1*, *3*, *14*). In contrast, highly selective separations of like-charged monovalent ions remain difficult for polymeric membranes.

Separation of like-charged ions could potentially be achieved by leveraging specific ion effects (SIEs), which refer to differences in ion behavior that are not captured in their simplified treatment as point charges (*16*–*19*). Notably, polyelectrolytes in solution exhibit SIEs in numerous contexts, with these trends famously described in the Hofmeister series (*20*). Hofmeister and others have proposed hierarchical ordering of ions to explain how various properties of polyelectrolyte/salt mixtures depend on the identity of the ions comprising the electrolyte (*21*, *22*). Because IEMs are essentially networks of polyelectrolytes, the extensive understanding of polyelectrolyte SIEs suggests that IEMs may hold potential for like-charge monovalent ion separations enabled by SIEs. However, SIEs in IEMs are not well understood on a fundamental level, hindering the design of IEMs for specific like-charge separations.

Although SIEs in polyelectrolyte solutions are better understood than in IEMs, SIEs in both systems remain unpredictable. Notably, the ordering of the Hofmeister and Hofmeister-inspired series varies with the electrolyte property being studied (*16*). Moreover, the qualitative order of the series does not yield quantitative predictions of the relative strength of SIEs. This uncertainty persists because the ion property that determines any chosen order of a Hofmeister series is still unclear (*16*–*18*). Most explanations, including the one originally proposed by Hofmeister, focus on the extent of ion interaction with surrounding water molecules (*16*, *20*). These explanations have collectively developed into Collin’s Law of Matching Water Affinities (LMWA) (*16*, *17*). A popular iteration of the LMWA equates the Hofmeister series to the Lyotropic series, which is unambiguously ordered by the ion enthalpies of hydration (*16*, *17*, *19*). However, other metrics of water affinity are also evoked in the LMWA (*16*–*19*, *23*, *24*), and other more ion-centric properties (*16*–*19*, *23*–*25*) are considered possible sources of SIEs. To exploit SIEs for like-charge membrane-based separations, it is crucial to develop a detailed understanding of the source of SIEs in ion transport across IEMs.

Several notable investigations have explored the topic of SIEs in charged polymer membranes and resins (*26*–*30*). In pioneering works by Boyd and Soldano (*31*–*34*), transition state theory (TST) was used to examine the activation energies and entropies associated with the diffusion of various ions in ion-exchange resins. Their findings revealed distinct trends for cations and anions, particularly with respect to ion size and valence. They speculated that dehydration and ion association drive the enthalpically dominated trends observed for the cations and the entropically dominated trends for the anions, and these general ideas have survived through the years as conventional knowledge. Recently, interest in applying TST to ion transport in IEMs has resurged (*35*–*39*). Although not all recent studies have focused on SIEs, some have leveraged this framework to better understand ion-specific transport properties (*38*, *39*). Badessa and Shaposhnik investigated the roles of hydrogen bonding and fixed charge/mobile ion interactions for cations, accurately predicting experimental activation energies of conduction for monovalent, divalent, and trivalent cations (*38*). They found that hydrogen bonding played a dominant role in the monovalent cation trends, attributing the SIEs to the enthalpy of hydration. Epsztein *et al.* studied monovalent cations and anions in a pair of commercial IEMs, identifying a correlation between the free energy of hydration and the measured activation energies of permeation (*39*). They proposed that ion dehydration underpinned this trend but noted deviations for larger, nonspherical ions due to effects of ion orientation. Although these studies propose plausible mechanisms for ion-specific transport properties among like-charge ions, they lack a quantitative comparison between ion properties and transport behavior in the membrane, leaving ambiguity in what the true cause of their observed trend is.

In this study, we performed a comprehensive experimental and modeling investigation aimed to advance the study of SIEs in IEMs, partly inspired by the success of this approach in studying other membrane systems (*40*). We quantified the transport of eight cations and seven anions in homogeneous IEMs with well-defined chemical structures. Using dilute aqueous solution conditions as a controlled baseline, we probed how the membrane matrix influences the transition state energetics of ion transport, revealing strong SIEs. All-atom molecular dynamics (MD) simulations of these model membrane systems support experimental observations and provide mechanistic insights into resolving the phenomena underpinning the observed SIEs. Our findings reveal that Ahrland’s measure of ionic softness, which quantifies the malleability of water solvating an ion (*18*, *41*), strongly correlates with the energy required for ion transport within these IEMs. Furthermore, the mechanism of ion interaction proposed by Ahrland is consistent with the phenomena detected within these membranes. In short, the limited availability of water in the membranes strengthens interactions between ions, water, and the polymer matrix, leading to collaborative ion hydration and substantial changes in ion transport. Building on this understanding, we propose specific membrane designs capable of tuning the energy profile for transport of nearly identical ions, potentially enabling targeted like-charge monovalent ion separations via IEMs.

## RESULTS

**Fig. 1.**
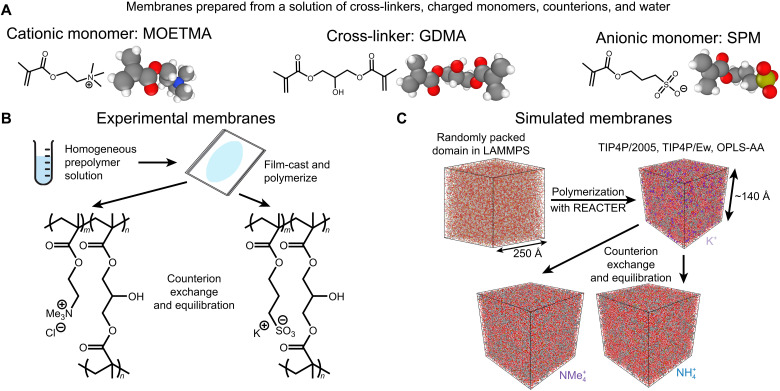
Membranes under study. (**A**) Monomers used to prepare the membranes in this study. AEMs were prepared as copolymers of MOETMA and GDMA, whereas CEMs were prepared as copolymers of SPM and GDMA. (**B**) Physical membranes were prepared via thermally initiated free radical polymerization of the aqueous comonomer system, followed by counterion exchange and equilibration with DI water. (**C**) Simulated membranes were prepared by randomly placing monomer molecules into an oversized domain, which was then equilibrated and allowed to collapse. A fraction of methacrylate functionalities was then activated and allowed to polymerize until the extent of network incorporation reached sufficient levels. After polymerization, the counterions were replaced with new ions, and water molecules were inserted or removed to match the experimentally determined water content. These domains were then equilibrated before beginning equilibrium sampling.

We synthesized cross-linked polymeric anion-exchange membranes (AEMs) and cation-exchange membranes (CEMs) with similar chemical structures and identical comonomer ratios, producing IEMs that differ only in the chemical identity of the fixed charge groups (Fig. 1 and table S1). The polymerization conditions were also tuned to yield AEMs and CEMs exhibiting nearly identical water and charge contents in their Cl^−^ and Na^+^ forms, respectively (figs. S2 and S3). Aligning the membrane properties with these common ions, considered relatively average in the Hofmeister and LMWA series (*22*), ensures a fair comparison of the anion and cation data. By considering both cation and anion data in a single test matrix, we aim to describe universal SIEs that are present in both AEMs and CEMs. After membrane synthesis and counterion exchange, we measured the charge and water contents of the IEMs in each of 15 counterion forms to investigate any counterion-dependent swelling differences and quantify the extent of conversion. The counterion conversions were successful (table S2), with gravimetric charge contents measured to be within 18% of the theoretical ion-exchange capacities (IECs). The water volume fractions, ϕ_*w*_, varied minimally across most membrane counterion forms and are recorded in fig. S2 and table S3.

We also implemented atomistic MD simulations (Fig. 1C) to establish a molecular-level understanding of the interactions between ions, polymer, and water for these systems. Starting with simulated prepolymer solutions analogous to those used experimentally (fig. S4 and table S4), we prepared an AEM and CEM in silico using the large-scale atomic/molecular massively parallel simulator (LAMMPS) (*42*). The methacrylate functionalities were activated and propagated into a polymer backbone and grown into a network using REACTER (*43*, *44*) (figs. S5 to S7 and table S5). Following polymerization, we performed ion exchange and equilibration steps to afford simulated IEMs in 11 monovalent counterion forms (figs. S8 and S9 and tables S6 and S7). These MD simulations used the TIP4P/2005 (*45*) water model and TIP4P/Ew (*46*, *47*) parameterization for the monatomic mobile ions, whereas the optimized potential for liquid simulation (OPLS) force field (*48*, *49*) was used to parameterize all other multiatomic molecules. Equilibrium interactions were assessed within IEM domains with side lengths of ~140 Å, containing over 2500 mobile counterions. By averaging the positions of species over the final 5 ns after equilibration was achieved, we developed a detailed picture of the interactions occurring within the simulated membranes. The simulated results were then extensively compared with the experimental results to ensure the simulations adequately captured the behavior of the real system. The IECs of simulated IEMs are only slightly higher (<8%) than the experimentally determined IECs (table S5) of the physical membranes, indicating that the total incorporation of monomer into the membranes was comparable between these two systems. Furthermore, the simulated IEMs achieved similar hydrated densities to the experimental IEMs (tables S6 and S7), indicating that water interactions and molecular packing were reasonably well represented by the MD parameterization.

### The transition state of ion motion

We used the TST framework developed by Eyring and Polanyi (*50*, *51*) to quantify the energetics of transport in the experimental IEMs and understand SIEs. To do so, we measured the ionic conductivities of deionized (DI) water–equilibrated IEMs in each counterion form as a function of temperature from 10° to 60°C (figs. S10 and S11 and tables S8 and S9). These measurements were performed using electrochemical impedance spectroscopy (EIS) via the direct contact method (*52*) (section S1.7), allowing us to isolate and extract the intrinsic membrane ionic conductivity. For each ion, the membrane water content at 10° and 60°C was similar to the value measured at room temperature (fig. S2), so any temperature dependence of the ionic conductivity should arise from differences in the ion mobility rather than the ion concentration. The membrane ionic conductivities and other derived values all increase monotonically with temperature, as shown in the respective Arrhenius plots (figs. S11 to S14). Comparing the effective ion conductances in the membrane (fig. S15) to those in aqueous solution (fig. S16) reveals a notable difference between cations and anions. Although the relative order of cation conductances in the CEM are similar to those observed in aqueous solutions, the relative order of anion conductances in the AEM is essentially reversed from that in aqueous solutions. Specifically, cation conductances in the CEM and both cation and anion conductances in aqueous solution increase with decreasing size of the hydrated ion, whereas the opposite trend is observed for anions in the AEM.

These conductance datasets allowed for the calculation of the enthalpic and entropic energetics of ion transport. Within the TST framework, ions execute translational steps by overcoming a free energy barrier associated with entering the transition state of motion (∆*G*^‡^) (*50*, *51*), which is then broken down into enthalpic (∆*H*^‡^) and entropic (∆*S*^‡^) components

$$∆G^{‡}=∆H^{‡}-T∆S^{‡}=\left(\mathrm{RT}+E_{a}\right)-T∆S^{‡}$$ (1)

Here, *R* is the ideal gas constant and *T* is the absolute temperature. The enthalpy can be further reduced to yield the temperature-independent enthalpic energy barrier, i.e., the activation energy (*E*_*a*_) (*50*, *51*). To connect these energetics to actual rates of transport, TST uses a Boltzmann distribution, alongside the frequency and distance of ion hops (*50*, *51*)

$$u_{i}=\frac{\lambda_{i}}{|z_{i}|\mathrm{eF}}=\frac{\delta^{2}}{6h}\text{exp}\left(\frac{∆S^{‡}}{R}+1\right)\text{exp}\left(-\frac{E_{a}}{\mathrm{RT}}\right)$$ (2)

Here, *u*_*i*_ is the absolute mobility of ion *i*, λ_*i*_ is its equivalent conductance, and *z*_*i*_ is its valence. *e* is the protonic charge, *F* is Faraday’s constant, *h* is Planck’s constant, and δ is the distance of a single ion jump [assumed to be 2.8 Å (*51*, *53*)]. The factor of 6 arises from the Einstein-Smoluchowski equation (see section S2.3.1) (*35*, *53*). The activation energy in Eq. 2 is notably the activation energy of motion, rather than the activation energy of diffusion (this distinction is not often discussed, so we develop this concept extensively in section S2.3).

**Fig. 2.**
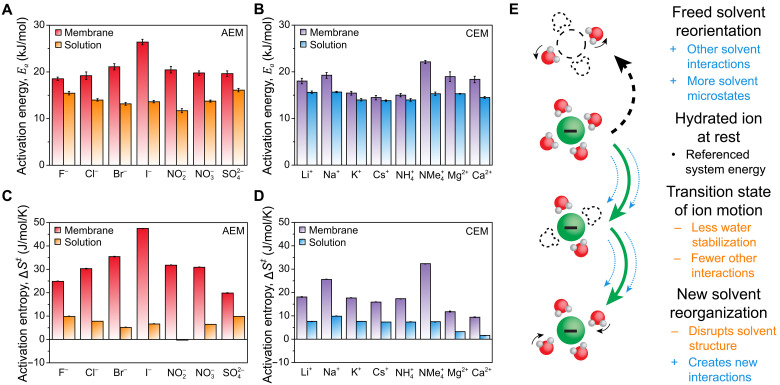
Transition state of ion motion. Activation energies of ion motion in IEMs ($E_{a}^{m}$) and in aqueous solution at infinite dilution ($E_{a}^{s}$) for (**A**) anions and (**B**) cations. Activation entropies of ion motion in IEMs [${\left(∆S^{‡}\right)}^{m}$] and in aqueous solution at infinite dilution [${\left(∆S^{‡}\right)}^{s}$] for (**C**) anions and (**D**) cations. Uncertainties for the membrane results represent the SEM for the linear fit performed on tortuosity-corrected equivalent ionic conductance data. Uncertainties for the solution results represent the SEM for the linear fit performed on sampled data points calculated using literature equivalent ionic conductance data (table S10). Tabulated values for the activation energies and entropies are given in table S11. A sensitivity analysis of the linear fitting parameters used to calculate activation energies and entropies is shown in table S12. The solution data for NO_2_^−^ were evaluated over a narrower temperature range than the other ions, as discussed in section S2.2. (**E**) Phenomena that influence the enthalpy and entropy difference between the resting state and the activated state for an ion in motion. Energy penalties are shown in orange (−) and energy benefits are shown in blue (+).

Equations 1 and 2 allow us to interpret our conductance-temperature data at the molecular level: The activation energy, *E*_*a*_, represents the added energy required for an ion to enter the activated state of motion, whereas the activation entropy, ∆*S*^‡^, quantifies the change in local system entropy associated with this transition. To ensure meaningful molecular transport rate energetics, transport data must be corrected for nonlocal effects before applying Eq. 2. Specifically, the ion transport data were corrected for geometric obstruction/tortuosity effects (which do not affect energetics) using the equations in section S2.3.4. Neglecting these corrections can yield quantitatively different activation entropies, leading to notably altered interpretations of transport phenomena. We refer the interested reader to sections S2.3.3 and S2.3.4 for a detailed discussion of rate process analyses. Following these procedures, the results in figs. S15 and S16 were analyzed to isolate the activation energies and entropies of ion motion in the membranes and aqueous solution (Fig. 2).

Both cations and anions encounter a higher enthalpic energy barrier to transport within the membrane than in a dilute aqueous solution, as demonstrated in Fig. 2 (A and B). Although $E_{a}^{m}>E_{a}^{s}$ for all ions, the difference is generally more pronounced for the anions than for the cations. Because $E_{a}^{m}>E_{a}^{s}$, two possible enthalpic interactions are implicated (Fig. 2E): (i) Resting ions in the membrane experience stronger stabilizing enthalpic interactions compared to resting ions in aqueous solution; or (ii) activated ions in the membrane experience weaker stabilizing interactions compared to activated ions in aqueous solution. The electric field of the membrane fixed charge groups presents a likely explanation for the first mechanism (*34*, *36*, *38*, *54*), but ion dehydration is a plausible explanation for the second mechanism (*39*, *54*). Either mechanism (or a combination of the two) could be feasible, and both have been evoked to explain ion transport results in past studies (*34*, *36*, *38*, *39*, *54*, *55*).

Explaining the results for ∆*S*^‡^ in Fig. 2 (C and D) requires a solvent-focused analysis involving the states of water that surround an ion (*33*, *36*). In aqueous systems, because of the strong hydrogen-bonding network, the rearrangement of microstates caused by (de)orienting the quadrupole of nearby waters is considered to be the dominant entropic change associated with solute transport (Fig. 2E) (*51*). For ionic species, some waters are strongly oriented by the electric field, such that these waters transport with the ion over diffusion-relevant timescales (*53*). Other weakly oriented waters may coordinate a resting ion but do not transport along with the ion (*53*). When an ion enters its activated state of motion, weakly oriented waters cease to interact with the ion and are free to interact more strongly with their surroundings (*34*, *53*). Negative values of ∆*S*^‡^ (observed for NO_2_^−^ in dilute aqueous solution) suggest that more microstates are available for the local ion and water system when the ion is present than when it is absent. This observation could be related to the strong dipole uniquely present in NO_2_^−^, which has been shown to enhance the hydrogen bonding of water in its vicinity (*56*). Conversely, positive values of ∆*S*^‡^ (observed for all other ions in dilute aqueous solution) suggest that more microstates are available for the local ion/water system when the water molecules are free to form a hydrogen bond network without incorporating the ion.

Because ${\left(∆S^{‡}\right)}^{s}$ is dominated by the number of water interactions available after an ion enters the activated state, it is no surprise that the hydrophilic polymer, which occupies ~45% of the membrane volume, produces values of ${\left(∆S^{‡}\right)}^{m}>{\left(∆S^{‡}\right)}^{s}$ for each ion under study. Large positive values of ${\left(∆S^{‡}\right)}^{m}$ indicate that, once weakly oriented water molecules are freed from the electric field of an ion, they are stabilized by interactions with other waters and polymer functional groups in the membrane. These positive values of ${\left(∆S^{‡}\right)}^{m}$ contrast most reported activation entropies for solute diffusion in membranes (*54*). However, the negative ${\left(∆S^{‡}\right)}^{m}$ values reported in other studies are likely due to neglecting the obstruction/tortuosity corrections, which are crucial for interpreting activation entropies at the molecular level (section 2.3.4). On the basis of this discussion, positive activation entropies are more realistic for hydrophilic polymer membranes decorated with myriad functional groups capable of hydrogen bonding with water.

### SIEs in the transition state of ion motion

**Fig. 3.**
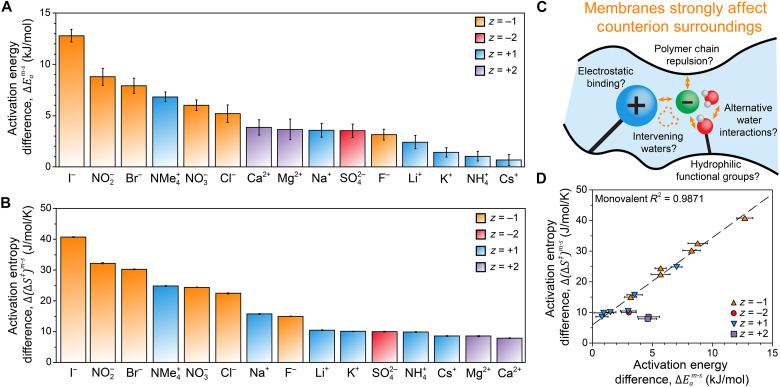
SIEs on the transition state of ion motion in polymer membranes. (**A**) Activation energies and (**B**) activation entropies of ion motion in the IEMs, referenced against corresponding values in dilute aqueous solution [$∆E_{a}^{m-s}$ and $∆{\left(∆S^{‡}\right)}^{m-s}$, respectively]. Data points are ordered independently from largest to smallest in each plot. Uncertainties were calculated from the standard errors of the activation energies and activation entropies using standard error propagation methods. (**C**) Schematic illustrating counterion interactions in charged polymer membranes. (**D**) Linear free energy relationship for membrane effects on the transition state of ion motion. The dashed line represents a linear fit to the monovalent ion data (*z*_*i*_ = −1, orange triangles, and *z*_*i*_ = +1, blue triangles). Divalent ion data (*z*_*i*_ = −2, red circles, and *z*_*i*_ = +2, purple squares) are shown but not included in the linear regression.

Although each ion under study exhibited both $E_{a}^{m}>E_{a}^{s}$ and ${\left(∆S^{‡}\right)}^{m}>{\left(∆S^{‡}\right)}^{s}$, the magnitudes of $E_{a}^{m}$, ${\left(∆S^{‡}\right)}^{m}$, $E_{a}^{s}$, and ${\left(∆S^{‡}\right)}^{s}$ depend on the mobile ion. To elucidate the nature of these SIEs, it is instructive to reference ion transport properties in the membrane against those in aqueous solution (*34*, *36*). This transformation isolates the enthalpic and entropic interactions each ion experiences due to the surrounding membrane environment. Accordingly, we focus the remainder of this discussion on the additional energy required to reach the activated state for an ion in the membrane relative to an ion in an aqueous solution, $∆E_{a}^{m-s}=E_{a}^{m}-E_{a}^{s}$, and the additional entropy derived from entering the activated state for an ion in the membrane relative to an ion in an aqueous solution, $∆{\left(∆S^{‡}\right)}^{m-s}={\left(∆S^{‡}\right)}^{m}-{\left(∆S^{‡}\right)}^{s}$. These transformations are nonlinear (fig. S17) and are themselves ion specific. The solution-referenced TST parameters are presented in Fig. 3.

Comparing the activation energy differences for anions and cations, large hydrated ions (F^−^ and Li^+^) exhibit similar $∆E_{a}^{m-s}$. However, $∆E_{a}^{m-s}$ increases down the halide series toward I^−^, whereas $∆E_{a}^{m-s}$ decreases down the alkali series toward Cs^+^. The multiatomic and multivalent ions do not stand out except for NMe_4_^+^, which experiences the highest $∆E_{a}^{m-s}$ among the cations. For $∆{\left(∆S^{‡}\right)}^{m-s}$, the ordering of cations and anions remains essentially the same as $∆E_{a}^{m-s}$, except all divalent ions are shifted lower in the ranked series of $∆{\left(∆S^{‡}\right)}^{m-s}$. The notable similarity between the trends in $∆E_{a}^{m-s}$ and $∆{\left(∆S^{‡}\right)}^{m-s}$ for the monovalent ions suggests that the ion-specific interactions causing activation energy and entropy differences may be due to the same phenomena. However, the hydrophilic charged polymer surrounding ions in the IEM can alter the enthalpic and entropic components of ion transport via different types of interactions (Fig. 3C), so it is also possible that distinct phenomena contribute to these two trends.

Correlations such as the qualitatively similar trends in Fig. 3 (A and B) can be extended via quantitative analysis. When discussing TST, this observation is referred to as the entropy-enthalpy compensation effect (*54*, *57*), but for more generalized analyses of activated rate processes, it is called the linear free energy relationship (*58*) or simply the compensation effect (*59*). This phenomenon describes how the temperature-invariant parameters of transport rate equations (either Arrhenius prefactors or Eyring-Polanyi activation entropies) are strongly correlated with the slopes of temperature-variant parameters (activation energies). This behavior is well documented in polymeric systems involving polymer chain–limited solute transport (*58*, *59*) but has also been recently observed in hydrated polymer systems (*40*, *54*, *55*). Applying the linear free energy framework to the membrane effects measured in this study, Fig. 3D demonstrates that $∆E_{a}^{m-s}$ values are linearly correlated with $∆{\left(∆S^{‡}\right)}^{m-s}$ for monovalent ions. The positive slope of the trend shows that ions experiencing greater enthalpic barriers in the membrane also have a stronger entropic incentive for transport. A positively correlated linear free energy effect essentially dampens the observable impact of membrane interactions on ion motion because ∆*S*^‡^ and *E*_*a*_ affect ion transport rates in opposing directions (Eq. 2 and figs. S18 and S19). Thus, the two factors “compensate” for one another. The dampened differences demonstrate why it is so instructive to analyze rate processes through TST: Competing enthalpic and entropic effects can be effectively decoupled to assess molecular differences in ion transport behavior. Independently analyzed membrane and solution datasets [plotting ${\left(∆S^{‡}\right)}^{m}$ versus $E_{a}^{m}$ and ${\left(∆S^{‡}\right)}^{s}$ versus $E_{a}^{s}$, respectively] also exhibit compensation behavior (fig. S20); however, the correlation for each individual phase is weaker than the highly linear relationship shown in Fig. 3D.

### Phenomena driving ion-specific membrane interactions

The ion-specific values for $∆E_{a}^{m-s}$ and $∆{\left(∆S^{‡}\right)}^{m-s}$ demonstrate that these IEMs exhibit SIEs. However, in the absence of a mechanistic understanding or clear driving force behind these trends, it remains unclear how such SIEs might be leveraged in designing membranes for ion separations. The highly linear trend for monovalent ions in Fig. 3D suggests that a singular key phenomenon may be driving monovalent SIEs. Drawing on insights from both the polyelectrolyte literature (*16*–*18*) and the membrane literature (*32*, *34*, *54*), the remainder of this manuscript investigates three key phenomena that may contribute to ion-specific interactions with IEMs. In order of prevalence in the open literature, we test for contact ion pairs, ion dehydration, and solvent-mediated ion pairs in these IEMs. Because of the convoluted nature of membrane systems, these effects are typically evoked with indirect evidence. However, by leveraging both the experimental and simulated results, we attempt to probe each of these possibilities directly and rigorously.

#### Contact ion pairing in charged membranes

**Fig. 4.**
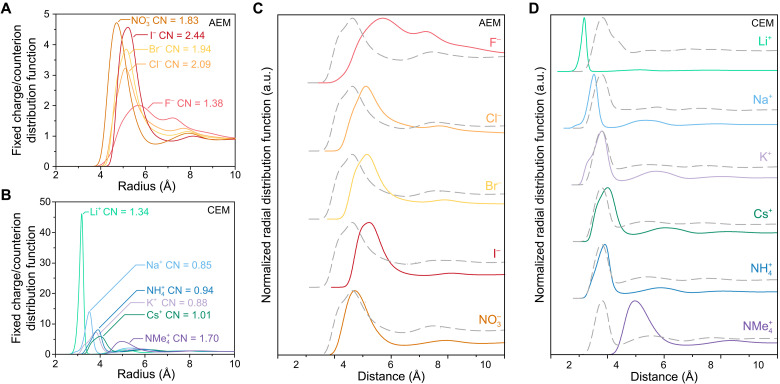
MD simulations of SIEs in charged membranes. Simulated RDFs [*g*(*r*)] showing the spatial distribution of mobile species relative to a central fixed charge group at room temperature. (**A**) RDFs for RNMe_3_^+^ fixed charge groups and anionic counterions. (**B**) RDFs for RSO_3_^−^ fixed charge groups and cationic counterions. For each counterion form, the primary coordination number of the fixed charge group, defined as the number of ions present at a separation distance less than the first local minimum of the density profile, is listed. (**C** and **D**) Normalized RDFs, with the largest peak height set to unity, for counterions (colored, solid lines) and water molecules (gray, dashed lines) around the fixed charge groups. Un-normalized water RDFs are presented in fig. S21. These overlays help demonstrate the competition between ions and water molecules for direct interaction with the fixed charge groups. a.u., arbitrary units.

The formation of contact ion pairs between mobile counterions and fixed charge groups is often invoked to explain ion transport results in IEMs (*34*, *36*, *38*, *54*, *60*, *61*). Contact ion pairs refer specifically to direct ion/ion neighbors, with no intervening solvent, such that the Coulombic attractions are felt at full strength. MD simulations can readily assess ion-specific interactions between fixed charge groups and mobile counterions by extracting the radial distribution functions (RDFs) and coordination profiles of these two species (*62*–*64*). On the basis of the OPLS force fields used in our methods, we expect the simulated membranes to be suitable for qualitatively assessing the relative prevalence of contact ion pairing between different mobile counterions. Figure 4 (A and B) shows the RDFs and nearest-neighbor coordination numbers of counterions relative to the fixed charge groups ($CN_{A-g}^{\mathrm{IEM}}$) in AEMs and CEMs. These distribution functions are directly comparable because the ratio of fixed charge groups to counterions is identical in each simulation.

In the AEMs, NO_3_^−^ achieves the closest distance of approach to the RNMe_3_^+^ groups, whereas F^−^ maintains the greatest separation among all the anions. For each anion, $CN_{A-g}^{\mathrm{IEM}}>1$, reaching values as high as 2.44 for I^−^. A coordination number of 1 suggests a singular cation/anion interaction, whereas a value of 2 indicates that two counterions are nominally equidistant from each fixed charge group. Consequently, $CN_{A-g}^{\mathrm{IEM}}∼2$ implies that the anions are positioned nearly equidistant between two RNMe_3_^+^ groups because of electroneutral mass balance. These results reveal that F^−^ uniquely adopts a more pairwise interaction with RNMe_3_^+^ at a comparatively larger distance, whereas other anions are located closer, typically bridging two or three RNMe_3_^+^ groups. However, for all anions measured, water molecules achieve closer distances of approach to RNMe_3_^+^ (Fig. 4C), suggesting that these ion/ion interactions do not occur within each other’s first coordination shells. That is, the anions do not appear to form contact ion pairs with the fixed charge groups.

The RDFs of the cations exhibit markedly different trends compared to those of the anions (Fig. 4B). Similar to the anions, NMe_4_^+^ and Cs^+^ RDFs peak at 2× to 5× their mean-field densities. However, NH_4_^+^, K^+^, Na^+^, and particularly Li^+^ cluster near the fixed charge groups at substantially higher densities. Notably, the Li^+^ RDF reaches a density 45× greater than the mean-field density. Most cations exhibit $CN_{A-g}^{\mathrm{IEM}}≅1$, indicating limited shared interactions between multiple RSO_3_^−^ and the same counterion. Small populations of certain cations, like Na^+^ and K^+^, are distinctly far from their nearest RSO_3_^−^ group (evidenced by having ${\mathrm{CN}}_{A-g}^{\mathrm{IEM}}<1$). However, both NMe_4_^+^ and Li^+^ exhibit ${\mathrm{CN}}_{A-g}^{\mathrm{IEM}}>1$. Whereas the NMe_4_^+^/RSO_3_^−^ interactions resemble the coordination behavior of RNMe_3_^+^ with mobile anions, the coordination of Li^+^ is distinct. The notably higher densities for Li^+^ reflect coordination dominated by favorable energetics near the fixed charge groups. The Li^+^ RDF therefore indicates a highly stabilized structure, sometimes involving multiple RSO_3_^−^ groups; this signature of intimate paired interactions completely dominates the Li^+^ RDF. Unique to Li^+^-form CEMs, the first shell of waters around the RSO_3_^−^ groups extends toward greater distances (Fig. 4D), which is consistent with the detection of ion pairs or even ion clusters.

The Na^+^ RDF features a small shoulder at the same location as Li^+^, suggesting that some Na^+^ counterions may adopt a similar configuration. RDFs of larger bare cations also exhibit low-distance shoulders, which could indicate a small population of strongly interacting fixed charge group/counterion pairs. These populations are more clearly interpreted when compared against water RDFs in Fig. 4D. Na^+^, K^+^, Cs^+^, and NH_4_^+^ each show two populations of ions within the first peak, and one of these populations always overlaps with the location of the water RDF peak. Because these ions do not allow room for intervening waters, these RDFs suggest that these cation populations are entirely contact ion paired. This assignment is supported by applying the Bjerrum criterion for ion pairing (*53*, *65*) to the potential of mean force (PMF) extracted from each energy profile (*66*) (fig. S22). Bjerrum calculated that any set of ions having an interaction potential greater than $2k_{B}T≅5$ kJ/mol would be considered an ion pair. Critically, each cation identified via the positional argument meets the Bjerrum criterion, whereas the other mobile ions do not.

This analysis suggests that over 80% of each cation (except NMe_4_^+^) participates in a contact ion pair. However, the true population of contact ion pairs in the experimental systems may be much smaller. The OPLS force field is known to overpredict contact ion pair formation (*66*–*68*), warranting some caution in interpreting these results quantitatively. However, numerous studies have demonstrated that the qualitative trends can be reliable, even if the absolute prevalence of contact ion pairing is overestimated (*66*–*71*). Therefore, the key result from these MD simulations is the trend among cations. Because OPLS typically overpredicts contact ion pairs, it is reasonable to conclude that these simulations predict no contact ion pairing in the AEM system. For the CEMs, we can conclude that the tendency to form contact ion pairs is ranked in the order of Li^+^ > Na^+^ > K^+^ ~ Cs^+^ ~ NH_4_^+^. On the basis of the magnitude of the RDF peak, Li^+^ pairs may be approximately 4× more favorable than Na^+^, which may be approximately 2× more favorable than the remaining cations.

To complement the MD simulations, we performed an experimental investigation of contact ion pairing using in situ vibrational spectroscopy. Raman spectroscopy has demonstrated sensitivity to contact ion pairs and is among the most common methods to detect this structure in ion solutions (*72*–*75*). Infrared (IR) spectroscopy can similarly detect contact ion pairing, provided that a suitable vibrational mode is present in the molecule. These methods have been shown to be reliable not only in detecting the presence of ion pairs but also in assessing their prevalence quantitatively, making this dataset extremely valuable alongside the qualitative simulation results. Ion pairing is detectable via vibrational spectroscopy because the formation of contact ion pairs shifts the vibrational energy of bonds within the paired structures, allowing the detection and quantification of distinct populations. By using both Raman and IR spectroscopy, we searched for signatures of ion pairing in functional groups active in both IR and Raman spectroscopy (*76*). Then, using Gaussian deconvolution (section S2.1), we identified coordination structures associated with fixed charge groups in various nearest-neighbor configurations.

Assigning deconvoluted peaks to specific physical phenomena in the membranes is inherently challenging. However, we increased confidence in our interpretation by comparing the spectra of hydrated and dried membranes for each counterion form. Drying out the membrane promotes contact ion pair formation as the absence of water shifts the chemical equilibrium toward the paired state. Once paired, the strong electrostatic interactions between counterions and fixed charge groups induce peak shifts, reflecting changes in Raman- and IR-active vibrational modes. Assessing relevant conformations (figs. S23 and S24) produced by density functional theory (DFT), we predicted red shifts and blue shifts of the fixed charge group vibrational modes. These DFT simulations predicted bidentate pairing uniquely present in the Li^+^ system, in which multiple fixed charge groups cluster around a single cation. This observation is consistent with the MD simulations, as discussed previously (Fig. 4B). The DFT results can be qualitatively rationalized via the size and mass of the counterions (fig. S25), providing additional confidence that the spectra were analyzed reliably and were sufficiently sensitive to the formation of contact ion pairs.

**Fig. 5.**
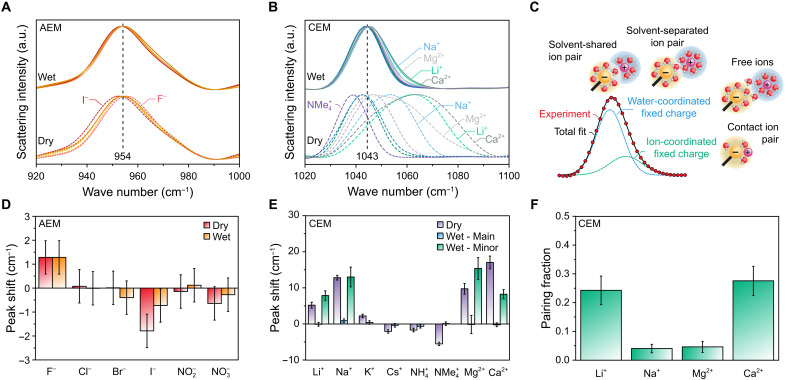
Ion pairing in charged membranes. Raman spectra of fixed charge group vibrations in hydrated and dried membranes. (**A**) C─N stretch of RNMe_3_^+^ in the AEMs. (**B**) The S─O symmetric stretch of RSO_3_^−^ in the CEMs. Notable counterion spectra are labeled, whereas overlapping results are unlabeled for clarity. (**C**) Illustration of Gaussian deconvolution used to determine populations of paired sulfonate species. Contact ion pairs produce a distinct peak in Raman spectroscopy, whereas solvent-separated and solvent-shared ion pairs are indistinguishable from free ions. (**D** and **E**) Bond vibrational energies isolated via Gaussian deconvolution for (D) the C─N stretch in the AEMs and (E) the S─O symmetric stretch in the CEMs, referenced against the average location of the primary signal in hydrated membranes. (**F**) Fraction of RSO_3_^−^ groups calculated to form contact ion pairs for each counterion form. The error bars in (D) to (F) were calculated using the bootstrapping techniques detailed in section S2.8 and fig. S31, followed by standard error propagation methods.

Given the established precedence for detecting contact ion pairs via Raman spectroscopy, these spectra are presented and discussed in the main text, whereas the corresponding IR spectra are provided in fig. S26. Full spectra are shown in figs. S27 and S28. Notably, SO_4_^2−^ counterions exhibit a Raman vibrational mode near the analyzed RNMe_3_^+^ peak, so these spectra were excluded from the following analysis (cf. fig. S29). The dry membrane spectra in Fig. 5 (A and B) demonstrate that the location of the RNMe_3_^+^ peak in AEMs and RSO_3_^−^ peak in CEMs depends on the membrane counterion form. This trend is more pronounced in the CEMs but is distinctly present in both types of IEMs. As expected, densely charged ions like Li^+^ and F^−^ cause high-energy shifts, whereas larger bare ions like NMe_4_^+^ and I^−^ cause low-energy shifts. The corresponding vibrational energies calculated via Gaussian deconvolution (Fig. 5C) are presented in Fig. 5 (D and E). For clarity, the peak locations are referenced against the average vibrational energy of the water-coordinated fixed charge groups. The statistically significant vibrational shifts observed in the dry membranes provide confidence that our experiments are sensitive to ion pairing in these membranes. However, the deconvoluted peaks also highlight a limitation of this analysis: For moderately sized ions, the effects of mass and electrostatic interactions balance one another, leading to signals for paired fixed charge groups that are comparable to those of unpaired fixed charge groups. Consequently, this spectroscopic analysis is not sensitive to contact ion pairs for moderately sized ions (e.g., Br^−^ or NH_4_^+^), although such pairs may still be present.

After calibrating the ion-pairing analysis using dry membrane spectra, we examined the spectra of hydrated IEMs for signals indicative of contact ion pairs in the presence of water. For the hydrated AEMs, only a single C─N peak was observed (fig. S30). As shown in Fig. 5D, this C─N peak exhibited minimal variation across counterions compared to the differences seen in the spectra of dried AEMs. Given the small variations in its position, this peak corresponds to water-coordinated RNMe_3_^+^, and the results do not provide statistically significant evidence of contact ion pairing in these AEMs. In contrast, the spectra of hydrated CEMs with Li^+^, Na^+^, Mg^2+^, and Ca^2+^ counterions required Gaussian fitting with two distinct fixed charge group peaks (fig. S30). Evidence of this second peak is apparent in the high-energy shoulders visible in the hydrated spectra shown in Fig. 5B. The presence of a significant second peak indicates that two configurations of first-shell coordination interactions were detected for RSO_3_^−^ groups in these CEMs. The lower energy peak position for these four cations remained constant and aligned with the single peak detected in the spectra of other cation forms (Fig. 5E), suggesting that it corresponds to water-coordinated RSO_3_^−^. In contrast, the higher energy peaks observed in the Li^+^, Na^+^, Mg^2+^, and Ca^2+^ form spectra varied across the counterions, following the trend of contact ion pairing vibrational energies established in the dried CEM spectra. This second peak is therefore assigned to RSO_3_^−^ groups involved in contact ion pairing.

Figure 5F presents the fraction of RSO_3_^−^ groups that form a contact ion pair, calculated as the fractional area of the corresponding Gaussian peaks. In each CEM, fewer than one-third of RSO_3_^−^ groups participate in contact ion pairing. However, because divalent counterions electrically balance two fixed charge groups, the impact on counterions may be underrepresented. Assuming pairwise ion pairing, which provides the highest estimate of paired counterions, up to ~60% of Ca^2+^, ~25% of Li^+^, ~10% of Mg^2+^, and ~5% Na^+^ ions were calculated to participate in contact ion pairs. The presence of contact ion pairs involving multiple RSO_3_^−^ groups coordinating the same counterion would result in a lower fraction of paired counterions. When these qualitative results are compared with the transport data in Fig. 3D, notable trends emerge. Specifically, Li^+^, Mg^2+^, and Ca^2+^ exhibited a negative deviation from the compensation trend that captured the transport results of the other cations. This negative deviation indicates that these ions exhibit unusually high activation enthalpies, unusually low activation entropies, or a combination of the two. These vibrational spectroscopy results provide a potential explanation for this behavior: Contact ion pairing adds a strong Coulombic force to be overcome and implies having fewer oriented waters to liberate upon ion transport, which are respectively consistent with the observed enthalpic and entropic deviations.

The spectroscopic ion pairing analysis presented above has certain limitations. Specifically, for membranes with moderately sized counterions, contact ion pairing peaks could be obscured by the peaks corresponding to the hydrated fixed charge group. These limitations are mitigated by considering the vibrational spectroscopy results in tandem with the MD simulations. The qualitative results of these independent approaches align remarkably well. Both analyses indicate that Li^+^ exhibits a markedly higher tendency to coordinate with RSO_3_^−^ groups than other monovalent cations in the CEMs, with a smaller signal (4× smaller in the simulations and 5× smaller in the experiments) of Na^+^ achieving similarly close conformations. Meanwhile, neither approach detected substantial contact ion pairing for any of the monovalent anions. The simulations using OPLS force fields predict a greater prevalence of contact ion pairing than we measured experimentally, which is consistent with previous reports on the topic (*66*–*68*). The convergence of these analyses generates confidence in their interpretations. We conclude that contact ion pairing can occur in these IEMs, potentially influencing the energetics of monovalent ion transport; however, most monovalent counterions do not exhibit substantial contact ion pairing detectable using these methods. Therefore, the ion-specific energetics of monovalent ion transport in these IEMs are not primarily governed by contact ion pairing. In contrast, the transport rates of multivalent ions appear to be substantially influenced by contact ion pairing.

#### Ion dehydration in charged membranes

Ion dehydration upon partitioning into membranes has also been invoked to explain SIEs on transport phenomena (*39*, *54*, *77*, *78*). To evaluate the hydration of mobile counterions in the simulated membranes, we examined RDFs of ion/water interactions. Complete RDFs and interaction energy data are provided in figs. S32 and S33. However, differences in IEM water content and the infinite dilution conditions preclude direct graphical comparisons of these RDFs. Instead, Table 1 summarizes the key results of the ion/water RDFs as coordination numbers. Before interpreting these results, it is important to distinguish between the hydration shell and the coordination shell of an ion. In the vicinity of an ion, certain water molecules are strongly oriented by the electrostatic field of the ion, forming the hydration shell (sometimes referred to as the dynamic or primary hydration shell for clarity) (*18*, *53*, *65*). Other nearby water molecules contribute less stabilizing energy toward ion solvation and are only considered part of the ion coordination shell but not the ion hydration shell. All hydrating waters are considered part of the coordination shell, but not all coordinating waters are dynamically hydrating. Although the loss of a coordinating water may not affect ion motion, the loss of a hydrating water should markedly affect ion interactions and transport.

**Table 1.** Water coordination and hydration numbers. The average dynamic hydration number of ions in aqueous solution ($HN_{g-w}^{\mathrm{ID}}$) as reported by Marcus (*18*) listed alongside simulation results. Simulated values include the primary coordination number of water for each counterion ($CN_{g-w}^{j}$), where j=ID for infinite dilution and j=IEM for the simulated IEMs; the primary coordination number of water for the fixed charge groups ($CN_{A-w}^{\mathrm{IEM}}$) in IEMs of each counterion form; and the total membrane hydration number in the simulation (λ). The IEM water contents were fixed to match the experimental values (section S3.2.2). Corresponding RDFs are provided in figs. S21, S32, and S33. Values for ${\mathrm{CN}}_{g-w}^{\mathrm{ID}}$ are compared against literature values compiled by Marcus (*18*) in table S13.

Ion	${\text{HN}}_{g-w}^{\text{ID}}$ (*18*)	${\text{CN}}_{g-w}^{\text{ID}}$	${\text{CN}}_{g-w}^{\text{IEM}}$	${\text{CN}}_{A-w}^{\text{IEM}}$	λ
F^−^	4.2	6.2	6.1	23.7	24.0
Cl^−^	2.0	6.7	6.0	22.2	19.0
Br^−^	1.3	7.1	5.9	21.0	17.3
I^−^	0.9	7.4	5.4	19.2	13.9
NO_3_^−^	1.6	10.1	6.7	20.0	16.7
Li^+^	4.2	4.1	2.6	9.7	20.5
Na^+^	3.3	5.9	4.7	8.9	19.9
K^+^	2.3	7.0	5.6	7.4	18.9
Cs^+^	1.7	8.3	6.7	6.7	18.3
NH_4_^+^	1.9	7.0	5.5	6.6	19.0
NMe_4_^+^	2.1	31.5	21.4	6.0	17.7

Ions with strong electrostatic fields tend to be small and have lower surface areas, resulting in an inverse relationship between the number of hydrating waters (*HN*) and number of coordinating waters (*CN*) (*18*, *53*, *65*). This leads to a picture of densely charged ions generating strong electric fields that orient a substantial fraction of adjacent water dipoles; contrastingly, larger ions exerting weaker electric fields across their greater surface areas allow a greater number of water molecules to interact with the ion loosely as part of a coordination shell. MD simulations enable the calculation of *CN* values via RDF analyses, but *HN* values are not easily accessible by MD simulations. However, through careful analysis and appropriate controls, trends in *CN* can be leveraged to infer qualitative insights into the hydration of ions within the IEMs.

To emphasize the difference between ion hydration and coordination, the first two columns of Table 1 compare reported infinite dilution hydration numbers ($HN_{g-w}^{\mathrm{ID}}$) to simulated infinite dilution coordination numbers ($CN_{g-w}^{\mathrm{ID}}$). As expected, the simulated coordination numbers exceed the experimental hydration numbers ($CN_{g-w}^{\mathrm{ID}}>HN_{g-w}^{\mathrm{ID}}$). Furthermore, as the surface area of the bare ion increases, ${\mathrm{HN}}_{g-w}^{\mathrm{ID}}$ decreases, whereas ${\mathrm{CN}}_{g-w}^{\mathrm{ID}}$ increases, consistent with the previous discussion. This diverging trend does not persist for the coordination number of counterions in the simulated IEMs, ${\mathrm{CN}}_{g-w}^{\mathrm{IEM}}$. The polymer matrix displaces water molecules surroundings the ions (on average, $\phi_{w}≅0.54$), leading to a general reduction of coordinating waters such that ${\mathrm{CN}}_{g-w}^{\mathrm{IEM}}<{\mathrm{CN}}_{g-w}^{\mathrm{ID}}$. The location of these coordinating waters does not notably change (fig. S34). Yet, a different trend emerges for the ${\mathrm{CN}}_{g-w}^{\mathrm{IEM}}$ values for alkali halide ions. In CEMs, cations maintain the infinite dilution trend of Cs^+^ > K^+^ > Na^+^ > Li^+^. However, in AEMs, the infinite dilution trend for the anions is inverted, with ${\mathrm{CN}}_{g-w}^{\mathrm{AEM}}$ ordered as F^−^ > Cl^−^ > Br^−^ > I^−^. This inversion is similar to that observed for the transport rate trends for the AEM (Fig. 3). Two noteworthy direct comparisons can be made between the IEMs and infinite dilution simulations: (i) F^−^ maintains a coordination shell in the AEM that is nearly identical to its infinite dilution value (6.2 and 6.1, respectively), supporting the relatively weak fixed charge group interactions observed for F^−^ in Figs. 4 and 5. (ii) Li^+^ loses coordinating waters such that ${\mathrm{CN}}_{g-w}^{\mathrm{IEM}}<{\mathrm{HN}}_{g-w}^{\mathrm{ID}}$ (2.6 < 4.2), providing clear evidence of dehydration. These *CN* results suggest that F^−^ is expected to retain the most solution-like behavior within the IEM, whereas Li^+^ is expected to display the strongest signs of dehydration.

The availability of water in the simulated IEMs is also influenced by the fixed charge groups. The final two columns of Table 1 compare the number of coordinated waters and the total number of waters per fixed charge group in each IEM ($CN_{A-w}^{\mathrm{IEM}}$ and λ, respectively). For RNMe_3_^+^ in the AEMs, the number of coordinating waters exceeds the total waters available: ${\mathrm{CN}}_{A-w}^{\mathrm{IEM}}>\lambda$. A similar result is observed for the NMe_4_^+^ form CEMs, where ${\mathrm{CN}}_{g-w}^{\mathrm{IEM}}>\lambda$. To reconcile this apparent discrepancy in the mass balance of water, we conclude that some coordinating water molecules must be shared between the bulky methylammonium groups in IEMs. This constrained distribution suggests that waters coordinating mobile anions are likely shared with the RNMe_3_^+^ fixed charge groups. The RNMe_3_^+^ RDF shows two distinct peaks within the primary coordination shell (fig. S21), which could correspond to these different populations of shared waters. For the CEMs, water sharing between fixed charge groups appears unlikely, as RSO_3_^−^ groups coordinate only 6 to 10 waters, resulting in ${\mathrm{CN}}_{A-w}^{\mathrm{IEM}}<\lambda$. The largest ${\mathrm{CN}}_{A-w}^{\mathrm{CEM}}$ is observed in the Li^+^ form CEM, indicating that Li^+^ is not dehydrated due to a lack of available space or water. Instead, these results suggest that Li^+^ ions release hydration waters to assemble a more favorable coordination shell. This observation aligns with the conclusion from the previous section regarding contact ion pair formation and the prevalence of clustered ion pairs with an expanded host of nearby waters.

**Fig. 6.**
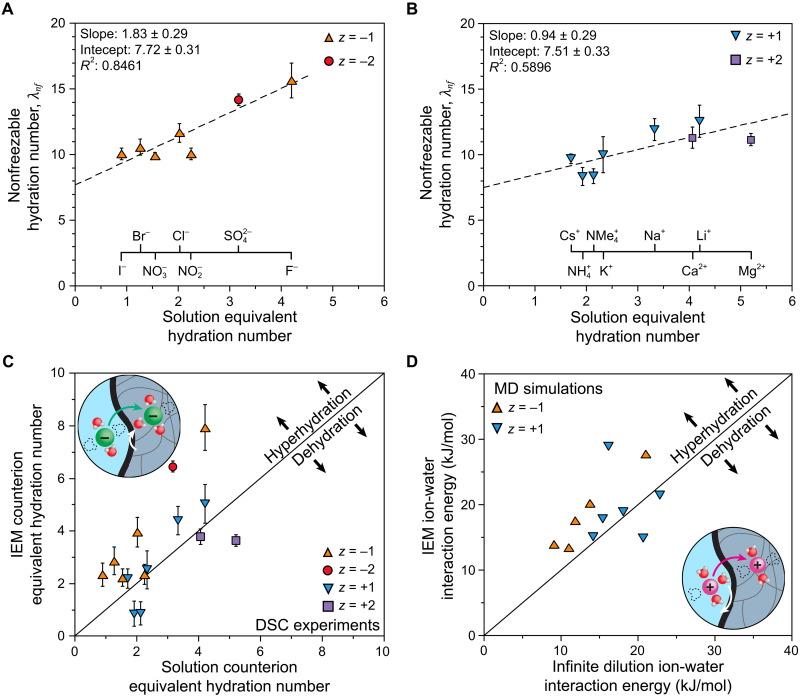
Ion hydration in charged membranes. The nonfreezable hydration number of the membrane ($\lambda_{\mathrm{nf}}$) plotted against the equivalent hydration number of the counterion (${\mathrm{HN}}_{g-w}^{\mathrm{ID}}/|z_{g}|$) for (**A**) anions in the AEM and (**B**) cations in the CEM. The position of individual ions is indicated on a second *x* axis. Uncertainties represent the SD calculated using standard error propagation methods. (**C**) Parity plot comparing the apparent counterion equivalent hydration number in the membrane (${\mathrm{HN}}_{g-w}^{\mathrm{IEM}}/|z_{g}|$) with the tabulated counterion equivalent hydration number in dilute aqueous solution (${\mathrm{HN}}_{g-w}^{\mathrm{ID}}/|z_{g}|$). (**D**) Parity plot comparing a proxy for counterion/water interactions, normalized per counterion, in the IEM and infinite dilution simulations. Insets illustrate that ions may gain or lose hydrating waters as they partition into an IEM, although they always lose coordinating waters.

To compare the simulation analysis with comparable experimental data, we used differential scanning calorimetry (DSC) to assess the interactions of water molecules within the membranes in situ (*36*, *79*–*81*). DSC characterizes the hydration environment of ions by analyzing the states of water in the membrane. Water in IEMs can be divided into two populations: freezable and nonfreezable. Strongly oriented ion hydration shells correspond to nonfreezable water, whereas weakly coordinated and noncoordinated waters are typically freezable (*82*, *83*). Nonfreezable water is evident from the reduced enthalpy observed in the water melting peak of the membrane (fig. S35). The observed enthalpy integrates to a lower value than that predicted by the water content of the membrane and the melting enthalpy of bulk water. Referencing the measured DSC results against this prediction, the membrane hydration number can be separated into its freezable and nonfreezable components: $\lambda =\lambda_{f}+\lambda_{\mathrm{nf}}$ (table S14). Interpreting $\lambda_{\mathrm{nf}}$ can be challenging due to contributions from waters bound to nonionic hydrophilic moieties in polymer membranes, such as the alcohols and esters present in these IEMs. Thus, it is likely that not all nonfreezable waters are part of the primary hydration shell of an ion. To isolate the contribution of mobile ions, we examined the trend of $\lambda_{\mathrm{nf}}$ for the same membrane in different counterion forms. Specifically, plotting $\lambda_{\mathrm{nf}}$ against the equivalent hydration number, $HN_{g-w}^{\mathrm{ID}}/|z_{g}|$, of each counterion decouples the counterion contribution into the slope, whereas the extrapolated *y* intercept reflects hydration contributions from the polymer backbone and fixed charge groups. Hydration results decoupled in this manner are presented in Fig. 6 (A and B).

The states of water analyses for both AEMs and CEMs produced reasonably linear trends between $\lambda_{\mathrm{nf}}$ and ${\mathrm{HN}}_{g-w}^{\mathrm{ID}}/|z_{g}|$, indicating that the extent of counterion hydration in the membranes is continuous across the range of ions studied. For cations (Fig. 6B), the slope is close to unity, suggesting that ions essentially retain their infinite dilution hydration in the IEM. In contrast, the slope for the anions (Fig. 6A) exceeds unity, indicating that an increase in ${\mathrm{HN}}_{g-w}^{\mathrm{ID}}/|z_{g}|$ corresponds to nearly twice that increase in $\lambda_{\mathrm{nf}}$. These differences align with the coordination results from the simulations, where CEMs preserve infinite dilution behavior, but AEMs exhibit deviations. The analysis of Table 1 suggested that AEMs likely foster shared coordination shells between counterions and fixed charge groups, which are consistent with the AEM DSC results. Oppositely charged ions can cooperate to orient waters in the same direction, water dipoles pointing toward the cation and away from the anion. We hypothesize that these shared, more strongly oriented waters may become nonfreezable, resulting in the observed slope greater than unity in Fig. 6A.

The fixed charge group hydration contributes to the *y* intercept of each plot, which represents $\lambda_{\mathrm{nf}}$ in the absence of counterion contributions. Because both the AEM and CEM share the same methacrylate polymer backbone and similar mol fractions of charged monomer and cross-linker, the contribution of nonionic hydrophilic moieties should be comparable between the membranes. This similarity allows us to focus on differences in the hydration numbers of RNMe_3_^+^ and RSO_3_^−^, the sole structural difference between the AEM and CEM. We hypothesize that RNMe_3_^+^ and NMe_4_^+^ likely have similar hydration numbers (${\mathrm{HN}}_{A-w}^{\mathrm{AEM}}≅2$) due to their structural similarity. Conversely, the closest common ionic analog for RSO_3_^−^ is SO_4_^2−^. However, SO_4_^2−^ has two formal charges and four pendant oxygens (two charge-carrying oxygens per formal charge), compared to the single formal charge and three pendant oxygens of the RSO_3_^−^ group (three charge-carrying oxygens per formal charge), making the CEM fixed charge groups more charge-delocalized and less hydrophilic than SO_4_^2−^. Consequently, it is reasonable to expect that RSO_3_^−^ would have a lower equivalent hydration number than SO_4_^2−^ (${\mathrm{HN}}_{A-w}^{\mathrm{CEM}}<3$). Experimentally, we observe that the *y* intercepts for the AEM and CEM are similar (7.72 ± 0.31 and 7.51 ± 0.33, respectively), suggesting that the hydration number of RSO_3_^−^ is not appreciably different from that of RNMe_3_^+^. That is, these experimental results are consistent with ${\mathrm{HN}}_{A-w}^{\mathrm{CEM}}≅2$. Given the charge-delocalization difference between RSO_3_^−^ and SO_4_^2−^, this result appears reasonable. Having established confidence in the *y* intercepts of these plots, we can subtract them from $\lambda_{\mathrm{nf}}$ to isolate the equivalent hydration number of the counterions in the IEMs, ${\mathrm{HN}}_{g-w}^{\mathrm{IEM}}/|z_{g}|$.

To compare the experimental and simulation hydration analyses, it is important to normalize each dataset appropriately to reveal qualitative trends. To achieve this, we constructed membrane/solution parity plots for both experimental and simulated data. The values for ${\mathrm{HN}}_{g-w}^{\mathrm{IEM}}/|z_{g}|$ extracted from the states of water analysis can be directly compared to the corresponding values in aqueous solution, ${\mathrm{HN}}_{g-w}^{\mathrm{ID}}/|z_{g}|$, as shown in Fig. 6C. For the simulations, we assessed ion hydration rather than ion coordination. To this end, the PMF for water molecules within the first coordination shell of counterions was extracted from the RDFs of counterions both in the membrane and at infinite dilution (Fig. 6D; see calculation details in section S3.3.2). Although these values are not hydration numbers per se, incorporating the strength of interaction effectively emphasizes hydration interactions over coordination interactions. This approach allows us to compare simulated hydration interactions, providing a more nuanced perspective than focusing solely on coordination interactions.

The coupled parity plots demonstrate strong agreement between the experimental and simulation hydration analyses. Although each individual process involves assumptions that could complicate their interpretation, the consistency between these independent analyses lends credibility to the results. Cation data points generally align closely with the parity lines, suggesting that cations largely retain their hydration shells in these CEMs. In contrast, anion data points generally fall above the parity lines, indicating that hydration interactions in the AEMs are stronger than in aqueous solution. The trends in Fig. 6 (C and D) are consistent with the coordination-based hydration analysis derived from Table 1. Overall, dehydration does not appear to be a prevalent phenomenon in these IEMs. Instead, anions in the AEMs exhibit enhanced hydration interactions, whereas cations in CEMs show minimal changes in hydration behavior. Although both anions and cations coordinate fewer waters in the IEM than in external aqueous solution (Table 1), these fewer coordination interactions are, on average, stronger in the IEMs.

#### Solvent-mediated ion pairing in charged membranes

Solvent-mediated ion pairing refers to configurations in which one (solvent-shared ion pairs) or two (solvent-separated ion pairs) water molecules intervene between oppositely charged ions (*84*). This stands in stark contrast to contact ion pairs, which have no water molecules between the ions. As a consequence of intervening water molecules, solvent-mediated ion pairs typically exhibit weaker Coulombic interactions. Nevertheless, solvent-mediated ion pairs are not equivalent to fully dissociated ions and are increasingly recognized as a primary mode of interaction between ions in solution (*16*, *84*). The unexpected presence of additional water interactions within the IEMs, especially for anions, suggests that such structures are relevant in this IEM system. Unfortunately, the looser definitions involved in solvent-mediated ion pairing (e.g., how far can a water molecule be from the central line between two ions and still be “intervening”) complicates any attempt to define discrete categories of ion pairs. Instead, we quantified this continuum of ion association through a graded measure of interaction strength, acknowledging that some solvent-mediated ion interactions might be weak, whereas others might approach the interaction strength of contact ion pairs.

**Fig. 7.**
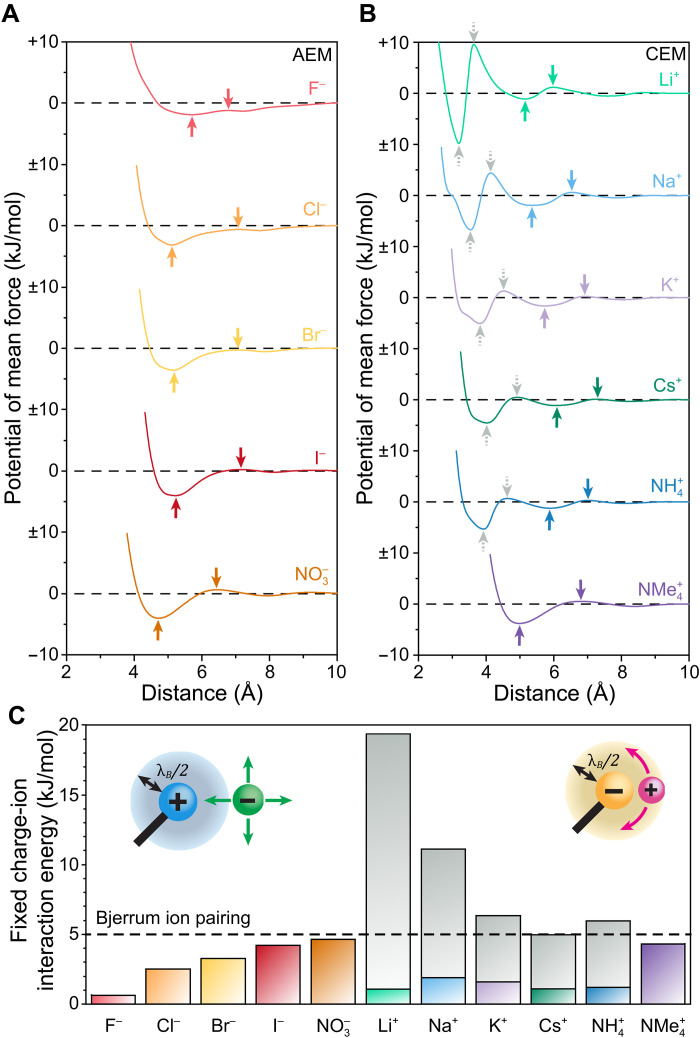
Quantifying fixed charge group/ion interactions via MD simulations. PMF profiles for the interactions between the (**A**) anions and (**B**) cations with the fixed charge groups at 27°C as a function of separation distance. Extrema previously identified as contact ion pairs are marked with gray dashed arrows. Extrema now interpreted as solvent-mediated ion pairs are marked with colored solid arrows. (**C**) Extent of interaction associated with the ion pair, i.e., the difference between the relevant minimum and maximum potentials, for the mobile ions and fixed charge groups. The dashed line indicates the ion pairing threshold proposed by Bjerrum, $2k_{B}T$, which is ~5 kJ/mol at 27°C. The gray bars represent the contact ion pairs, whereas the colored bars represent the solvent-mediated ion pairs. Insets illustrate ion pairing as described by Bjerrum: Although ions generally diffuse somewhat independently, energy barriers greater than $2k_{B}T$ limit ion diffusion and produce an ion pair. In Bjerrum’s formalization of ion pairing, the criterion for ion pairing is entirely Coulombic, corresponding to a separation distance less than half of a Bjerrum length, $\lambda_{B}/2$.

To determine the most relevant energy barrier for each ion, we analyzed its PMF profile relative to the fixed charge groups. After excluding populations corresponding to contact ion pairs (cf. Fig. 4 and fig. S22), we measured the energy difference between the next-nearest potential minimum and the adjacent maximum at a slightly greater distance from the fixed charges. This approach isolates the energy barrier associated with the departure of the second ion population from the referenced fixed charge group (*66*). As before, we draw analogy to Bjerrum’s ion pairing criterion, which was shown to reliably identify contact ion pairs. Solvent-mediated ion pairs are challenging to detect experimentally, so this simulation-based method is our only means of quantifying them. However, given the strong agreement between the experimental and simulation results in detecting ion hydration and contact ion pairing, we view the fidelity of this analysis as sufficiently robust. Figure 7 (A and B) presents the PMF profiles derived from each fixed charge/counterion RDF, with arrows indicating the extrema used in the calculations.

The PMF profiles in Fig. 7 (A and B) provide insights into the mechanism involved in separating the mobile ions from the fixed charges. Each profile exhibits a rise in energy as the ions are displaced from a local minimum, reflecting the loss of favorable interactions which result in the lowered density observed in the corresponding RDFs (Fig. 4). For all anions, as well as cations in solvent-mediated pairs, the potential shift almost entirely reflects this step of ion dissociation: The local maximum occurs near the mean-field reference potential of zero, indicating minimal penalty for separation. In contrast, cations forming contact ion pairs exhibit a notably higher local maximum, well into the positive range. We hypothesize that this unfavorable positioning reflects the energetic cost of reorganizing the more structured hydration shells required once the ions cease to neutralize each other. Because solvent-mediated ion pairs do not shed their hydration shell, the water molecules do not become immobilized upon ion pair separation.

The strength of solvent-mediated interactions, inferred from the preference given to these conformations (Fig. 7C), illustrates how cation behavior shifts when contact ion pairs are excluded. Although the Li^+^ contact ion pairs are extremely prevalent (and therefore energetically favorable), the second population of Na^+^, in solvent-mediated pairs, is larger than that of Li^+^. Notably, NMe_4_^+^, a counterion that does not form contact ion pairs, stands out with the highest rate of detection in a solvent-mediated ion pair. Among the anions, no contact ion pair formation was detected. Instead, F^−^ displays minimal preference between various conformations, whereas I^−^ and NO_3_^−^ are located in solvent-mediated pairs at a rate suggesting interactions nearly as strong as those seen in contact ion pairs. These interaction extents closely mirror the experimental trends in activation energy and entropy differences presented in Fig. 3. Although this correlation does not definitively establish causation, the alignment strongly suggests that solvent-mediated ion pairing is the most plausible driving force behind the observed ion-specific transport energetics among the three phenomena evaluated in this study.

### Correlating SIEs to ion properties

The phenomenological investigation presented above indicated that neither contact ion pairing nor ion dehydration adequately account for the SIEs observed in these IEMs. Instead, solvent-mediated interactions, where ions co-orient shared water molecules, emerged as the most plausible mechanism. However, the underlying origin of these ion-specific interactions remains unclear. In this section, we adopt a more traditional correlative SIE analysis to identify ion properties that align with the observed SIE trends (*16*–*18*). These results and the accompanying SIE theories can then be interpreted in the context of the phenomenological framework developed above. Given the strong correlation between the two metrics of transport energetics for the monovalent ions under study (Fig. 3D), we focus this analysis on explaining the observed variation in $∆E_{a}^{m-s}$ values for monovalent ions.

**Fig. 8.**
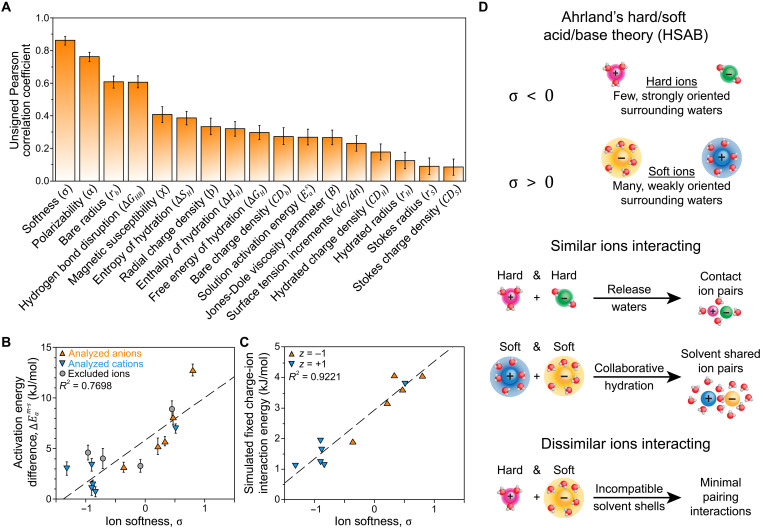
Correlating SIEs to ion properties. (**A**) Magnitude of Pearson correlation coefficients from a pairwise analysis of the activation energy difference, $∆E_{a}^{m-s}$, with each ion property considered in this study (table S15). Uncertainties were calculated via a 10,000-point Parametric bootstrap (section S2.8). Comparable Spearman correlation coefficients are given in fig. S36. (**B**) $∆E_{a}^{m-s}$ plotted against ion softness, σ, for the ions considered in this study. Ions excluded from the statistical analysis (NO_2_^−^, SO_4_^2−^, Mg^2+^, and Ca^2+^) are shown as gray data points. Uncertainties were calculated using standard error propagation methods based on standard errors of the activation energies. (**C**) Simulated ion interactions associated with solvent-mediated ion pairs plotted against σ for the ions simulated in this study. (**D**) Illustration of the ion pairing phenomena described by Ahrland in HSAB theory (*41*).

There are a wide range of theories and ion properties that have been proposed to explain SIEs (*16*–*18*). Building on this foundation, we considered a comprehensive suite of plausible ion properties previously associated with SIEs in other systems (section S2.5 and table S15). We performed an array of pairwise Pearson correlation coefficient (*r*) tests with a *t* test significance threshold of α = 0.05 to identify each ion property that linearly correlates with $∆E_{a}^{m-s}$. The significant results were then validated using principal component analysis as detailed in the Supplementary Materials. The results of this statistical analysis are presented in Fig. 8.

The Pearson correlation coefficient test removed most ion parameters from consideration as only four ion properties were significantly correlated with $∆E_{a}^{m-s}$ (Fig. 8A). Notably, $E_{a}^{s}$ does not strongly correlate with $∆E_{a}^{m-s}$, suggesting that ion interactions with the membrane are the source of the observed trends in $∆E_{a}^{m-s}$. The ion softness, σ, stands out as the most strongly correlated property. The validation steps taken to analyze the modes of variance among the significant properties reveals that σ is particularly informative. In addition to the strong linear correlation with $∆E_{a}^{m-s}$, σ shares similar modes of variance as both the experimental transport energetics and the simulated ion interactions (fig. S37). Plotting each of these correlations explicitly (Fig. 8, B and C) reveals that σ is even more closely aligned with the simulated energy barrier than with the experimental one. The colinearity and shared variance among these three properties reinforces the connection between the experimental and simulated energy barriers and identifies a clear ion property to analyze: σ, the softness parameters of the counterions.

The ion softness quantifies the extent to which the ionization of a species is compensated by ion-solvent interactions (*41*, *85*, *86*). Conceptually, σ reflects the malleability of the ion hydration shell (*18*, *41*) (Fig. 8D). Ions with excess stability derived from solvation do not bind waters very strongly and are considered “soft” (*41*). Soft ions typically have large bare radii (e.g., I^−^ or NMe_4_^+^). Conversely, when solvation does not compensate the energy of ion formation, the energy deficit causes an ion to bind its hydrating waters strongly and become “hard” (*41*). Hard ions typically have small bare radii (e.g., F^−^ or Li^+^). Although σ is related to the ion polarizability (α), which describes the malleability of the ion electron shell, the two properties are distinct.

The concept of ion softness is rooted in the hard/soft acid/base (HSAB) theory, developed by Ahrland and Pearson in the 1960s (*25*, *41*, *85*, *87*–*89*). However, these collaborators’ interpretations ultimately diverged: Pearson focused on the solvent-independent molecular hardness (*90*), rejecting solvent-based definitions of softness (*25*), whereas Ahrland advocated for a solvent-based definition, specifically publishing on the ionic softness (*41*, *85*). Our work follows the Ahrland definition, using σ values reported by Marcus as the predictor for ion interaction strength. Although either version of HSAB is sparingly applied in recent polyelectrolyte literature (*16*), HSAB remains distinct from other more modern SIE theories due to the ability to quantitatively predict ion interaction extents, an advantage enabled by Marcus’s extensive characterization of ion softness (*91*, *92*). Therefore, Ahrland’s HSAB framework is based on the principle of like/like interactions and serves as a less empirical, thermodynamically grounded precursor for the more widely adopted LMWA (*16*).

According to Ahrland (*41*), ion-ion interactions are governed by σ because the malleability of the hydration shells determines whether collaborative hydration between ions is feasible (Fig. 8D). Soft ions permit hydrating waters greater freedom of movement, allowing the water molecules to adopt intermediate positions that hydrate two ions simultaneously. In such cases, the entropic penalty for immobilizing the water molecule is paid just once, whereas the energetic benefit of hydration is realized twice. As a result, soft ions tend to form solvent-mediated ion pairs with other soft ions. Meanwhile, hard ions rigidly anchor their hydrating waters, preventing such compromise and inhibiting shared hydration between ions. These strongly bound water molecules provide a strong entropic incentive for dehydration, leading to the formation of contact ion pairs between two hard ions. When a hard ion interacts with a soft ion, neither hydration mechanism dominates, and the resulting interactions are relatively weak. These guidelines can help analyze the expected behavior of the IEM system under study.

The steric bulk of polymer backbones helps to intensify the soft behavior of ions (e.g., for RSO_3_^−^, ${\sigma |}_{R=\text{polymer}}>{\sigma |}_{R=\text{methyl}}$). As in the hydration analysis, we can benchmark our expectations using analogous mobile ions. NMe_4_^+^ is a soft cation (σ = 0.51), and the AEM fixed charge group, RNMe_3_^+^, is expected to be softer, encouraging solvent-mediated ion pairing with soft anions and suppressing contact ion pairing. This is consistent with the phenomenological and correlative analyses presented above. The analogous SO_4_^2−^ is moderately hard (σ = −0.08), but because of enhanced delocalization, we expect RSO_3_^−^ to be softer. We therefore presume that RSO_3_^−^ is essentially neutral (σ ∼ 0) or slightly soft, which would make it susceptible to both solvent-mediated ion pairs or contact ion pairs provided that the countercation has a strong driving force for pairing (∣σ∣ ≫ 0). Once again, this is consistent with the data, where NMe_4_^+^ forms a solvent-mediated pair with RSO_3_^−^, and only the two hardest cations under study (Li^+^ and Ca^2+^) show appreciable contact ion pairing (Fig. 5F). σ is one of only three ion properties in table S15 where the trend between Mg^2+^ and Ca^2+^ opposes the trend between Li^+^ and Na^+^, which makes HSAB uniquely equipped to rationalize the contact ion pairing detected via Raman spectroscopy. This agreement with our phenomenological investigation leads us to conclude that not only does σ correlate with $∆E_{a}^{m-s}$ but also the hydration shell interaction mechanism proposed by Ahrland (*41*) is the primary driver of ion-specific energy barriers to transport in these representative IEMs.

## DISCUSSION

Installing ion-specific interactions in IEMs could facilitate the separation of ions with similar charge and valence. To leverage IEMs effectively for this application, a foundational understanding of SIEs is essential. In this study, we explored this phenomenon by investigating highly swollen IEMs, typical of those used for desalination. Our results demonstrated that solvent-mediated interactions between ions and fixed charge groups are the primary drivers of SIEs in these IEMs. Although contact ion pairs were detected for densely charged monovalent ions (e.g., Li^+^) and multivalent ions, most monovalent counterions interacted with the polymeric fixed charge groups via cooperative hydration of water molecules. This interaction contributed to an enthalpic barrier and a compensating entropic incentive for ion transport, forming a linear free energy relationship. Critically, the strength of solvent-mediated counterion/fixed charge group interactions measured in simulated IEMs strongly correlated with the increased enthalpic transport barriers measured experimentally, which, in turn, were linked to tabulated ion softness parameters described by Ahrland in HSAB theory. As HSAB theory captures both the mechanism and magnitude of these ion transport phenomena, it provides a robust framework to explain the observed SIEs.

Our findings raise important questions about the broader applicability of HSAB-governed SIEs in IEMs. The cooperative hydration mechanism depends on the presence of weakly interacting waters that can then be shared between mobile ions and fixed charge groups. However, it remains unclear how these effects manifest in IEMs with lower water contents, where more water molecules are strongly oriented and where a lower dielectric constant might promote additional contact ion pairing. Even in higher water content IEMs, such as those studied here, replacing the acrylic polymer backbone (which strongly orients approximately five waters per fixed charge group) with a more hydrophobic polymer backbone (e.g., styrenic or perfluoroalkyl) could substantially alter water interactions. In addition, although the idealized experimental conditions used in this study provided fundamental insights, future studies must verify whether trends from HSAB hold in salt-equilibrated, mixed ion membrane systems that better represent real-world separation conditions. Competitive interaction sites and ion-correlated motion could affect the mobility of counterions in IEMs, leading to deviations from the ideal energy differences measured in this study. Despite these uncertainties, our comprehensive characterization of these model IEMs establishes a foundation for future research aimed at understanding SIEs in IEMs under more complex and application-relevant conditions.

Further exploration of SIEs for tuning ion interactions and transport rates in IEMs will enable targeted design strategies to enhance selectivity between like-charged species. This goal underscores the importance of linking experimental findings to a predictive framework like Ahrland’s HSAB model. According to HSAB theory, the softness of the fixed charge groups is the key determinant of ion-specific transport in IEMs. Thus, varying the fixed charge group softness presents a promising strategy for controlling specific ion interactions and achieving like-charge ion separations. Softer fixed charge groups, characterized by greater bulk and charge delocalization, are predicted to inhibit the transport of soft counterions via stronger solvent-mediated interactions while promoting the transport of hard counterions. Conversely, harder fixed charge groups, which are smaller and less charge delocalized, are expected to inhibit the transport of hard counterions via contact ion pairing while promoting the transport of soft counterions. By precisely designing the fixed charge group hydration shell to fine-tune ion selectivity between like-charged monovalent ions, IEMs could provide a highly effective solution for challenging like-charged ion separations.

## MATERIALS AND METHODS

The materials and methodologies used in this study are comprehensively detailed in the Supplementary Materials. Section S1 outlines the synthesis and experimental characterization of the IEMs used in this study. Section S2 describes the procedures for sourcing salt conductance data and ion properties from the literature, isolating single-ion limiting conductances, analyzing transport data using TST, probing vibrational spectroscopy using DFT, and performing statistical analyses of the datasets. Section S3 discusses the methods and parameters used to construct atomistic models, perform MD simulations, and extract equilibrium properties of simulated membranes.

The IEMs used in this work were synthesized from [2-(methacryloyloxy)ethyl]trimethylammonium (MOETMA), 3-sulfopropyl methacrylate (SPM), and glycerol dimethacrylate (GDMA), shown in Fig. 1. MOETMA and GDMA were copolymerized to form AEMs in the chloride form, whereas SPM and GDMA were copolymerized to form CEMs in the potassium form. For brevity, we will refer to ions using elemental symbols. For the cations studied, Li^+^ is lithium, Na^+^ is sodium, K^+^ is potassium, Cs^+^ is cesium, NH_4_^+^ is ammonium, NMe_4_^+^ is tetramethylammonium, Mg^2+^ is magnesium, and Ca^2+^ is calcium. For the anions studied, F^−^ is fluoride, Cl^−^ is chloride, Br^−^ is bromide, I^−^ is iodide, NO_2_^−^ is nitrite, NO_3_^−^ is nitrate, and SO_4_^2−^ is sulfate. In addition, for the fixed charge groups: RNMe_3_^+^ is the trimethylammonium fixed charged group of the MOETMA repeat units in the AEMs and RSO_3_^−^ is the sulfonate fixed charge group of the SPM repeat units in the CEMs.

The MD simulations and some statistical analyses excluded the following ions: NO_2_^−^, SO_4_^2−^, Mg^2+^, and Ca^2+^. The divalent ions seem to follow different trends than the monovalent ions (as discussed in the main text), and with only three divalent ions under study, these differences would confound other analyses. Meanwhile, extremely limited availability of literature data for aqueous NO_2_^−^ prevented analysis analogous to that for the other monovalent ions. These four ions are still included as experimental data points where appropriate.

## Supplementary Materials

### This PDF file includes:

Sections S1 to S3 Figs. S1 to S37 Tables S1 to S15 References
