# Supplementary material for: Specific ion effects on ion transport in charged polymer membranes
Source: Sci Adv. 2026 Mar 11;12(11):eadx1214. doi: 10.1126/sciadv.adx1214 (PMC12978233; doi:10.1126/sciadv.adx1214)
Supplement: Supplementary file 1 — Sections S1 to S3 Figs. S1 to S37 Tables S1 to S15 References [file sciadv.adx1214_sm.pdf]

Science Advances
AAAS

Supplementary Materials for

Specific ion effects on ion transport in charged polymer membranes

David Kitto *et al.*

Corresponding author: Jovan Kamcev, jkamcev@umich.edu

Sci. Adv. **12**, eadx1214 (2026)
DOI: 10.1126/sciadv.adx1214

This PDF file includes:

Sections S1 to S3
Figs. S1 to S37
Tables S1 to S15
References

S1 Experimental Methods

S1.1 Reagents

1-propanol (certified), sodium chloride (NaCl, ≥99%), and cesium chloride (CsCl, ≥99.5%) were purchased from Fisher Bioreagents (Fair Lawn, NJ). 3-Sulfopropyl methacrylate potassium salt (SPM, 98%), [2-(Methacryloyloxy)ethyl] trimethylammonium chloride solution (MOETMA, aqueous, 75%), sodium bromide (NaBr, ≥99%), sodium iodide (NaI, ≥99.5%), sodium nitrate ($NaNO_3$, ≥99.0%), ammonium nitrate (NH_4NO_3, ≥98%), and potassium chloride (KCl, 99.0-100.5%) were purchased from Sigma-Aldrich (St. Louis, MO). Sodium fluoride (NaF, 99%) was purchased from Alfa Aesar (Heysham, United Kingdom). Sodium nitrite ($NaNO_2$, ≥97%), sodium sulfate (Na_2SO_4, ≥99%), lithium chloride (LiCl, ≥98.5%), n-heptane (99%), trace metal sulfuric acid (H_2SO_4, 93–98% with <1 ppb metals), trace metal nitric acid (HNO_3, 67–70% with <1 ppb metals), and hydrogen peroxide (H_2O_2, aqueous, 29.0–32.0%) were purchased from Fisher Chemical (Fair Lawn, NJ). Glycerol dimethacrylate (GDMA, mixed isomers, >90.0%) was purchased from TCI Chemicals (Portland, OR). 2,2'-Azobis(2-methylpropionamidine) dihydrochloride (V-50, 98%) was purchased from Thermo Scientific (Ward Hill, MA). Tetramethylammonium chloride (NMe_4Cl, >99.0%) was purchased from Supelco (Bellafonte, PA). Magnesium chloride solution ($MgCl_2$, aqueous, 1 M) and calcium chloride solution ($CaCl_2$, aqueous, 1 M) were purchased from Honeywell-Fluka (Charlotte, NC). Type I Deionized (DI) water (18.2 MΩ•cm and less than 10 ppb TOC) was produced using a Mili-Q IQ 7000 Water Purification System (Burlington, MA). All reagents were used as received, unless otherwise specified.

S1.2 Membrane Synthesis

Cross-linked ion-exchange membranes (IEMs) were synthesized in a similar manner to our previously reported procedures (*81*, *94*). We used MOETMA as the charge monomer for the anion-exchange membrane (AEM) and SPM as the charged monomer for the cation-exchange membrane (CEM). GDMA served as an uncharged cross-linker for both the AEM and CEM (Fig. 1). Briefly, the charged monomer, cross-linker, and solvent were mixed with magnetic stirring in a 20 mL glass scintillation vial in the amounts listed in Table S1. The solution became homogeneous after approximately 30 minutes of stirring at room temperature, at which point the thermal initiator V-50 was added. The initiator was dissolved over another 30 minutes, producing a clear, homogeneous pre-polymer solution.

The pre-polymer solution was cast between two glass plates that had been pretreated using a KOH/IPA base bath for three hours. Metal spacers were also placed between the plates to enforce a membrane thickness of approximately 300 μm in the relaxed state following polymerization. The cast solution and plates were then polymerized in a Thermo Fisher Scientific (Waltham, MA) HERAtherm 60 L forced convection oven for 40 min at 85 ºC. Afterwards, the glass plates were separated, and the membrane films were placed in DI water. This water was replaced periodically (at least three times) over 24 hours to remove any unreacted monomer and organic solvents. Membranes were then cut into rectangular coupons, 20–30 mm long and 2–8 mm wide.

The densities of the pre-polymer solutions (ρ_{pp}) were measured using five aliquots of solution sampled at room temperature (21 ± 1 °C). 0.5 mL of solution ($V_{aliquot}$) was withdrawn from a well-mixed pre-polymer solution using a 1 mL Eppendorf Xplorer micropipette (Enfield, CT). The liquid was then dispensed onto a Sartorius MCA-125P-2S00 semi-microanalytical mass balance (Gottingen, Germany) where its mass ($m_{aliquot}$) was recorded. ρ_{pp} was calculated as:

$$\rho_{pp} = \frac{m_{aliquot}}{V_{aliquot}} \quad \text{S1}$$

S1.3 Counter-Ion Conversion

The membrane synthesis procedure produces AEMs in the chloride (Cl^-) form and CEMs in the potassium (K^+) form, based on MOETMA and SPM salts that are commercially available. An aqueous salt solution was used to exchange these counter-ions to produce IEMs in other counter-ion forms. Each conversion involved soaking the samples in 1 M salt solution for three days, with the solution being replaced twice per day. To remove any additional sorbed salts, the converted membranes were then re-equilibrated with DI water over an additional day with periodic (at least three) water changes. AEMs were converted to the fluoride (F^-) form using NaF, the bromide (Br^-) form using NaBr, the iodide (I^-) form using NaI, the nitrite (NO_2^-) form using $NaNO_2$, the nitrate (NO_3^-) form using $NaNO_3$, and the sulfate (SO_4^{2-}) form using Na_2SO_4. CEMs were converted to the lithium (Li^+) form using LiCl, the sodium (Na^+) form using NaCl, the cesium (Cs^+) form using CsCl, the ammonium (NH_4^+) form using NH_4NO_3, the tetramethylammonium (NMe_4^+) form using NMe_4Cl, the magnesium (Mg^{2+}) form using $MgCl_2$, and the calcium (Ca^{2+}) form using $CaCl_2$. For consistency, membranes to be used in the Cl^- and K^+ forms were also treated with NaCl and KCl, respectively, via the same process. Measuring the charge content (Section S1.5) for each membrane counter-ion form confirmed that the counter-ion conversions were fully complete.

S1.4 Membrane Water Contents and Membrane Densities

The water content of membranes equilibrated with DI water was measured at 10 ºC, 60 ºC, and at room temperature (21 ± 1 ºC). Hydrated samples for these experiments weighed at least 0.1 g to ensure experimental accuracy. Prior to measurements, hydrated membrane coupons were equilibrated at the measurement temperature for 24 hours. To determine the wet mass (m_{wet}),

samples were removed from the solution, quickly blotted with a KimWipe so that surface water was removed with minimal loss of internal water, then weighed with a Sartorius MCA-125P-2S00 mass balance (Goetingen, Germany). The samples were out of the temperature-controlled water for approximately 20 seconds; after measurement, the samples were returned to a reservoir of water held at the measurement temperature for 1 minute. Coupons were then removed and photographed before returning to the temperature-controlled water. These images were analyzed via Image-J to determine the hydrated surface area (A) of each sample. Finally, the re-equilibrated samples were removed from water and their hydrated thickness (ℓ) was measured at five different locations using a Mitutoyo IP65 micrometer (Kawasaki, Japan).

Samples characterized for their hydrated mass and size were then placed in a Lab-Line vacuum oven (Thermo Fisher Scientific, Waltham, MA) to dry. IEMs in most counter-ion forms were dried under vacuum at an elevated temperature of 85 ºC; however, to avoid degradation of the membrane, membranes in the F^-, NO_2^-, and SO_4^{2-} forms were dried under vacuum at room temperature. Samples were periodically weighed until they reached a stable mass, which was assigned as the sample dry mass, m_{dry}. The sample dry mass stabilized after approximately 1.5 weeks for membranes in monovalent counter-ion forms dried at elevated temperatures, but approximately 3 weeks for the room temperature dried membranes and the divalent counter-ion form membranes dried at elevated temperatures. Assuming water in the membrane maintains a similar density to its bulk value of ($\rho_w = 1$ g/mL), these results were used to calculate the water uptake (WU, g[water]/g[dry polymer]) and water volume fraction (ϕ_w, L[sorbed water]/L[hydrated membrane]) of the IEMs:

$$WU = \frac{m_{wet} - m_{dry}}{m_{dry}} \qquad \text{S2}$$

$$\phi_w = \frac{(m_{wet} - m_{dry})/\rho_w}{A\ell} \qquad \text{S3}$$

Dried water content samples were also used to determine the hydrated membrane and dry polymer densities (ρ_m (g[hydrated membrane]/mL[hydrated membrane]) and ρ_p (g[dry polymer]/mL[dry polymer]), respectively). After samples reached their dry mass, they were removed from the vacuum oven a final time and immersed in n-heptane. The measured weight of the immersed sample was recorded as m_{hept}. Samples were then removed from n-heptane and allowed to dry completely. By knowing the density of this auxiliary non-solvent (ρ_{hept}), Archimedes' principle was used to determine the volume of the dried sample, which often did not retain the simple flat geometry of its hydrated state. With this information, the hydrated membrane and dry polymer densities were calculated according to:

$$\rho_m = \frac{m_{wet}}{A\ell} \qquad \text{S4}$$

$$\rho_p = \frac{m_{dry}}{(m_{dry} - m_{hept})/\rho_{hept}} \qquad \text{S5}$$

S1.5 Membrane Charge Contents

To determine the charge content of the IEMs in different counter-ion forms, dried samples from the water content experiments were immersed in aqueous salt solutions to remove the counter-ions initially present in the sample (n_g). The removal process varied across the counter-ion forms of the IEMs, as outlined below. After equilibration with the salt solutions, the concentration of desired ions in the desorption solution was determined via elemental analysis. Metal cation concentrations were determined using an Agilent 4210 Microwave Plasma Atomic Emission Spectrometer (MP-AES, Agilent, Santa Clara, CA) using an aqueous nitric acid matrix. Organic cation concentrations were determined using a Dionex IonPac cation-exchange chromatograph (Thermo Fisher Scientific, Waltham, MA) with aqueous methanesulfonic acid as

the mobile phase. All anion concentrations were determined using a Dionex Integrion anion-exchange chromatograph (Thermo Fisher Scientific, Waltham, MA) with aqueous potassium hydroxide as the mobile phase. Knowing the ion concentration and volume of the desorption solution allowed calculation of n_g for each sample.

Chromatography samples were prepared via a desorption technique. To remove F^-, Cl^-, Br^-, NO_2^-, and NO_3^- from dry AEMs, samples were placed in a 100 mL 0.03 m NaI desorption solution for at least 24 hours. I^- is generally preferred by AEMs, so all the counter-ions initially present in the membrane were eluted into the supernatant solution. SO_4^{2-} ions are slightly more preferred than the other ions, so a 4-stage desorption was performed instead. After the initial desorption, the solution was collected, and the membrane was exposed to 40 mL of additional 0.03 m NaI. This solution was then removed, and the process was repeated twice more. After four stages, the collected solutions were combined and fixed to a volume of 250 mL. To remove I^- from AEMs, a 4-stage desorption was also performed but used 1 M Na_2SO_4 as the desorption solution. To remove NH_4^+ and NMe_4^+ from dry CEMs, a 4-stage desorption was performed using a 0.1 m $MgCl_2$ desorption solution.

MP-AES samples were prepared via a digestion technique. Digestion was utilized because the ashing technique that we previously utilized (*81*, *94*) was inadequate for Cs^+, Mg^{2+}, and Ca^{2+} samples. To digest dry membranes containing Li^+, K^+, Cs^+, Mg^{2+}, and Ca^{2+}, 4 mL piranha solution (a 3:1 mixture of H_2SO_4 and H_2O_2) was prepared *in situ* in a 20 mL scintillation vial containing dried membranes from water content experiments. Piranha solutions were heated to 50 ºC and stirred for three days. After 24 hours, the membranes were reduced to small pieces, and after 48 hours, the solutions appeared homogeneous and exhibited no signs of insoluble membrane pieces. These piranha solutions were diluted using the nitric acid matrix for analysis. To mitigate Na^+

leaching from borosilicate scintillation vials, 8 mL screw-cap pyrex test tubes were used for dry membranes in the Na^+ form. Na^+ samples were otherwise treated identically to the other digestion samples.

Once samples were processed to obtain n_g, by electroneutrality, the number of fixed charge groups in the membrane was calculated. For divalent ions, this process required accounting for the counter-ion valence (z_g) in the electroneutrality charge balance. Various measures of membrane charge content, including the ion-exchange capacity (IEC, meq[fixed charge]/g[dry polymer]), the fixed charge density ($C_A^{m,t}$, mol[fixed charge]/L[hydrated membrane]), the fixed charge concentration ($C_A^{m,w}$, mol[fixed charge]/L[sorbed water]), the membrane hydration number (λ, mol[sorbed water]/mol[fixed charge]), and the maximum charge density (C_A^{max}, mol[fixed charge]/L[dry polymer]) were calculated according to:

$$IEC = \frac{|z_g| n_g}{m_{dry}} \qquad \text{S6}$$

$$C_A^{m,t} = \frac{|z_g| n_g}{A\ell} \qquad \text{S7}$$

$$C_A^{m,w} = \frac{|z_g| n_g}{(m_{wet} - m_{dry})/\rho_w} = \frac{1}{\lambda \bar{V}_w} \qquad \text{S8}$$

$$C_A^{max} = \frac{|z_g| n_g}{(m_{dry} - m_{hept})/\rho_{hept}} \qquad \text{S9}$$

Here, $\bar{V}_w$ represents the partial molar volume of water in the membrane, which is assumed to be the bulk value of 18 mL/mol.

To ensure reliable conversion into divalent counter-ion forms, we assessed whether stray Na^+ or Cl^- ions disrupted the charge balance between fixed charge groups and divalent counter-ions. In the randomized structure of an IEM, an isolated charge group may result in a divalent

counter-ion electrically balancing one fixed charge and one mobile ion, even when equilibrated with DI water. To investigate whether this phenomenon occurred in the IEMs studied here, we digested SO_4^{2-} AEMs to confirm that no Na^+ was detected. Similarly, we digested Mg^{2+} and Ca^{2+} CEMs to confirm that no Cl^- was detected. For all three divalent counter-ion forms studied, no residual Na^+ or Cl^- ions were detected, suggesting that each counter-ion is balanced by two fixed charges.

S1.6 Polymer Gel Fractions

The gel fractions (GF) of the AEM and CEM were determined by modifying the polymerization and water uptake protocols. Before polymerization, the amount of pre-polymer solution deposited onto the casting plates was weighed. By accounting for the mass of polymerizable monomers and the solvent, the maximum mass of the resulting membrane, $m_{initial}$, was calculated. This solution was then polymerized as described in Section S1.2. The membrane samples were stored in DI water to remove unreacted components but were never cut or exposed to salt solution. Instead, the entire membrane was dried per Section S1.4. Here, the mass of the dried polymer represents the mass of the monomer and cross-linker successfully incorporated into the polymer network, m_{gel}. GF was then calculated as:

$$GF = \frac{m_{gel}}{m_{initial}} \tag{S10}$$

S1.7 Ionic Conductivities

Ionic conductivity measurements were performed according to our recently published methodology (*52*), which we briefly summarize below. We refer the reader to this publication for the full details. The ionic conductivity of each sample in the in-plane direction was determined via electrochemical impedance spectroscopy (EIS). We employed a BioLogic controlled environment sample holder (CESH) (Knoxville, TN) with four parallel gold-plated electrodes and a BioLogic

SP-300 potentiostat for the data collection. Temperature regulation was performed using a Binder KB53 Refrigerated Incubator (Tuttlingen, Germany). Rectangular samples (at least 2 mm wide, at least 2 cm long, and approximately 300 µm thick) were equilibrated with DI water prior to the experiment. Reported conductivities represent the average of results for four independent samples.

The thickness of membrane samples was recorded at three separate locations using a Mitutoyo IP65 micrometer (Kawasaki, Japan). The width of samples was recorded at three separate locations using Fowler Ultra-Cal V calipers (Valencia, CA). Hydrated samples were placed perpendicular to the four electrodes, allowing impedance measurements at path lengths of 1.0 cm, 1.3 cm, and 1.6 cm. Samples were pressed into the electrodes with the top stage to ensure good contact, and the electrode assembly was then sealed into the CESH. Inside the sealed chamber, we also included moist filter paper and standing water to establish a humid environment, which limits membrane dehydration during experiments. The entire CESH was placed in the incubator and equilibrated at the desired temperature for approximately 2.5 hours. Frequency sweeps probed the impedance response to an oscillating 100 mV potential from 3 MHz to 100 Hz every five minutes during the experiment.

The conductivity was extracted from impedance scans using a modified Randles circuit (Figure S1) to isolate the resistance of the membrane film. The membrane resistance was normalized to the sample geometry as:

$$\kappa = \frac{\ell}{R_m A_x} \qquad \text{S11}$$

Here, κ (mS/cm) is the membrane ionic conductivity, ℓ is the electrode separation length, R_m is the membrane resistance extracted from the equivalent circuit, and A_x is the cross-sectional area of the membrane coupon (the width multiplied by the thickness). To automate the temperature variation (between 10 °C and 60 °C), we validated the results of the equivalent circuit fit at the

most difficult temperature to fit. We compared the membrane conductivity extracted from the equivalent circuit fit with the membrane conductivity extracted from the varying length method, described in our previous publication (*52*). The varying length method unambiguously isolates the membrane conductivity from resistance measurements at all three lengths of electrode separation using the following equation:

$$\kappa = \frac{d\ell}{d(R_c A_x)} \qquad \text{S12}$$

Here, R_c is the total cell resistance measured at the right-most x-intercept of a Nyquist plot. If the two methods produced conductivity measurements in agreement with each other, we proceeded to use the equivalent circuit at lower temperatures with confidence. The reduced membrane conductivity at lower temperatures ensured that lower frequencies made up the membrane impedance response (semicircle of the Nyquist plot) rather than the semi-infinite diffusion response (tail of the Nyquist plot), producing a more reliable equivalent circuit fitting. If the two methods did not agree, we attributed the discrepancy to inadequate data for the equivalent circuit fit and repeated the comparison at lower temperatures (Table S8). Once the methods agreed, the equivalent circuit fit was used to extract membrane resistance at all lower temperatures.

S1.8 Raman Spectroscopy

Raman spectra for both dried and hydrated membranes were collected using a 532 nm, 100 mW laser and a RenCam CCD detector within a Renishaw InVia Raman microscope (West Dundee, IL). Spectra were collected in static scan mode from 260 to 2000 cm^{-1} with 1800 lines/mm grating and a 65 µm slit. The spectral resolution was 1.53 cm^{-1}, and 32 replicate scans were performed per sample. All samples were held in sealed borosilicate glass vials during collection. Hydrates samples were immersed in DI water within the glass vial. Dry samples were dried in a vacuum oven as reported for water content experiments (Section S1.4) and then sealed in the glass

vial with an argon headspace. Due to the varied path lengths inherent to confocal spectroscopy, quantitative comparisons of spectral intensities were not performed between different samples.

S1.9 Fourier Transform Infrared Spectroscopy (FTIR)

FTIR spectra for both dried and hydrated membranes were collected with an attenuated total reflectance (ATR) probe using a Thermo Fisher Nicolet Smart iTX iS20 (Billerica, MA). Hydrated membranes in each counter-ion form were equilibrated with DI water, blotted dry on one side, and then clamped down with the blotted side facing the ATR diamond. For spectra of the dried membranes, samples were dried under vacuum as reported for water content experiments (Section S1.4) and samples were clamped onto the ATR diamond immediately after removal from the drying oven. For both dried and hydrated samples, the spectra were recorded over 64 scans at a 0.48 cm^{-1} resolution, lasting approximately 1 minute. Within this minute, water peaks did not significantly change in the sample spectrum, suggesting that water gain/loss was minimal.

S1.10 Differential Scanning Calorimetry (DSC)

Melting behavior of water in membranes was characterized via DSC using a TA Instruments DSC250 (New Castle, DE) employing a nitrogen gas purge. Approximately 5 mg of hydrated membrane was laid flat in a TZero hermetic aluminum pan after being quickly blotted with a KimWipe to remove surface water. The hermetically sealed pans were quenched to −80 ºC at 20 ºC/min, annealed for 10 min, and then heated back to 40 ºC at 10 ºC/min. The enthalpy of melting was determined as the average integration of the water melting peak across >5 samples. The integration limits were placed at the onset of non-zero derivatives of the thermograms. Enthalpies of melting, referenced to the mass of the sample, were re-normalized to the mass of water in the sample (ΔH_m^m) using the water content results.

The melting enthalpy of water in membranes is a helpful way to understand water interactions with its surroundings in the membrane (*36*, *79–81*). Water is categorized into two distinct states by normalizing the membrane melting enthalpy by the bulk melting enthalpy of water, $\Delta H_m^s = 333\ \mathrm{J/g}$. The difference between the two enthalpies is attributed to water that does not freeze, either due to domain-size effects or strong interactions with its surroundings. We calculated the freezable (λ_f) and non-freezable (λ_{nf}) hydration number of the IEMs using the following equations:

$$\lambda_f = \frac{\Delta H_m^m}{\Delta H_m^s}\lambda \qquad \text{S13}$$

$$\lambda_{nf} = \left(1 - \frac{\Delta H_m^m}{\Delta H_m^s}\right)\lambda \qquad \text{S14}$$

These definitions identify just two populations of water, $\lambda = \lambda_f + \lambda_{nf}$. However, ion-water interactions are typically grouped into three states of water (*53*): strongly bound, weakly bound, and free. To integrate these frameworks, we distinguish that strongly bound waters, forming the dynamic ion hydration shell, are thought to be non-freezable. Meanwhile, weakly bound waters do not interact with the ion strongly enough to prevent freezing; instead, these waters are presumed to freeze at temperatures lower than 0 ºC. To analyze this data quantitatively, we group weakly bound and free waters into the freezable designation.

S2 Reference Data and Statistical Methods

S2.1 Interpreting Vibrational Spectroscopy

S2.1.1 Gaussian Deconvolution of Spectra

To quantitatively interpret the vibrational spectroscopy results, signals associated with the IEM fixed charge groups were analyzed via Gaussian deconvolution. Before deconvolution, Raman spectra were pre-processed to reduce the influences of fluorescence and sensor noise on

the Gaussian curve fitting. First, spectra were baseline-corrected using an asymmetric least squares fitting algorithm (*95*). Then, further processing was performed using spectrapepper (*96*) (v0.1.10). Sampling noise was filtered using the function *lowpass* with a Frequency Cutoff of 250 Hz, a Sample Rate of 60 Hz, a Sinusoid Approximation Order of 2, and a Nyquist Frequency of 22.5 Hz. Spectra were intensity-normalized using the function *normtopeak,* such that the signals associated with the fixed charge group (see Section 0) integrate to unity.

Gaussian peaks were then fit to the pre-processed Raman spectra using the *fit* function in MATLAB (R2024a). Fitting parameter bounds of 0 and 10^8 ensured peak areas remained positive but were otherwise essentially unbounded; the maximum observed peak parameter was below 10^4. To determine the number of components for each spectrum, the quality of the fit was tracked as the number of Gaussian components was increased (Figure S30). The root mean square errors of fits with increasing numbers of Gaussian peaks were compared to each other, revealing the threshold where additional components would not improve the quality of the fit. However, sometimes the added components were at the same wavenumber as other peaks. We sought non-redundant components that significantly reduced the error of the fit. For the spectra of each dried and hydrated AEM, three distinct peaks produced the best fits. For CEMs, the results depend on the counter-ion form. K^+, Cs^+, NH_4^+, and NMe_4^+ form CEMs produced spectra fit with just one component in both hydrated and dried states. For these CEMs, trialed second components produced minimal changes in the fitting error (Figure S30) and were placed at the same wavelength as the first component. Li^+ and Na^+ form CEMs, in both hydrated and dried states, required two distinct components, as did hydrated Mg^{2+} and Ca^{2+} form CEMs. Dried Mg^{2+} and Ca^{2+} form CEMs required three distinct components. The assignments of these components are discussed in the following section.

When multiple signals overlap with one another, such as the Raman spectra analyzed in this work, fitting parameters associated with deconvoluted Gaussian peaks may be correlated with one another. These correlations affect the estimates of statistical confidence produced by the nonlinear fitting algorithm and require a technique such as bootstrapping to estimate the confidence of the fit (Section S2.8). To bootstrap these spectra, we compared four common methods (97)—XY, Non-Parametric, Parametric, and Wild—and found that the methods all essentially agreed with one another (Figure S31). This convergence suggests that the deconvolutions are reliable. To calculate uncertainties displayed in main body Fig. 5, we used the Non-Parametric approach, as it is considered the most appropriate method for analyzing Raman spectra where each data point may have a non-Gaussian error distribution as a function of signal intensity (98).

S2.1.2 Assignment of Vibrational Modes

The ion pairing analysis presented in the main text is contingent on analyzing a signal corresponding to the fixed charge group that is sensitive to contact ion pairing. Vibrational spectroscopy is often utilized to assess interactions within CEMs containing RSO_3^- or other fixed charge groups (*99–101*), so we confidently analyze the symmetric S–O vibrations in the proximity of 1043 cm^{-1}. The symmetric band was chosen for analysis instead of the asymmetric band for two reasons. 1) The symmetric band in our membrane is clearly visible in the spectra of our membranes, while the asymmetric band is partially obscured by the esters in the acrylate backbone. 2) The asymmetric band is bimodal, requiring additional parameters to quantify via Gaussian deconvolution. Previous work has shown that both sulfonate vibrational modes are sensitive to the hydration environment of the fixed charge groups (*100*).

Unfortunately, comparable analyses for AEMs are lacking. Some of the differences are systematic: RSO_3^- exposes the charge-carrying oxygens, whereas the charge-carrying nitrogen of $RNMe_3^+$ is sheltered by the pendant methyl groups. However, we believe some of the reason is derived from the mystery of the C–N stretching mode. Although amines are thoroughly documented in reference materials, there is less data available for ammoniums (*102*). We based our assignment for the quaternary ammonium C–N vibration peak primarily based on the NIST standard spectrum for NMe_4Cl (*103*). Only three prominent peaks are present in this spectrum: $\nu = 930 - 960$, $1400 - 1415$, and $1480 - 1510$ cm^{-1}. By analogue to tertiary amines, both peaks at higher wavenumbers likely correspond to C–H vibrational modes among the methyl groups (*102*). Meanwhile, tertiary amines and primary ammoniums both are reported to exhibit C–N stretches from 900 – 1000 cm^{-1}, suggesting that this final peak of NMe_4Cl corresponds to a C–N stretch (*102*). This location features a prominent peak for both FTIR and Raman spectra of both hydrated and dried AEMs, but the peak is absent in all CEM spectra except for the NMe_4^+ counter-ion form. These trends support identifying the ~ 954 cm^{-1} signal as the C-N stretch of the AEM fixed charge groups.

Three Gaussian peaks were required to accurately fit the C–N stretch in all AEMs (Section S2.1.1). Two small peaks were located at ~930 and ~970 cm^{-1}, surrounding a larger ~954 cm^{-1} signal. Both smaller peaks did not vary significantly between spectra of hydrated and dried membranes, suggesting that they are unrelated to the fixed charge group. Meanwhile, the ~954 cm^{-1} peak was sensitive to the hydration state of the membrane. Therefore, we analyze the 954 cm^{-1} peak as the C-N stretch, while the smaller peaks are included to accomplish an accurate Gaussian deconvolution of the AEM spectra but are not analyzed or interpreted.

For CEMs, when only one peak was found during Gaussian deconvolution, the assignment was unambiguously the symmetric stretching of the S–O bond. When multiple peaks were present, we assigned them to various contact ion pair structures, all still representing the S–O bond. Counter-ions interacting primarily with one oxygen or positioned between all three oxygens of RSO_3^- would yield different signals. Additionally, especially for multivalent counter-ions, the possibility of bidentate pairing where one counter-ion contacts two RSO_3^- groups would give rise to another population. Finally, the interaction strength likely changes for pairs in a fully dried environment compared to a hydrating environment, where water could still be present surrounding the point of ion pairing contact. Due to all these possibilities, we examine the direction of wavenumber shift, but do not make quantitative comparisons between the bond vibrational energies of pairing signals in the wet and dry states. However, except for Li^+, observed pairing peaks in the hydrated spectra do overlap with a dry paired peak location. When multiple peaks were present in the spectra of dry CEMs, for simplicity, the main text refers to the pairing peak with the lowest energy peak and highest integrated area, which should be the most likely conformation to appear in hydrated membranes.

S2.1.3 Predicting Conformations with Density Functional Theory

Experimentally detected Raman shifts of $RNMe_3^+$ stretching (for AEMs) and RSO_3^- stretching (for CEMs) were compared to DFT-predicted Raman shifts for these species with monoatomic, monovalent counter-ions (F^-, Cl^-, Br^-, I^-, Li^+, Na^+, K^+, and Cs^+). To facilitate comparison, the difference in energy between the vibrational energy in the hydrated and dried states were compared using DFT simulations considering both conditions. DFT calculations were performed using the Gaussian software (*104*). Seven replicates were performed for each ion-monomer pair, where replicates differed in their initial atomic configurations. Each replicate used

the same initial randomized geometry for both the dehydrated (gas-phase) and hydrated (implicit solvent) conditions. When systems failed to converge initially, a slight perturbation was introduced to facilitate convergence. Geometry optimizations were performed using Gaussian's relaxed convergence thresholds. Self-consistent field calculations, in both gas phase and implicit solvent, used Gaussian's default convergence criteria. All simulations used the B3LYP functional and Def2TZVP basis set, which have been applied in studies of similar systems to reproduce Raman spectra (*105–108*). The hydrated membrane condition was modeled with the Integral Equation Formalism Polarizable Continuum Model (IEFPCM), which represents a polarizable dielectric medium that responds self-consistently to the electrostatic potential of the atoms (*109*).

To mimic real membrane systems, where ions may interact with multiple nearby fixed charges, each system included two monomers with polymerized backbones and two ions, yielding overall charge neutrality. For an illustration of the final geometries of all ion-monomer pairs and for access to the raw data, readers are directed to Zenodo (*110*); some representative examples are included in Figure S23 and Figure S24. Peak positions for C–N and S–O stretches were determined by calculating an intensity-weighted average or centroid frequency within the observed vibrational ranges (800–1000 cm^{-1} for MOETMA and 950–1150 cm^{-1} for SPM), according to Equation S15.

$$\nu_{centroid} = \frac{\sum_{i \in S} I_i \nu_i}{\sum_{i \in S} I_i} \qquad \text{S15}$$

Here ν_i is the discrete, DFT-computed Raman signal in cm^{-1}, I_i is the corresponding intensity, and S represents the range in which the centroid is computed. The centroid approach used here to compute primary signals provided a representative peak center without assuming a specific linewidth for Gaussian smoothing of discrete signals.

The average wet primary signal for $RNMe_3^+$ with all anions from experiment was 954 cm^{-1} and for DFT it was 921 cm^{-1}. The average wet primary signal for RSO_3^- with all cations from

experiment was 1043 cm^{-1} and from DFT was 1054 cm^{-1}. Although the simulation and experimental values differed, each value served as its own reference for the dried spectra, which induced strong contact ion pairing. The change in vibrational mode energy from the hydrated to the dried state could then be compared directly, even though the average hydrated locations differed.

S2.2 Equivalent Conductances at Infinite Dilution

To source the equivalent conductances of ions at infinite dilution, we employed a similar procedure as that described in our recent publication (*36*). Specifically, we sourced conductance data of salts from literature over the past century (*111–145*). Often the data were expressed as limiting equivalent conductances at infinite dilution (Λ^0, S•cm^2/eq); however, some sources reported enough conductance data to determine Λ^0 but did not actually report values of Λ^0. For these sources, we calculated Λ^0 using the Lattey form (*146*) of Kohlrausch's Law:

$$\Lambda = \Lambda^0 - \frac{A\sqrt{c}}{1 + B\sqrt{c}} \qquad \text{S16}$$

In this equation, Λ is the equivalent conductance at a finite concentration and c is the concentration expressed in molality. A is a parameter grounded in the Debye-Hückel-Onsager framework, while B is a parameter dependent on size (*134*), similar to Bjerrum's extension of Debye-Hückel's theory (*53*). More adjustable parameters, such as those used in the Jones-Dole form of Kohlrausch's Law, could extend the concentration range of experimentation (*53*, *65*, *134*). We selected the Lattey form as a trade-off between requiring results at low salt concentrations and including too many fitting parameters. To extract Λ^0 from experimental data, B was adjusted to maximize the linearity of Λ with the function $\sqrt{c}/(1 + B\sqrt{c})$.

Most sources reporting results for the same salts and temperatures were in reasonable agreement with each other. When the results for a particular salt deviated, it was always for a salt

where a clear majority consensus could be determined by analyzing the results of multiple sources, and the anomalous reference was excluded from analysis. All the assembled conductance data monotonically increased with temperature, as expected. Following Harned and Owen (*147*), salt conductance data from 0 to 100 ºC was fit using a cubic polynomial. The salt limiting equivalent conductances were then decoupled into single-ion limiting equivalent conductances. This step is generally performed by relying on KCl conductances because the transference number of KCl remains relatively constant across temperatures below 50 ºC (*141*, *144*, *148*). By considering discrepant sources, we elected to use a nominal cation transference of 0.495 for KCl at all temperatures, which agrees with all reported values within 1 %. Once both K^+ and Cl^- single-ion limiting equivalent conductances were calculated, the single-ion conductances (λ_i^0, S•cm^2/eq) of other KX and MCl salts were determined by subtracting the contribution of either K^+ or Cl^-:

$$\Lambda_{MX}^0 = \lambda_{M^+}^0 + \lambda_{X^-}^0 \quad \text{S17}$$

NMe_4Cl data was not readily available, so we added an intermediate calculation using the picrate ion, Pic^-. Using NH_4Cl, NH_4Pic, and NMe_4Pic, $\lambda_{NMe_4^+}^0$ could be calculated via this same procedure. To the best of our knowledge, NO_2^- conductance data has only been reported by Klemenc and Hayek (*137*), ranging from 0 to 30 ºC. All other ions spanned the full range of temperatures studied in this manuscript, exceeding the range 10–60 ºC. The range of validity and cubic fits for each λ_i^0 are given in Table S10.

S2.3 Activated Transport Process

Activation energies of transport have historically been extracted from temperature-variate diffusion data by assuming a functional form for the transport rate process (*51*, *149*, *150*). In this formalization, diffusion is treated as a reaction that transports the species from one local environment to another. It is relatively common to report the activation energy of ionic

conductivity for IEMs; however, such values are not often analyzed with a molecular level interpretation. Because we are seeking to understand the molecular source of measured activation energies, some common assumptions inherent in rate process analyses require further consideration. The following subsections introduce nuances related to transport rate processes that become relevant when applying activation energy formalizations to interpret the molecular transport of charged ions.

S2.3.1 Activated Diffusion Equations

Mathematical treatments of activated transport processes have historically been grounded in the diffusion of gas molecules. There are two common forms of the transport rate expression (*50*, *51*, *150*): First, Arrhenius empirically proposed that rate processes would follow Equation S18. Later, Eyring and Polanyi provided a more thermodynamically grounded derivation using transition state theory (TST) for the analogous Equation S19.

$$D_i = D_0 \exp\left(-\frac{E_{a,D}}{RT}\right) \quad \text{S18}$$

$$D_i = \left[\frac{\delta^2 k_B T}{6h} \exp\left(\frac{\Delta S^\ddagger}{R} + 1\right)\right] \exp\left(-\frac{E_{a,u}}{RT}\right) \quad \text{S19}$$

In these equations, D_i is the diffusivity of ion i, D_0 is the unhindered ion diffusivity at infinite temperature (also called the Arrhenius pre-factor), δ is the length of a single ion diffusional jump, R is the ideal gas constant, k_B is the Boltzmann constant, h is the Planck constant, T is the absolute temperature, E_a is the activation energy, and $\Delta S^\ddagger$ is the activation entropy. Because aqueous ion transport is at least partially limited by the rearrangement of solvent molecules, it is common to set δ as the diameter of a water molecule (*51*, *53*), 2.8 Å. The factor of 6 in Equation S19 is related to the dimensionality of diffusion: this factor accounts for the different directions in which a single diffusional jump can be taken. This factor of 6 is (*35*, *53*) or is not (*51*, *54*) included in the Eyring-

Polanyi equation depending on the source; however, it is required to rectify TST with the Einstein-Smoluchowski equation:

$$D_i = \frac{MSD}{2nt} \tag{S20}$$

Here, MSD is the mean square displacement of an ion after diffusing for time t and n is the number of dimensions available for diffusion, i.e., 3. For a single diffusional jump, $MSD = \delta^2$, and the necessity of the $2n = 6$ factor becomes apparent in the comparison of Equations S19 and S20.

Employing Equations S18 and S19 involves fitting experimental values for D_i at various T to either equation. This is typically done by linearizing the equations. Arrhenius coordinates plot ln (D_i) against T^{-1} to easily extract $-E_{a,D}/R$ from the slope and ln (D_0) from the intercept. Eyring coordinates plot ln (D_i/T) against T^{-1} to directly extract $-E_{a,u}/R$ from the slope and $\Delta S^{\ddagger}$ from the intercept. Because of this difference in methodology, the temperature dependence of observed transport rates plays a pivotal role in rate process analyses.

The subscript on E_a differs between these two equations. In this section, $E_{a,t}$ represents the activation energy of proportionality coefficient t, where $t = D$ refers to ion diffusivities (having units of cm^2/s), $t = \lambda$ refers to equivalent ion conductances ($mS \bullet cm^2/eq$), $t = \kappa$ refers to ionic conductivities (mS/cm), and $t = u$ refers to absolute ion mobilities ($cm^2/(s \bullet J)$). As suggested by the distinct subscripts we have used in Equations S18 and S19, experimental values for $E_{a,t}$ extracted from a single data set using Arrhenius and Eyring coordinates will not agree with one another. It is common to claim that $E_{a,t}$ will be identical from the two equations (*34*, *36*, *55*), a claim also made by Eyring (*51*). However, the temperature derivative calculated using each functional form will clearly differ between these two equations, which leads to an experimental difference in $E_{a,t}$. It is also worth noting that Equations S18 and S19 display very different limiting

behavior: at infinite temperature, the Arrhenius equation predicts a diffusivity of $D_i = D_0$, while the Eyring-Polanyi equation predicts $D_i \to \infty$. Additionally, depending on the TST equation used (whether or not the factor of 6 is included), values of $\Delta S^{\ddagger}$ will differ between investigations. Because of all these considerations, one must carefully consider quantitative comparisons of transport rate parameters reported by various sources. Before comparing values, it is essential to analyze not only the experimental methods, but also the mathematical methods used to perform the calculations.

S2.3.2 Proportionality Coefficients for Ion Transport

To understand the apparent inconsistencies between the Arrhenius and Eyring formulations, it is helpful to discuss the mechanism of ion transport using the Nernst-Einstein relationship (*151*, *152*). For ion transport driven by an electrochemical potential gradient, the transport response of an ion to an applied force (f) can be written using the absolute mobility of an ion (u_i) (*53*). The terminal drift velocity (v_d) developed by the ion is written as:

$$v_d = u_i f \qquad \text{S21}$$

such that the absolute mobility has units of $cm/(s \bullet N)$ [=] $cm^2/(s \bullet J)$. When the driving gradient is purely an electric field ($d\Psi/dx$) or concentration gradient (dC_i/dx), the absolute mobility can be transformed via the relationships of Nernst and Einstein into more common proportionality coefficients: the equivalent ionic conductance (λ_i) and the ionic diffusivity (D_i).

$$\lambda_i = |z_i|eF \bullet u_i \text{ for } f = |z_i|e\frac{d\Psi}{dx} \text{ such that } v_d = \frac{\lambda_i}{F}\frac{d\Psi}{dx} \qquad \text{S22}$$

$$D_i = k_B T \bullet u_i \text{ for } f = \frac{k_B T}{C_i}\frac{dC_i}{dx} \text{ such that } v_d = \frac{D_i}{C_i}\frac{dC_i}{dx} \qquad \text{S23}$$

Here, z_i is the ion valence, e is the protonic charge, F is Faraday's constant, Ψ is the electric potential, C_i is the ion concentration, and x is the direction of transport. Combining these equations

using the Nernst-Planck framework gives an expression for the ionic conductivity due to the transport of ion i, κ_i, which is the more common way to express the Nernst-Einstein relationship:

$$\kappa_i = |z_i| C_i \lambda_i = \frac{z_i^2 eF}{k_B T} C_i D_i = \frac{z_i^2 F^2}{RT} C_i D_i \qquad \text{S24}$$

These relationships are valuable for understanding the rate process equations given in the previous sub-section, which are traditionally framed using D_i, for ion transport more generally.

For completeness it is worth noting that an additional proportionality coefficient, called either the electrochemical mobility or the conventional mobility, is also utilized extensively in ion transport studies (*53*). This coefficient functions similarly to λ_i in every way relevant to this discussion. For brevity, we will focus on only one proportionality coefficient for transport driven by an electric potential gradient, i.e., λ_i.

Because this discission relies on the Nernst-Einstein relationship to convert between κ_i, λ_i, D_i, and u_i, it is worthwhile to examine the validity of the Nernst-Einstein relationship in the systems relevant to this manuscript (*151*, *152*). Recent investigations have added support for the validity of the Nernst-Einstein relationship for both DI water equilibrated IEMs and dilute aqueous solutions. Two studies have independently measured single-ion diffusion coefficients at infinite dilution and single-ion limiting conductances at infinite dilution (*112*, *153*), finding reasonable agreement between these independent measurements and the Nernst-Einstein equation. These results support the continued usage of the Nernst-Einstein relationship for dilute salt solutions. In IEMs, it has recently been demonstrated that Nernst-Einstein diffusivities were reasonably accurate so long as co-ion concentrations were minimal (*154*). Since the IEMs in this investigation were equilibrated with DI water and characterized without any co-ions present, the validity of the Nernst-Einstein relationship is reasonable.

S2.3.3 Activation Energies of Generalized Transport Equations

As extensions of the Arrhenius framework, it is common to see Equation S18 analogues employed in electrochemical research to analyze either the ionic conductivity (producing κ_0 and $E_{a,\kappa}$) or the ionic conductance (producing λ_0 and $E_{a,\lambda}$) as activated rate processes. Although less common, the same extension could be drawn for an Arrhenius analysis of the absolute ionic mobility (producing u_0 and $E_{a,u}$). For Arrhenius analyses, these various equations amount to semantic differences. However, when utilizing TST, these different proportionality coefficients instead require a detailed understanding of the differing assumptions in each of these equations. The following discussion attempts to rectify these numerous Arrhenius equations for ion transport with the Eyring-Polanyi equation using the assumption inherent in the existence of a generalized absolute mobility and the relationships of Nernst and Einstein: the inherent force-response of an ion is the same, no matter the applied force. By extension, these theories must assume that an ion will pass through the same transition state as it moves from one location to another, no matter the applied force.

Rate processes deal with the temperature-dependence of transport proportionality coefficients, so it is natural to consider the temperature dependence of the Nernst and Einstein equations. Equation S24 contains two parameters dependent on temperature via a non-activated process. The expressions featuring D_i also contain T^{-1}, revealing that $E_{a,\kappa} \neq E_{a,D}$ within the Nernst-Einstein framework. However, both the λ_i and D_i expressions depend on C_i, which may depend on temperature through system densification and swelling differences. Thus, generally, $E_{a,\kappa} \neq E_{a,\lambda}$ as well. For systems with constant concentration across the temperature range studied, $E_{a,\kappa} = E_{a,\lambda}$, but $E_{a,\kappa} \neq E_{a,D}$. The concentration distinctions result from κ_i behaving more like a flux proportionality coefficient than a single-molecule proportionality coefficient.

Looking only at the proportionality coefficients for transport of single molecules (λ_i, D_i, and u_i), it is helpful to calculate activation energies following Equations S21–S23. The result is:

$$-\frac{d\ln(v_d)}{dT^{-1}} = -\frac{d\ln(u_i)}{dT^{-1}} - \frac{d\ln(f)}{dT^{-1}} = E_{a,u} + E_{a,f} \qquad \text{S25}$$

$$\text{if } f = |z_i|e\frac{d\Psi}{dx} \text{ then } E_{a,f} = 0 \text{ and } E_{a,\lambda} = E_{a,u} \qquad \text{S26}$$

$$\text{if } f = \frac{k_B T}{C_i}\frac{dC_i}{dx} \text{ then } E_{a,f} = T \text{ and } E_{a,D} \neq E_{a,u} \qquad \text{S27}$$

Here, even though the temperature dependence of the applied force is not due to an activated rate process or transition state, for unit consistency we define $E_{a,f}$ as the *artificial* activation energy derived from the applied force. For convenience, the driving force for transport is typically thought to be constant when the macroscopic gradient ($d\Psi/dx$ or dC_i/dx) is held constant. However, because some force terms are included in proportionality coefficients λ_i and D_i, the applied force f can change even when the relevant gradient is held constant. Ions do not change valence as a function of temperature, so it is generally true that $E_{a,\lambda} = E_{a,u}$. However, f depends directly on T for concentration gradient–driven transport. In Equation S27, $E_{a,f}$ represents the same T scaling identified in the previous paragraph. This force scaling increases the observed temperature dependence of diffusion processes, despite the Nernst-Einstein assumption that $E_{a,f}$ should not influence the mechanism of ion motion or the transition state of ion transport.

To interpret transport at the molecular level via TST, it is important to distinguish enthalpic or entropic effects on diffusing species from those related to the applied force. Such a distinction is already present in the Eyring-Polanyi equation (Equation S19): the pre-exponential terms explicitly account for the $k_B T$ relationship between D_i and u_i. Therefore, the activation energy in

TST is $E_{a,u}$, even though the equation is fit to D_i data and not u_i data. Combining Equations S19 and S23 results in a TST equation directly using u_i:

$$u_i = \left[\frac{\delta^2}{6h}\exp\left(\frac{\Delta S^\ddagger}{R}+1\right)\right]\exp\left(-\frac{E_{a,u}}{RT}\right) \quad \text{S28}$$

$E_{a,u}$ in Equations S19 and S28 exclusively refers to the energy required for an ion to execute a single jump through the transition state of motion. In contrast, $E_{a,D}$ in Equation S18 should be greater than $E_{a,u}$ because it accounts for the change in energy available to equalize concentration gradients via thermal motion ($E_{a,f} = T$).

Since $E_{a,\lambda} = E_{a,u}$, we can write directly comparable Arrhenius and Eyring-Polanyi analogues for both ion motion and ion conduction:

$$\lambda_i = \lambda_0 \exp\left(-\frac{E_{a,u}}{RT}\right)\ where\ \lambda_0 = |z_i|eF\frac{\delta^2}{6h}\exp\left(\frac{\Delta S^\ddagger}{R}+1\right) \quad \text{S29}$$

$$u_i = u_0 \exp\left(-\frac{E_{a,u}}{RT}\right)\ where\ u_0 = \frac{\delta^2}{6h}\exp\left(\frac{\Delta S^\ddagger}{R}+1\right) \quad \text{S30}$$

In both equations, $E_{a,u}$ is identical to the value resulting from TST, and the Arrhenius activation energies can be directly related to molecular interactions. The insights offered by this Nernst-Einstein analysis helps resolve the unphysical limiting behavior of the Eyring-Polanyi equation introduced earlier: TST does not predict particles to be infinitely responsive to an applied force at infinite temperature; rather, the Eyring-Polanyi equation describes a finite response (u_0) to an infinite force ($f = (k_BT/C_i)dC_i/dx \to \infty$).

S2.3.4 Activation Entropies of Generalized Transport Equations

So long as all extraneous temperature dependence has been accounted for, the guidance in Section S2.3.3 should sufficiently isolate molecular enthalpic interactions. However, to analyze $\Delta S^\ddagger$ properly, additional terms that do not scale with temperature also become important.

Phenomena that alter the observed transport rate of ions without molecular interactions must be accounted for to properly isolate activation entropies. Even though it is common to report "effective" diffusion coefficients in membrane phases (*149*, *155*), activation entropy analyses require actual diffusion coefficients (or other proportionality coefficients). For data in a dilute aqueous solution, no correction is needed. However, in typical IEMs, obstruction effects account for significant differences between the observed, macroscopic, effective transport rate and the actual speed of molecular ion transport within the membrane (*149*, *155*).

Obstruction effects within membranes refer to the tortuous path that ions must traverse within the polymer/water mixture. Due to the discrepant path lengths traveled by ions within membranes, ions inside membranes will appear to be much slower than in dilute aqueous solution when viewed macroscopically, even if each individual jump covers the same distance and occurs at the same frequency in the two phases. The TST model calculates the molecular components of transport (the jump distance and frequency of jumps) and is agnostic to the path length, so tortuosity effects must be accounted for before using the Eyring-Polanyi equation. Other phenomena that contribute to a shift in the effective transport rate of an ion without altering any single jump must be treated similarly; however, tortuosity effects are typically considered the largest effect of this nature (*155*).

Measured values for κ_i in tortuous media can be transformed via the Nernst-Einstein framework to produce effective conductances, diffusivities, or mobilities. To calculate proportionality coefficients representative of molecular transport within IEMs, we use the Mackie-Meares model (*156*) to remove tortuosity effects. The Mackie-Meares model predicts the effective slow-down of diffusing species with no adjustable parameters by relying on the water volume fraction, ϕ_w, of the medium. Despite this exacting simplicity, the Mackie-Meares model has been

surprisingly successful at describing transport rates, so we employ it as a reasonable first approximation that will enable an activation entropy analysis within this data set. The Mackie-Meares model was derived for diffusing species by assuming that the tortuosity (τ) represents the only difference between the effective membrane (m) and solution (s) diffusivities, and is traditionally written as:

$$\frac{(D_i^m)^*}{D_i^s} = \frac{1}{\tau^2} = \left(\frac{\phi_w}{2-\phi_w}\right)^2 \qquad \text{S31}$$

To use the Mackie-Meares model, we refine these assumptions: The molecular-scale proportionality coefficient of an ion in the membrane will be allowed to differ from the corresponding value in dilute aqueous solution. The observed, effective proportionality coefficient ($(u_i^m)^*$) can be converted to this molecular-scale proportionality coefficient (u_i^m) using τ^2:

$$u_i^m = \tau^2 (u_i^m)^* \qquad \text{S32}$$

Here, the $*$ designation is used to indicate the macroscopic, effective proportionality coefficient. Through the Nernst-Einstein Relationship (Section S2.3.2), analogous equations can be written for D_i and λ_i. Unlike a direct analogue to the traditional Mackie-Meares model in Equation S31, where it is assumed $u_i^m = u_i^s$, we again emphasize that it is permissible for $u_i^m \neq u_i^s$ in Equation S32 based on our refined assumptions.

The accuracy of the Mackie-Meares model is an inherent assumption when correcting for tortuosity of ion transport in water-swollen membranes. Any deviations from the tortuosity predictions will be interpreted as an entropic effect, even though the true source could be entropy, model error, and other extraneous temperature-invariant phenomena. However, failing to perform a tortuosity correction would severely underestimate experimental values for $\Delta S^\ddagger$ due to misrepresentation of the distance traveled (the δ^2 term in the Eyring-Polanyi equation), so we proceed with this limitation.

S2.3.5 Converting Between Rate Process Parameters

Although ion conduction data can be easily analyzed via an Arrhenius fit, numerous constraints hinder comparable TST analyses. Historically, Arrhenius pre-factors and activation energies have been calculated then mapped 1:1 onto TST parameters (*51*); however, the previous sections have demonstrated that this is not mathematically rigorous. When all the data is readily available, the simplest way to convert between rate analyses is to transform each temperature-variate data point individually. Then, one may perform a TST analysis directly using an appropriate form of the Eyring-Polanyi equation (Equations S19, S28-S30). However, raw data is not always available for this transformation. For these situations, the following conversions will be useful.

The simplest conversion relates to $E_{a,\kappa}$: if the packing of diffusing species in space (C_i) remains constant over the range of temperatures studied, $E_{a,\kappa} = E_{a,\lambda} = E_{a,u}$. In linear IEMs, the swelling can be a strong function of temperature, resulting in C_i that varies with T. For example, this behavior has been well documented for Nafion membranes (*157*). In cross-linked membranes and aqueous solutions, differences in C_i may be quite small over practical temperature ranges (c.f. Figure S3).

For analysis performed using effective proportionality coefficients, which were not corrected for obstruction effects or other phenomena not operating at the molecular level, there is not a generalizable procedure. When tortuosity is the primary concern and is constant over the range of temperature studied, a tortuosity model such as the Mackie-Meares equation can be applied directly to Arrhenius pre-factors or activation entropies:

$$u_0^m = \tau^2 (u_0^m)^* \quad \text{S33}$$

$$\Delta S^{\ddagger} = R \ln(\tau^2) + (\Delta S^{\ddagger})^* \quad \text{S34}$$

The corrected pre-factors may then be converted to activation entropies, whether the pre-factors are u_0 or λ_0. For reported values of $\Delta S^{\ddagger}$ calculated using effective proportionality coefficients, Equation S34 can be used directly. These equations are only valid for a constant tortuosity over the range of temperatures studied, which also implies that no tortuosity correction is needed for values of $E_{a,u}$. When a medium has a tortuosity that varies with temperature, the activation energy will also need correction.

Because diffusion is thermally driven, converting Arrhenius diffusion analyses to TST parameters requires the most attention to detail. First, conversion between $E_{a,D}$ and $E_{a,u}$ is accomplished using Equations S18, S23, and S30:

$$-\frac{E_{a,D}}{R} = \frac{d\ln(D_i)}{dT^{-1}} = \frac{d\ln(k_B T u_i)}{dT^{-1}} = \frac{d}{dT^{-1}}[\ln(u_i) + \ln(T)] \qquad \text{S35}$$

The first term in the rightmost expression evaluates to $-E_{a,u}/R$ by definition. The second term, $-E_{a,f}/R = d\ln(T)/dT^{-1}$, needs to be evaluated over the experimental range of measured diffusivities. The simple result of the derivative is $E_{a,f} = T$, so an averaging function is necessary. When diffusivity data are homogeneously distributed between T_{min} and T_{max}, it should be sufficient to perform an integrated average over the temperature range:

$$E_{a,D} = E_{a,u} + R\frac{\int_{T_{min}^{-1}}^{T_{max}^{-1}} T d(T^{-1})}{\int_{T_{min}^{-1}}^{T_{max}^{-1}} d(T^{-1})} = E_{a,u} + R\frac{\ln(T_{max}^{-1}/T_{min}^{-1})}{T_{max}^{-1} - T_{min}^{-1}} \qquad \text{S36}$$

For the temperature range used in this work (10 – 60 ºC) this expression results in $E_{a,D} - E_{a,u} = 2.55\ \mathrm{kJ/mol}$.

The conversion between D_0 and u_0 is also affected by the temperature range of experimental data. This transformation uses the definition of a linear regression y-intercept alongside Equations S23 and S36:

$$\ln(D_0) = \langle \ln(D_i) \rangle + \frac{E_{a,D}}{R}\langle T^{-1} \rangle = \langle \ln(k_B T u_i) \rangle + \left(\frac{E_{a,u}}{R} + \frac{\ln(T_{max}^{-1}/T_{min}^{-1})}{T_{max}^{-1} - T_{min}^{-1}} \right) \langle T^{-1} \rangle \qquad \text{S37}$$

Here, the bracket terms (e.g., $\langle T^{-1} \rangle$) indicate the mean of an experimental data set. By using an equation for the intercept $\ln(u_0)$ analogous to that of $\ln(D_0)$ in Equation S37, we can isolate the conversion functions from the second expression.

$$\ln(D_0) = \ln(u_0) + \langle \ln(k_B T) \rangle + \frac{\ln(T_{max}^{-1}/T_{min}^{-1})}{T_{max}^{-1} - T_{min}^{-1}} \langle T^{-1} \rangle \qquad \text{S38}$$

The first correction term accounts for the direct temperature scaling in Equation S23, while the second correction term propagates the difference in slope.

For broad temperature ranges, the functional forms of the temperature averaging equations become important; however, linear approximations are sufficient for small temperature ranges. Using simple averages, the following expressions will be reasonably accurate:

$$E_{a,D} \cong E_{a,u} + R\langle T \rangle \qquad \text{S39}$$

$$\ln(D_0) \cong \ln(u_0) + \ln(k_B \langle T \rangle) + 1 \qquad \text{S40}$$

The resulting u_0 pre-factors can then be analyzed via TST directly to calculate $\Delta S^{\ddagger}$. For values of $\Delta S^{\ddagger}$ calculated from D_0 directly, the first correction term is typically accounted for; however, because it has not been widely recognized that $E_{a,D} \neq E_{a,u}$, the second term propagating the difference in the slope is typically missing.

S2.3.6 Controlled Comparisons of Rate Process Parameters

Throughout this manuscript, we control for effects inherently present during ion transport in dilute aqueous solution. So long as membrane and solution data were collected over the same temperature range, many of the quantitative distinctions outlined above are irrelevant for referenced rate process parameters. Equation S36 demonstrates that the conversion between $E_{a,u}$

and $E_{a,D}$ is additive, so when comparing membrane and solution activation energies, $E_{a,u}^{m} - E_{a,u}^{s} = E_{a,\lambda}^{m} - E_{a,\lambda}^{s} = E_{a,D}^{m} - E_{a,D}^{s}$. The same cannot be said of the ratio: $E_{a,D}^{m}/E_{a,D}^{s} \neq E_{a,u}^{m}/E_{a,u}^{s}$.

The temperature conversions are also linear in Equation S38 and thus disappear from pre-factor comparisons. Further, if the hopping distance of ion transport does not differ between membrane and solution, we are able to access the change in activation entropy from membrane to solution without specifying the hopping distance. For membrane and solution data sets collected over identical experimental temperature ranges:

$$\ln\left(\frac{u_0^m}{u_0^s}\right) = \ln\left(\frac{\lambda_0^m}{\lambda_0^s}\right) = \ln\left(\frac{D_0^m}{D_0^s}\right) = \frac{(\Delta S^{\ddagger})^m - (\Delta S^{\ddagger})^s}{R} \qquad \text{S41}$$

S2.4 Arrhenius Fits of Ion Transport in Membranes and Solutions

The unknown parameters of rate process equations (Equations S18 and S19) can be fit to experimental values collected at multiple temperatures in their non-linear forms; however, it is typical to linearize the equations (*158*). Arrhenius coordinates plot ln (D_i) against T^{-1} to easily extract $-E_{a,D}/R$ from the slope and ln (D_0) from the intercept. Eyring coordinates plot ln (D_i/T) against T^{-1} to directly extract $-E_{a,u}/R$ and $\ln(k_B u_0)$. Other transport proportionality coefficients are typically analyzed in Arrhenius coordinates. Because other proportionality coefficients do not represent thermally driven transport, the corresponding Eyring equations may be analyzed directly in Arrhenius coordinates (see Section S2.3). To extract TST parameters representing ion transport in this investigation, we analyze equivalent ionic conductances, λ_i. Per the discussion in Section S2.3.4, effective membrane ionic conductances were transformed into corrected conductance values that are representative of molecular transport rates within the membranes.

To produce a statistically appropriate estimate of rate process parameters from our experimental data, we followed the recommendations of Brauner and Shacham (*158*). These authors caution that an ordinary least-squares linear regression should be avoided in favor of a

general least squares regression. Additionally, for data spanning orders of magnitude where the relative uncertainties are uniform, they recommend that data be linearized into Arrhenius coordinates. Consequently, conductance data were transformed to Arrhenius coordinates, where it was verified that the relative uncertainties in conductance data were uniform. Then, assuming that the uncertainty in temperature was relatively small, we performed a general weighted least-squares linear regression as described by York (*159*). This regression technique weights the residuals by the inverse variance of the data point and re-centers the analysis on the first moment in both x and y coordinates. This methodology allowed for reliable calculation of $E_{a,\lambda}$ and λ_0 for ions in both the IEMs and in dilute aqueous solutions. These parameters were readily transformed into $E_{a,u}$ and $\Delta S^{\ddagger}$ values as described in Section S2.3. To confirm that potential experimental non-linearities causing deviation in the experimental data from a completely linear trend were not significant, the linear fit parameters were also estimated by 5000-point *Parametric* and *Non-Parametric* bootstraps (*97*, *160*). This technique is discussed thoroughly in Section S2.8, and the results of this validation (Table S12) suggest that the Arrhenius fits of this data set are reliable..

We perform a direct comparison between activation energies in the membranes, $E_{a,u}^{m}$ and those in aqueous solution, $E_{a,u}^{s}$. To minimize any biases associated with non-Arrhenius behavior, solution activation energy calculations were generally evaluated using conductances at the same temperatures as the experimental IEM data: 10, 20, 30, 40, 50, and 60 ºC. The aqueous solution conductances of NO_2^- were instead sampled at 5, 10, 15, 20, 25, and 30 ºC to avoid extrapolation beyond the literature data. Although we compare these values qualitatively throughout the manuscript, this limited temperature range may introduce bias in the comparison of activation energies for NO_2^- ions.

S2.5 Intrinsic Ion Properties

Various ion properties proposed to explain SIEs were sourced from the literature. Marcus compiled vast experimental results in his textbooks in 1997 and 2015, titled *Ion Properties* and *Ions in Solution and their Solvation*, respectively (*18*, *86*). These texts provided invaluable and convenient sources for ion information. However, some of the values considered in our study were not available in Marcus's books. Gregory *et al*. reports the radial charge density, þ, which we sourced from their recent study (*16*). Marcus in 1994 reports a geometric hydrogen bonding character of ions which was not fully incorporated into his textbooks (*161*). Finally, Nightingale assembled various ionic radii in 1959, which we utilize to describe the size of ions in this solution (*162*). All these literature properties are listed for the ions under study in Table S15.

S2.6 Correlation Plot Analysis

To identify correlations between observed SIEs and the variables proposed to explain SIEs, we used a correlation plot method to screen for promising options. Because NO_2^- data is scarce in the literature, it was excluded from this analysis. Additionally, without more divalent ion data, valence effects would be difficult to resolve, so the divalent counter-ions were excluded from this analysis. Further work with additional multivalent ion data would be required to understand nuanced effects related to the counter-ion valence. For the remaining ions, the number of ion parameters under consideration outnumbered the number of ions characterized in our experimental dataset. Having more ion properties than ions prevented more sophisticated statistical analyses from being applied to our full dataset. To maximize the statistical power of the five monovalent anions and the six monovalent cations, we needed to select promising ion properties independently from each other.

A linear least-squares regression between observed membrane properties and tabulated ion properties was performed for every pair of independent and dependent variables. The correlations were described using both Pearson correlation coefficients and Spearman correlation coefficients. Both metrics were calculated because the more common Pearson coefficients quantify linear association but contain no internal mechanism to account for unequal spread of data between predictors (called leverage effects). Spearman's method correlates properties by their rank-order, rather than their magnitude, and is therefore able to assess nonlinear dependencies. Nominal agreement between Pearson and Spearman correlation coefficients indicates that leverage differences between the ion properties under study do not meaningfully bias these conclusions.

To ensure that sign differences between anions and cations did not arbitrarily influence the results, only the magnitude of charge density values were used (rather than making all anions negative and all cations positive). Signed values were still utilized for measures of interaction, such as the softness, σ, and hydrogen bond disruption, ΔG_{HB}. To assess our confidence in each correlation, we estimated uncertainty in the correlation coefficients using a 10,000-point *Parametric* bootstrap (*163*) on the measured activation energies, which are approximately normally distributed within each ion's six replicate measurements. This technique is introduced fully in Section S2.8. Because the ion property values are tabulated, we fixed these data during this process. With each bootstrap iteration, Pearson and Spearman correlation coefficients were calculated, building an empirical sampling distribution that does not rely on normality or continuity of the predictor variables.

S2.7 Principal Component Analysis (PCA)

Once statistically significant factors had been selected via the Pearson correlation coefficient screening, we used PCA to further analyze the correlated ion factors. This second-level

analysis simplifies the physical interpretation of phenomena contributing to differences in activation energies of transport in these IEMs. PCA methods in chemical sciences have been covered extensively in Malinowski's seminal textbook (*164*). PCA reduces the dimensionality of complex data sets by grouping modes of variance into principal components (PCs). Although all components are required to exhaustively account for all modes of variance in the data set, often PCs are more efficient than measurable variables at explaining data variance. Typically, two or three PCs can account for nearly all the variance in an interconnected data set, even though there may be significantly more than three measured variables. To identify the number of PCs required to explain the variance in a data set, it is standard to perform a *Skree Plot* analysis to assess PC significance (*164*). In all our PCA analyses, only the first two PCs were required to explain >90% of the variance in the data set. To show the relationship between variables in these PCs, a biplot is used to map ion properties onto the first two principal components (PC1 and PC2). Factors grouped close to each other on the biplot are considered highly correlated within the context of the analyzed variables, while orthogonality indicates less similar modes of variance.

S2.8 Bootstrapping Methods for Uncertainty Quantification

Bootstrapping is a widely used method to assess the confidence in calculated and fitted parameters (*97*, *160*, *163*). Highly irregular data (non-normal distributions) or highly co-dependent fitting parameters invalidate assumptions used in standard methods used to calculate and propagate uncertainty, giving rise to a host of alternate approaches which may handle these difficult circumstances (*165*). Among the most popular techniques, bootstrapping functions as a re-sampling method to assess how small systemic changes to the data drawn from re-sampling the error of samples affect the results and quality of optimization problems. The overlapping peak deconvolutions, correlated Arrhenius parameters, and non-continuous collections of ion properties

each either violate statistical normality, exhibit parameter collinearity, or demonstrate small systematic changes that would affect the confidence interval calculated using typical population statistics-based formulae. Therefore, all these situations require bootstrapping solutions to properly assess our confidence in these results.

Bootstrapping requires a method to introduce random error into the data set (*97*, *98*, *160*, *163*). The best method of introducing error depends upon the nature of the data set. The most common approaches are *XY*, *Non-Parametric*, *Parametric,* and *Wild*, which differ in their assumptions about the underlying error distribution of measured values in a statistical problem. *Non-Parametric* bootstraps are often the most robust, as they only use re-sampled values (drawn from replicate data or a similar source) with no assumptions regarding the underlying noise distribution. *Parametric* bootstraps assume that the underlying noise distribution is normal, and so may work better in cases where this assumption is reasonable. In each case, the original data set is re-sampled by systematically replacing the original data and re-fitting the optimization problem. By re-fitting the data using a large (>1000) number of these systemic re-samplings, bootstrapping techniques estimate how robust the fitting parameters are and thus give insight into the uncertainty about their optimal values. The ease of performing a large number of re-samplings and the lack of external information required (since bootstrapping methods only rely on the measured data) make bootstrapping a favored technique to estimate population statistics with reasonable accuracy but without relying on assumptions such as normal variance and non-collinearity between factors.

To demonstrate a method of introducing error, the *Parametric* bootstrapping technique is outlined below. Data are randomly drawn from the vicinity of the average according to:

$$x^* \sim \mathcal{N}\left(\bar{x}, \frac{\sigma}{\sqrt{n}}\right) \quad \text{S42}$$

Here, x^* indicates the re-distributed measured variable, $\bar{x}$ is the measured average value, σ is the standard deviation, and n is the number of samples measured. This sampling is performed for each measured variable in an optimization problem (each data point or each sampling energy along a spectrum) to produce a statistically likely assembly of data which is not necessarily the exact values measured. The optimization problem is then re-run, and the results are tabulated. When this is done a large number of times, the variations observed in the fitting parameters represent how sensitive they are to the data being precisely the mean values, as opposed to other reasonably likely values within their confidence interval. Other bootstrapping methods perform the same process but with a different re-sampling function. For example, *Non-Parametric* bootstraps do not assume an error distribution, but instead uses the replicates already contributing to a data point to simulate experimental error without *a priori* knowledge of its parametric form.

S3 Molecular Dynamics Methods

S3.1 Simulation Parameterization

Molecular dynamics (MD) simulations were performed using the Large-scale Atomic/Molecular Massively Parallel Simulator (LAMMPS) (*42*). Water was modeled using the transferable intermolecular potential with four points (TIP4P/2005) (*45*). This water model was used because of its accurate representation of the density, coordination number, surface tension, dielectric constant, and self-diffusion coefficient of bulk water (*166*). The parameterization of alkali and halide ions for the TIP4P/Ew water model was used as it accurately replicates solution properties (e.g., solvation energy, coordination number, hydrated radius, and solute diffusivity) in simulations containing TIP4P/2005 water (*46*, *47*). Non-bonded interactions and bonds in multiatomic molecules were parameterized using the optimized potentials for liquid simulation (OPLS) force field (*48*, *49*). Non-bonded interactions were modeled via Lennard Jones and

Coulombic parameters. Bonded interactions include interatomic bonds (2-body), bond angles (3-body), and dihedral angles and improper dihedral angles (4-body). Pairwise interactions were computed for both Lennard Jones and Coulombic potentials up to a cutoff of 10 Å. Beyond this cutoff, Coulombic pairwise interactions were evaluated using the particle-particle particle-mesh algorithm.

Multiatomic molecules included the monomers shown in Fig. 1: [2-(Methacryloyloxy)ethyl] trimethylammonium (MOETMA), 3-Sulfopropyl methacrylate (SPM), and glycerol dimethacrylate (GDMA). Additional multiatomic molecules included: tetramethylammonium (NMe_4^+), ammonium (NH_4^+), nitrate (NO_3^-), and water. Alkali metal ions included: lithium (Li^+), sodium (Na^+), potassium (K^+), and cesium (Cs^+). Halide ions included: fluoride (F^-), chloride (Cl^-), bromide (Br^-), and iodide (I^-).

The isothermal-isobaric (NPT) ensemble was used to hold the number of atoms (*N*), domain pressure (*P*), and domain temperature (*T*) constant (*167*). The Nosé-Hoover style thermostat and barostat were used to control temperature and pressure, respectively. The time step during simulation was 1 fs.

S3.2 Simulation Design

To generate IEM simulation domains suitable for equilibrium sampling, subsequent steps of pre-polymer solution equilibration, polymerization, ion exchange, swelling, and equilibration were performed. Figure 1c in the manuscript depicts this workflow visually. To access LAMMPS input and parameter files used in these simulations, readers are directed to Zenodo (*168*). Simulation output data will be made available by request due to the large file sizes.

S3.2.1 Pre-polymer Solution Equilibration

IEMs were prepared *in silico* via polymerization of a charged monomer and cross-linker in water. The simulation was designed to match the experimental conditions as closely as possible. First, an oversized domain (250×250×250 $Å^3$) was randomly packed with GDMA cross-linkers, charged monomers, counter-ions, and water. For the AEMs, the charged monomer was MOETMA, and the counter-ion was Cl^-. For the CEMs, the charged group monomer was SPM, and the counter-ion was K^+.

After packing, the domain was equilibrated using an NPT ensemble for 3 ns. During equilibration, the domain was held at 1 atm and 300 K. Within this ensemble, domain volume is allowed to change to maintain the applied pressure. Equilibration continued until the pre-polymer solution density reached a constant value.

S3.2.2 IEM Polymerization and Equilibration

During synthesis of the lab-made IEMs, the methacrylate chain growth reaction was initiated using a free radical initiator, V-50, and reactions were promoted via elevated temperature. To accurately replicate in-lab polymerization conditions, an NPT ensemble was used to increase the domain temperature to 358.15 K while maintaining the pressure at 1 atm. The number of initiated methacrylate groups was determined by assuming that all initiator molecules added to the experimental pre-polymer solution (1% mass initiator/mass polymer) were consumed. The ratio of initiated methacrylate groups on GDMA molecules to those on the charged monomers was set proportional to their concentrations in the simulation. No GDMA molecules had both methacrylate groups initiated. All initiations were performed at the beginning of the simulation and no new initiations occurred during polymerization.

Polymerization of the IEM via chain initiation and radical propagation was performed using REACTER (*43*, *44*). REACTER enables the use of reactive MD for select reactions, including growth of networked methacrylate polymers (*43*, *44*). To recreate the chain initiation behavior, only the initiated methacrylate groups were candidates to initiate propagation reactions. To enable chain growth, after a single successful propagation reaction, the newly added methacrylate group was designated as initiated and therefore a possible candidate for a new propagation reaction.

Characterization of the lab-made AEM and CEM polymer gel fractions yielded values greater than 95% (Table S5), indicating that the final system is highly entangled and/or crosslinked. Accordingly, polymerization was carried out until fewer than 10 new polymerization reactions occurred per simulated ns. This threshold was deemed a sufficient trade-off between the crosslinking of the final polymer and the investment of computational effort, and produced gel fractions that were comparable with the experimental results (Table S5).

Once polymerization was completed, all remaining unreacted monomers were deleted. Then, the ion form of the membrane was converted by replacing the counter-ion (Cl^- or K^+), if necessary, and inserting/deleting water molecules to match the corresponding experimental water uptake (Table S6). Although this procedure does not allow the network to swell to equilibrium naturally, the computational resources required to implement such results are were deemed too intensive for the scope of this study (*169*). After these changes, IEMs were equilibrated using an NPT ensemble at 300 K and 1 atm for 1 ns.

S3.2.3 Infinite Dilution Equilibration

To accurately compare the interaction behavior of ions and water, infinite dilution simulations of the counter-ions of interest were also performed. Infinite dilution simulations were

performed using 100 counter-ions and 52,300 water molecules. Simulations were initialized and equilibrated via a similar process to the IEMs.

S3.2.4 Equilibrium Sampling

Equilibrium sampling of the equilibrated IEM and infinite dilution domains was performed using an NPT ensemble 300 K and 1 atm for 5 ns. During equilibrium sampling, trajectory data of the water molecules, counter-ions, and (for IEM simulations) polymer networks were saved every 1000 fs. The time-resolved coordination numbers of species were monitored to ensure the system was at equilibrium. These data were then averaged over the full duration of the equilibrium sampling.

S3.3 Simulation Analyses

S3.3.1 Radial Distribution Functions

Within LAMMPS, the command *compute rdf* was utilized to determine radial distribution functions (RDFs, $g(r)$). For a central atom/candidate atom pair, $g(r)$ is the probability of finding a candidate atom within a shell of radius r around the central atom ($\rho(r)$) normalized against the probability of finding a candidate atom in that shell if the density of the candidate atom within that shell was equivalent to the bulk density of the candidate atom in the simulation (ρ_0). With this definition, $g(\infty) = 1$. These functions were evaluated at all simulated timesteps with a radial step size of 0.01 Å up to the pairwise cutoff of 10 Å.

RDFs involving water were calculated using the position of oxygen within the water molecules. RDFs involving multiatomic counter-ions and fixed charge groups were calculated using the position of the central atom of the ion: nitrogen for NH_4^+, NMe_4^+, NO_3^-, and $RNMe_3^+$ in MOETMA; sulfur for RSO_3^- in SPM. RDFs for counter-ion/water pairs were determined for both

IEM and infinite dilution simulations. Additionally, RDFs for fixed charge/water and fixed charge/counter-ion pairs were determined for the IEM simulations.

The sizes of primary coordination shells (CN) were determined by the location of minima in the RDFs. The density of species was integrated from the center atom to the radius of the coordination shell to extract the coordination number.

S3.3.2 Potential of Mean Force

The radial distance profile for the potential of mean force (PMF) between a central atom and a candidate atom, $W(r)$ can be determined as (*170*, *171*):

$$W(r) = -k_B T \ln P(r) \qquad \text{S43}$$

Here, k_B is the Boltzmann constant and $P(r)$ is the probability of a candidate atom being located a distance r from the central atom. The probability function can be determined from $g(r)$ using the following equation:

$$P(r) = \frac{g(r)}{\sum g(r)} \qquad \text{S44}$$

From the PMF profiles, three key metrics were calculated. First, the interaction potential between a central and candidate molecule located in a coordination shell, per candidate molecule, could be extracted from the local minimum associated with that coordination shell. Second, the same value normalized per central molecule could be calculated by integrating the product of coordination number and interaction energy.

$$W_{coord} = \int_0^{r_{coord}} W(r) * CN(r)\, dr \qquad \text{S45}$$

Here, W_{coord} and r_{coord} are the PMF and radius associated with a given coordination shell around the centrally queried atom. Finally, the energy barrier faced by a candidate molecule leaving the

vicinity of a central molecule can be taken as the difference between the corresponding PMF local minimum and the next-closest local maximum.

S3.4 Validation of Simulated Membranes

S3.4.1 Pre-Polymer Solutions

To ensure that pre-polymer simulations were resolved adequately and were comparable to experimental pre-polymer solutions, system variables (temperature, pressure, density, and total energy) were monitored during equilibration. Figure S4 depicts these system variables over time for the AEM and CEM pre-polymer solution simulations, and Table S4 contains the final characteristics of the simulated pre-polymer solutions.

The temperature and total energy remained constant and well-controlled within the limits of the MD simulation parameters. The pressures exhibited higher variance, as is expected from liquid solvents. For nearly incompressible liquids such as water, small changes in domain volume can result in significant pressure fluctuations. For both the AEM and CEM pre-polymer solutions, the simulated densities matched the experimentally determined densities within the uncertainties of the experimental measurements.

S3.4.2 Polymerization

To ensure that simulation dynamics were adequately resolved during polymerization, we again monitored system level variables as a function of time (Figure S6), which indicated that the reactions did not disrupt the accuracy of the simulation. We also monitored the extent of reaction as a function of time (Figure S7). Initially chain growth proceeded rapidly, but as the polymerization approached completion, the rate of reaction slowed. The polymerization was deemed complete once the rate of reaction reached less than 10 chain growth steps per simulated ns. Using this criterion, AEM polymerization lasted 15 ns and CEM polymerization lasted 12 ns.

S3.4.3 Equilibration

Table S5 lists characteristics of the simulated IEMs after polymerization. Both simulations contained unreacted GDMA molecules and charged monomers that needed to be removed from the simulation. Removing non-networked monomers allowed for calculation of the IECs, which were slightly higher than the experimentally determined values. The IEC differences must be attributable to co-monomer polymerization, because the IEC relates to the molecular composition of the polymer backbone. Nevertheless, these differences were small, giving us confidence in the simulated co-polymerization.

Water was then added to the Cl^- and K^+ form IEM simulations to match the respective experimental water uptakes of 0.833 and 0.813 g[water]/g[dry polymer]. Following the brief equilibration, data were collected via the equilibrium sampling techniques. The coordination numbers of ions, charge groups, and waters obtained during sampling of AEMs and CEMs, demonstrate consistency over time, indicating stability of the local structure while sampling was performed. Representative profiles for two AEM and two CEM simulations are shown in Figure S8 and Figure S9, respectively. For the remainder of the analysis, these data were averaged across the entire sampling period.

After equilibration, the total hydrated densities of the simulations were recorded for comparison with experimental values (Table S5). The simulated CEM exhibited an equilibrated hydrated density that was the same as the experimentally determined value, within the uncertainty of the experimental data. Meanwhile, the simulated AEM exhibited a lower density than the lab-made AEM. Although the IEC discrepancy carries over between counter-ion forms, the hydrated density changes as the simulations are re-equilibrated with the various counter-ion forms. Therefore, we consider the hydrated density of all 11 counter-ion forms (Table S6 and Table S7)

to compare the molecular arrangement of the simulated IEMs with experimental values. The hydrated density of the simulated AEMs and CEMs fell within the uncertainty of the experimental data for most of the anions and cations analyzed, respectively. The simulated CEM densities were higher than the experimentally measured values for Cs^+ and NH_4^+, but within error for Li^+, Na^+, K^+, and NMe_4^+ form membrane simulations. Meanwhile, the simulated AEM densities were lower than the experimentally measured values for Cl^- and I^-, but within error for F^-, Br^-, and NO_3^- form membrane simulations. Overall, these results suggest that the simulated membranes provide a good representation of the lab-made membranes that were characterized experimentally.

S4 Supplementary Figures

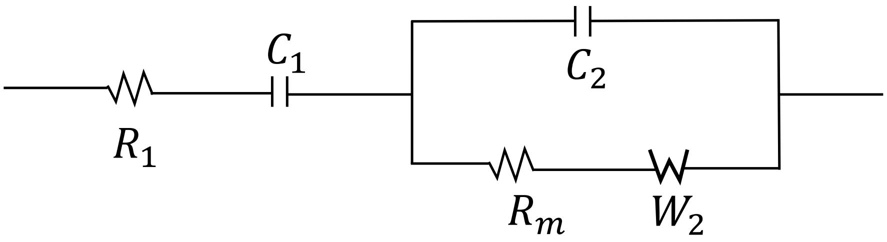

Figure S1: EIS equivalent circuit. A modified version of the Randles equivalent circuit that was used to extract the membrane resistance (R_m) from measured electrochemical impedance spectroscopy (EIS) data.

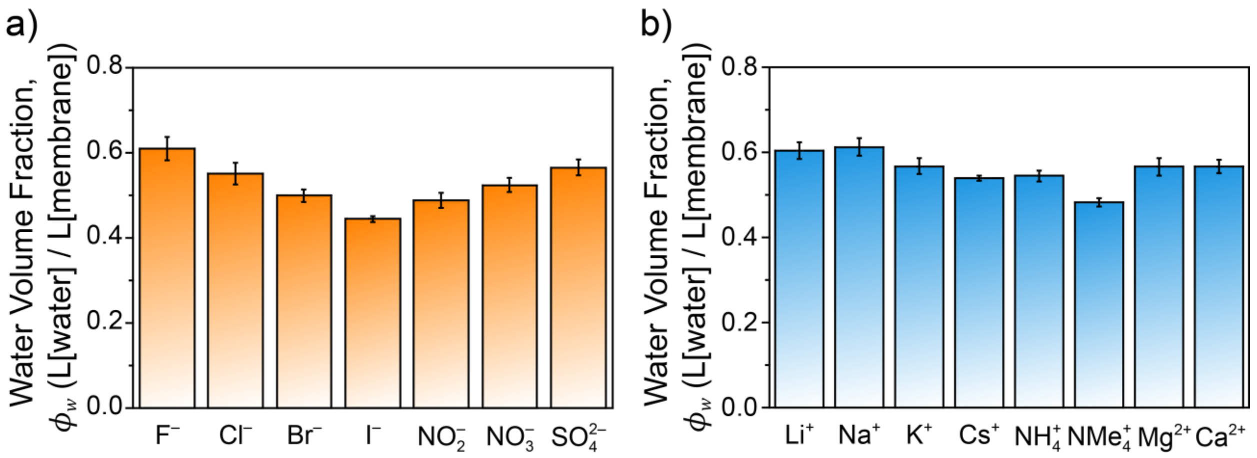

Figure S2: Membrane water volume fractions. The water volume fraction (ϕ_w) of a) AEMs and b) CEMs in each counter-ion form under study at room temperature (21 ± 1 ºC). Error bars represent the standard deviation of measurements made on at least five independent samples.

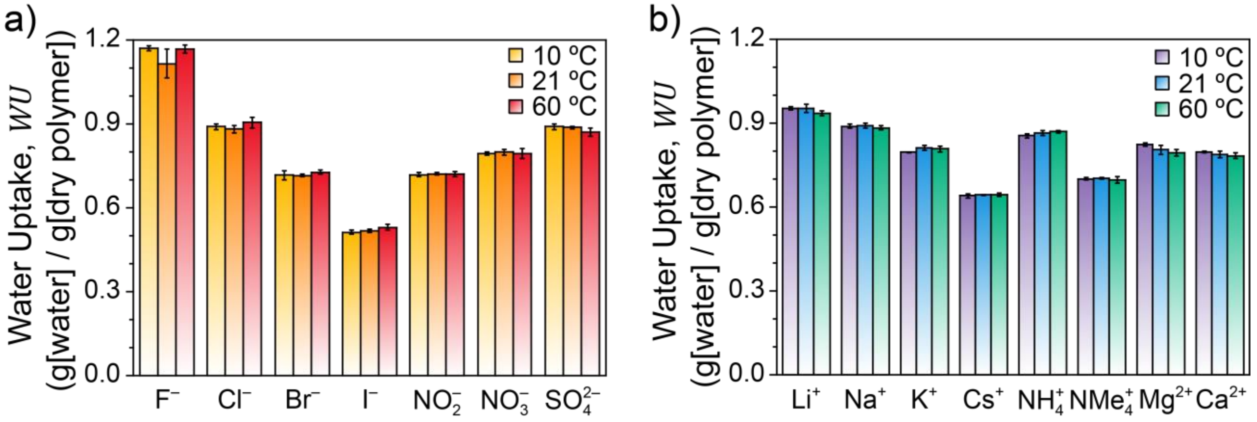

Figure S3: Membrane water uptake at various temperatures. The water uptake (WU) of a) AEMs and b) CEMs in each counter-ion form under study collected at 10 ºC, room temperature (21 ± 1 ºC), and 60 ºC. Error bars represent the standard deviation of measurements made on at least five independent samples.

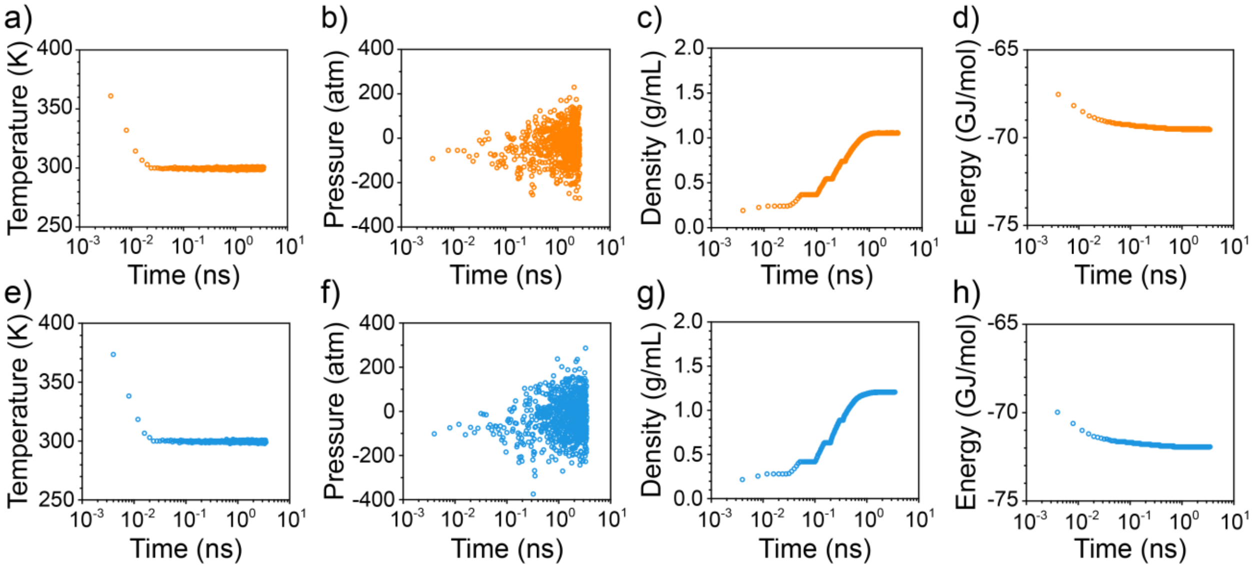

Figure S4: Simulated pre-polymer solutions. System-level variables during equilibration of the simulated pre-polymer solution for the AEM (a–d) and CEM (e–h). Monitored variables include the temperature (a and e), pressure (b and f), density (c and g), and total system energy (d and h).

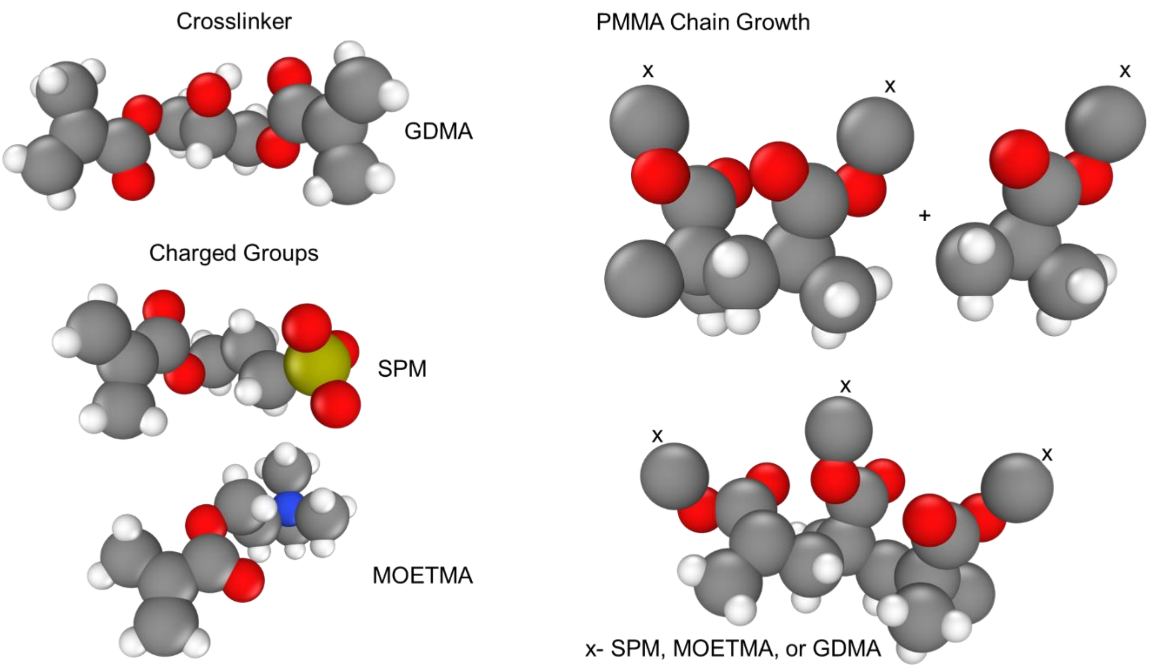

Figure S5: Simulated monomers and repeat units. Representative cross-linkers and charged monomers, and the mechanism of the methacrylate backbone chain growth. For clarity, truncated versions of the reacting molecules are depicted.

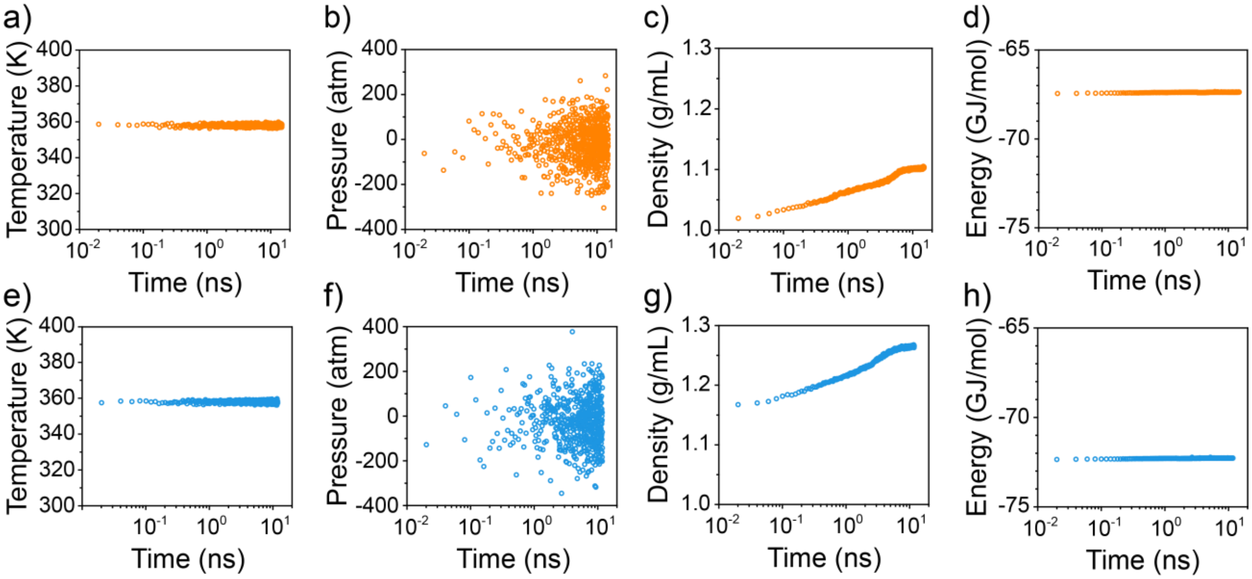

Figure S6: Simulated polymerization. System-level variables during the polymerization reaction for the AEM (a–d) and CEM (e–h). Monitored variables include the temperature (a and e), pressure (b and f), density (c and g), and total energy (d and h).

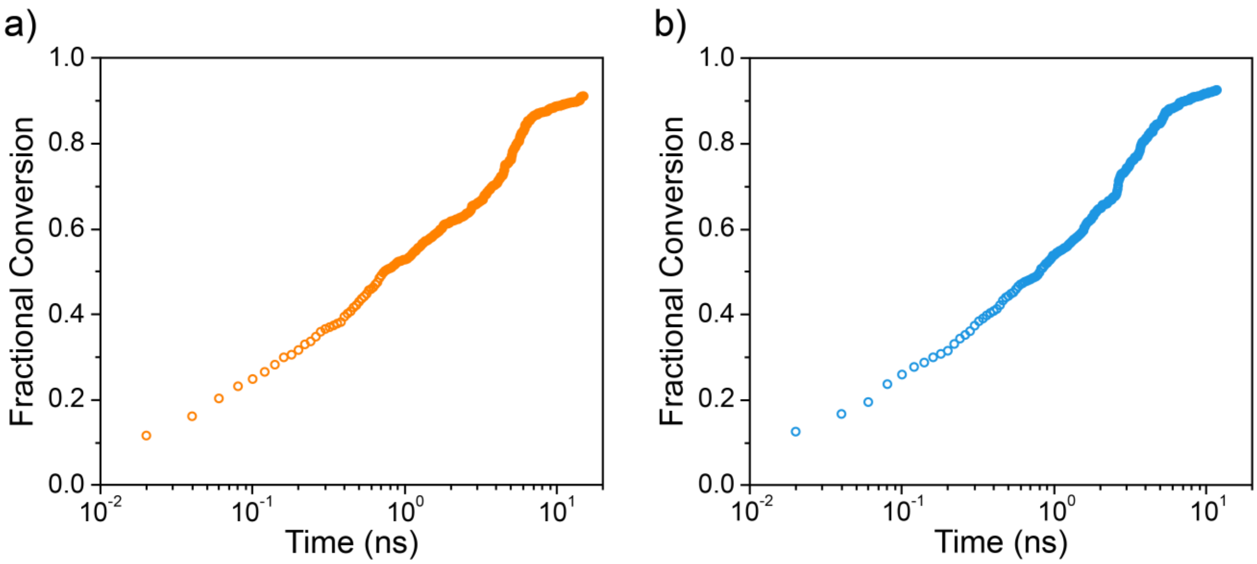

Figure S7: Simulated chain growth progression. The extent of reaction for methacrylate chain growth steps during polymerization. Progress is shown for a) the AEM and b) the CEM simulation.

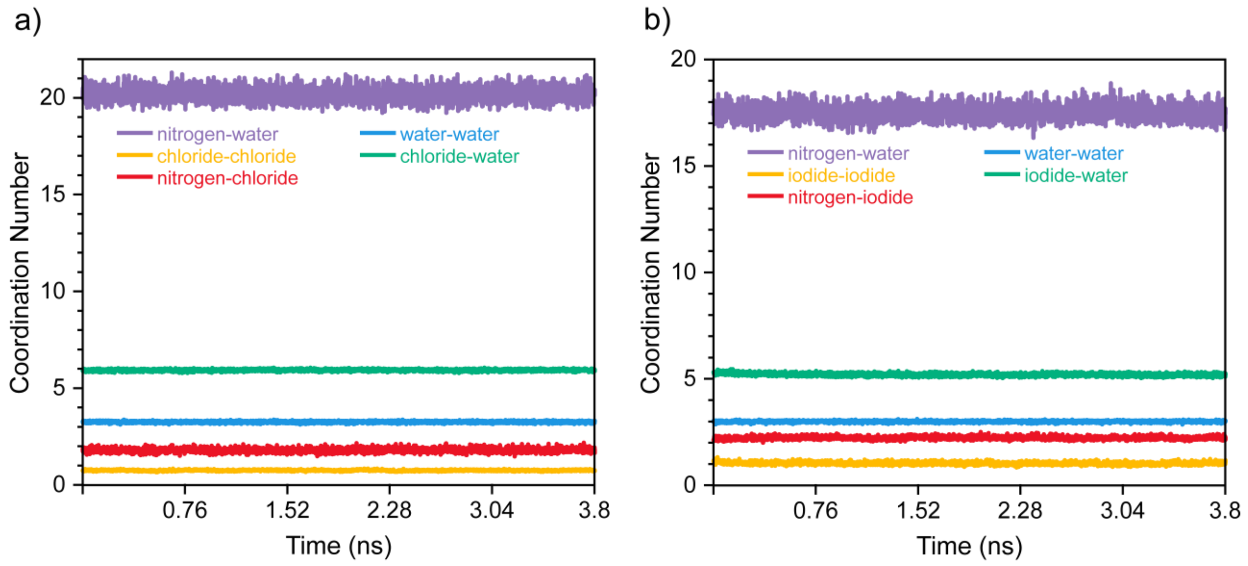

Figure S8: Time-resolved equilibrium sampling of AEMs. Representative plots of coordination numbers versus time for various pairs during the initial 3.8 ns of AEM simulations with a) Cl^- counter-ions and b) I^- counter-ions. Nitrogen atoms correspond to the ammonium groups of the MOETMA monomer.

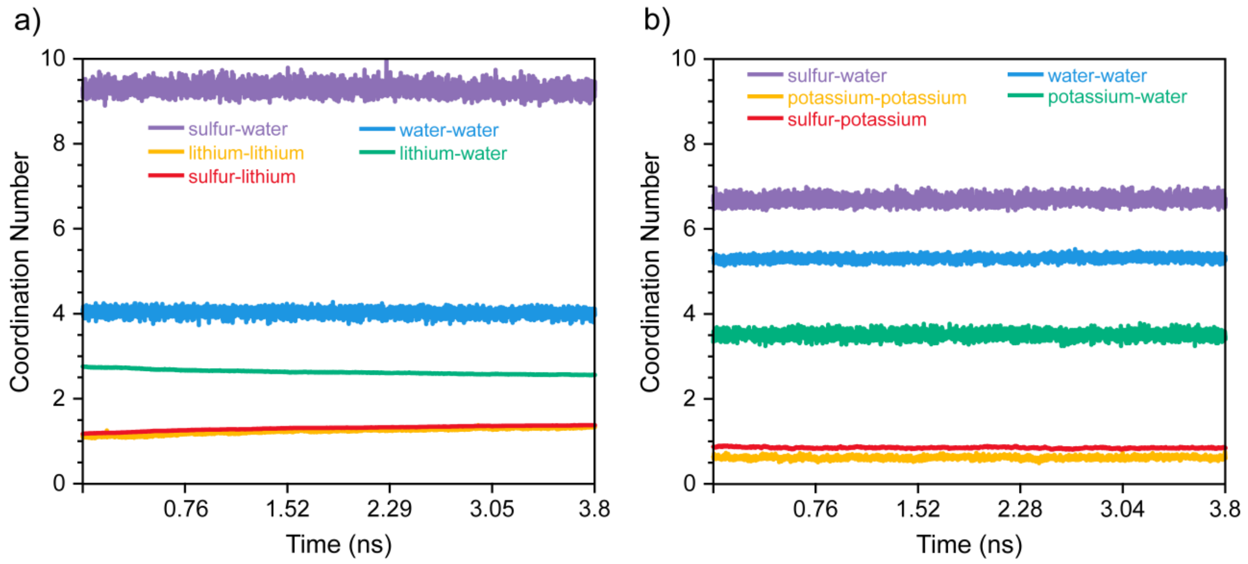

Figure S9: Time-resolved equilibrium sampling of CEMs. Representative plots of coordination numbers versus time for various pairs during the initial 3.8 ns of CEM simulations with a) Li^+ counter-ions and b) K^+ counter-ions. Sulfur atoms correspond to the sulfonate groups of the SPM monomer.

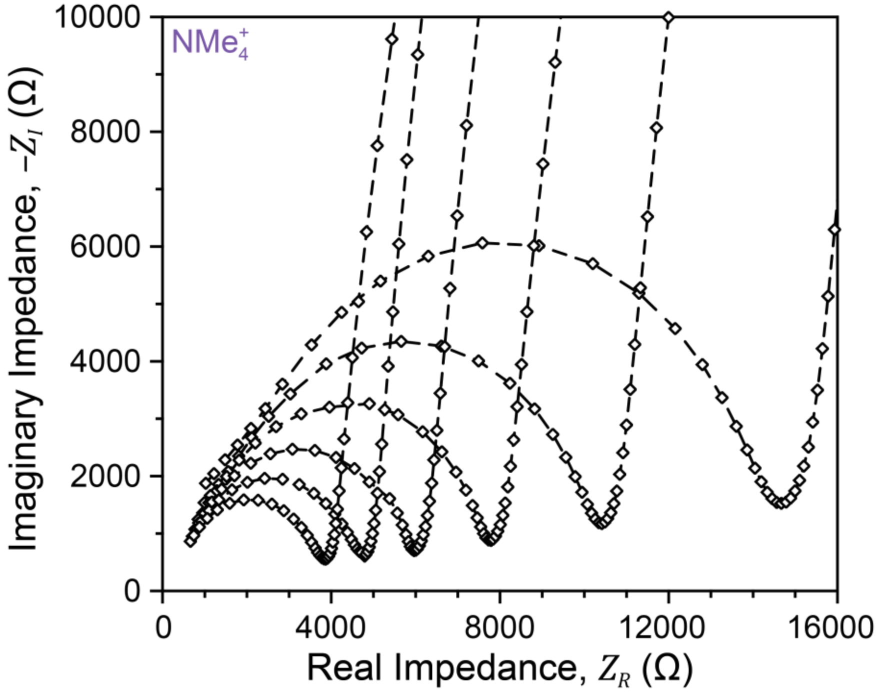

Figure S10: Example Nyquist plots. Typical Nyquist plots obtained during in-plane ionic conductivity measurements collected at 10, 20, 30, 40, 50, and 60 ºC. Greater temperatures yield smaller semi-circles at the left side of the plot, while lower temperatures yield larger semi-circles at the right side of the plot. These representative scans are for a CEM in the NMe_4^+ form.

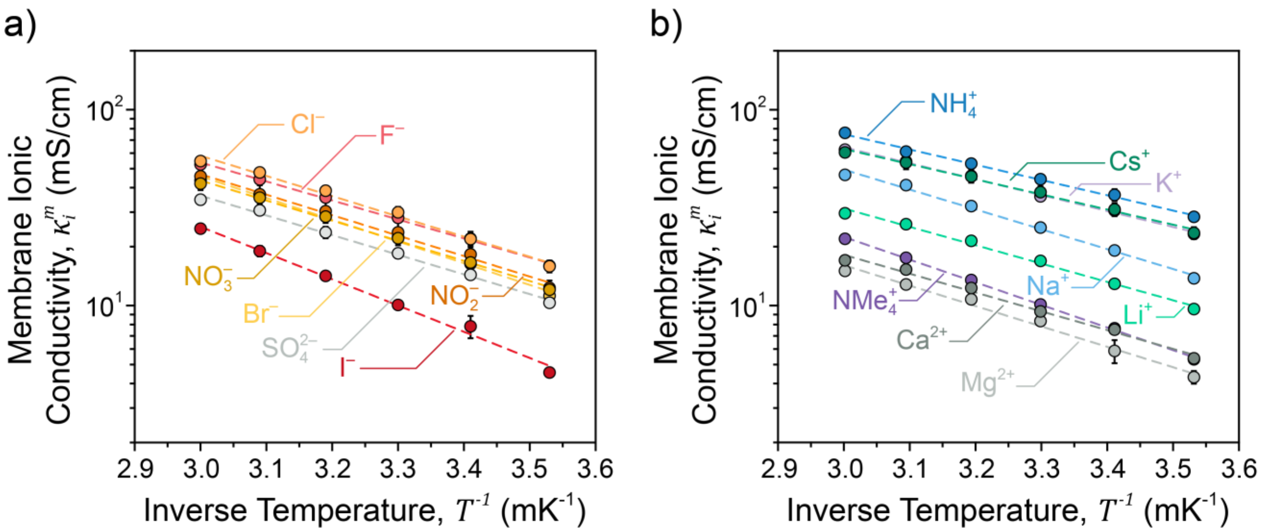

Figure S11: Arrhenius plots of membrane ionic conductivities. The logarithm of ionic conductivities (κ_i^m) plotted against the inverse temperature (T^{-1}) for a) anions and b) cations in the IEMs. Error bars represent the standard deviation of four independent conductivity measurements at each temperature.

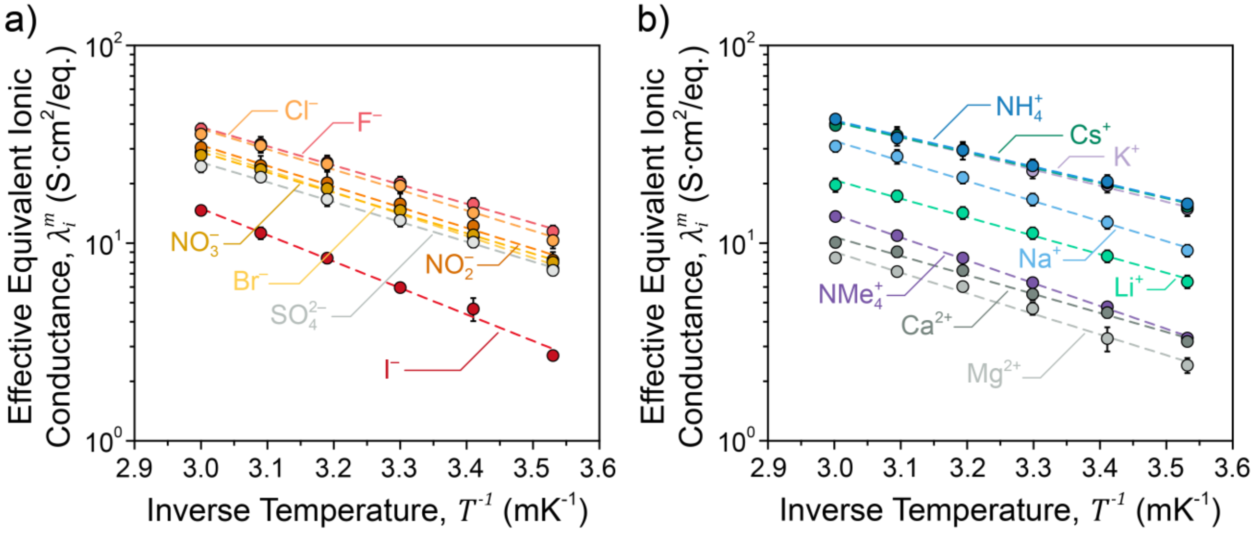

Figure S12: Arrhenius plots of effective equivalent ionic conductances for ions in the IEMs. The logarithm of effective equivalent ionic conductance (λ_i^m) plotted against the inverse temperature (T^{-1}) for a) anions and b) cations in the IEMs. Error bars represent the standard deviation of the data, calculated using standard propagation of uncertainty methods.

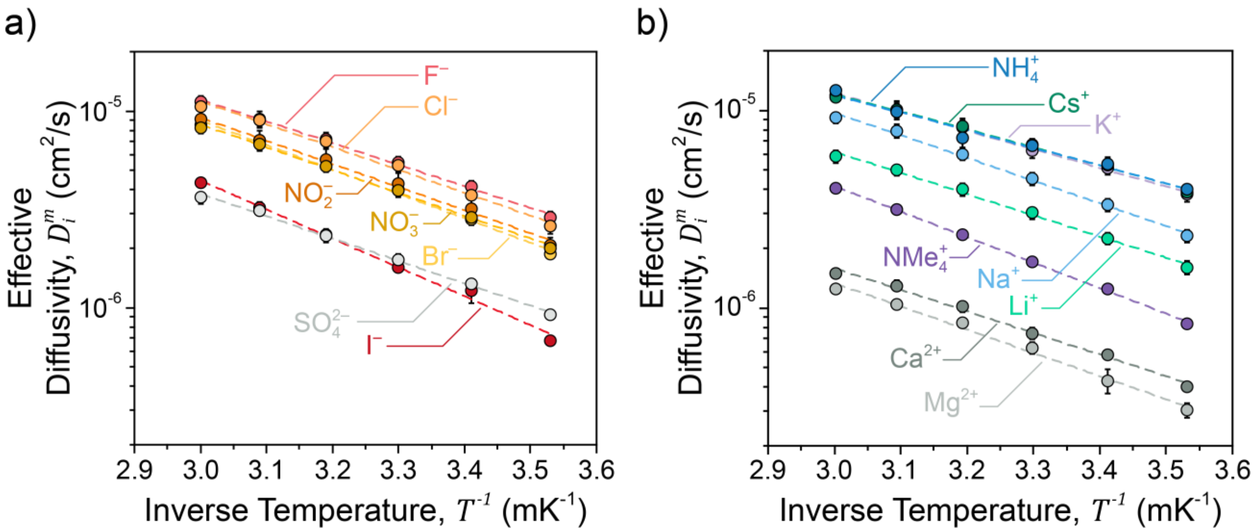

Figure S13: Arrhenius plots of effective ion diffusivities in the IEMs. The logarithm of effective ion diffusivities (D_i^m) plotted against the inverse temperature (T^{-1}) for a) anions and b) cations in the IEMs. Error bars represent the standard deviation of the data, calculated using standard propagation of uncertainty methods.

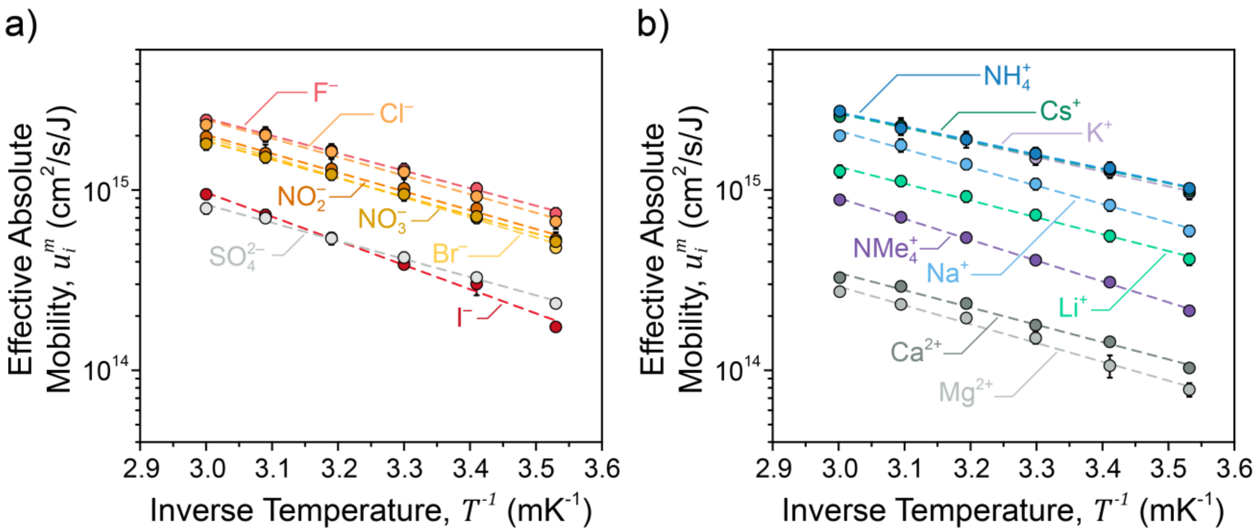

Figure S14: Arrhenius plots of effective absolute mobilities for ions in the IEMs. The logarithm of effective absolute mobilities (u_i^m) plotted against the inverse temperature (T^{-1}) for a) anions and b) cations in the IEMs. Error bars represent the standard deviation of the data, calculated using standard propagation of uncertainty methods.

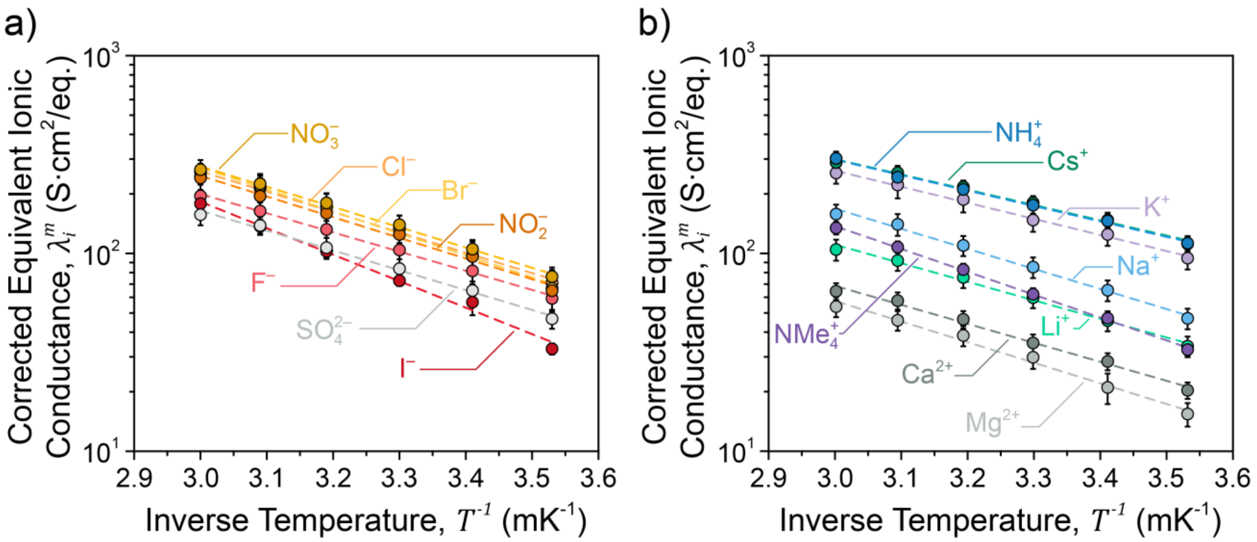

Figure S15: Arrhenius plots of corrected equivalent ionic conductances for ions in the IEMs. The logarithm of equivalent ionic conductances (λ_i^m), which have been corrected for obstruction effects via the Mackie-Meares model (Section S2.3.4), plotted against the inverse temperature (T^{-1}) for a) anions and b) cations in the IEMs. Error bars represent the standard deviation of the data, calculated using standard propagation of uncertainty methods.

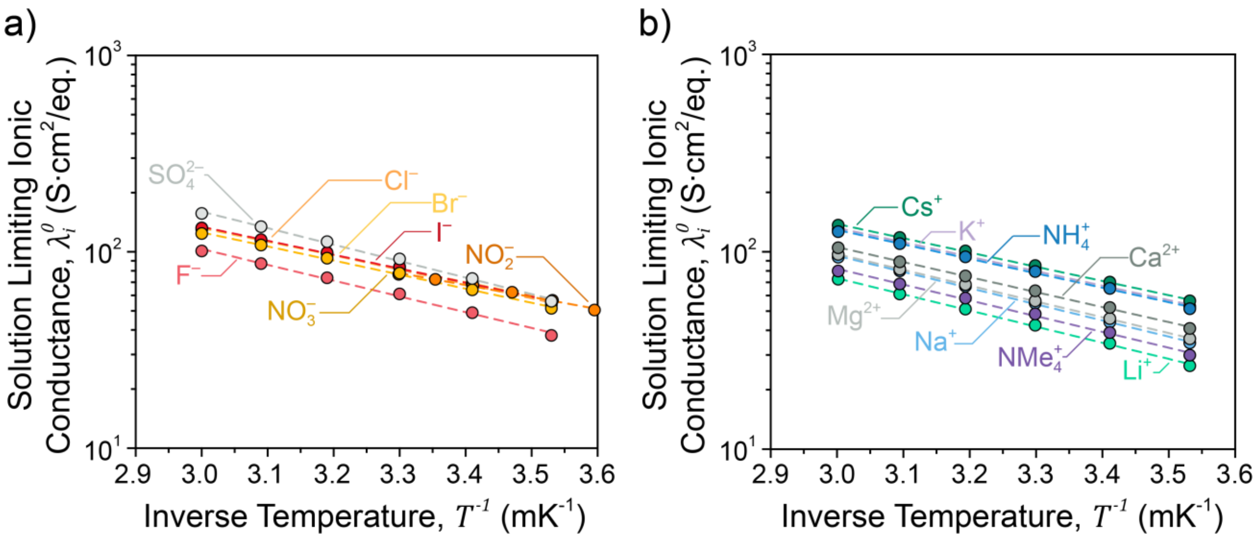

Figure S16: Arrhenius plots of limiting equivalent ionic conductances for ions in dilute aqueous solution. The logarithm of the limiting equivalent ionic conductance (λ_i^0) plotted against the inverse temperature (T^{-1}) for a) anions and b) cations in infinitely dilute aqueous solution. Data were sourced according to Section S2.2.

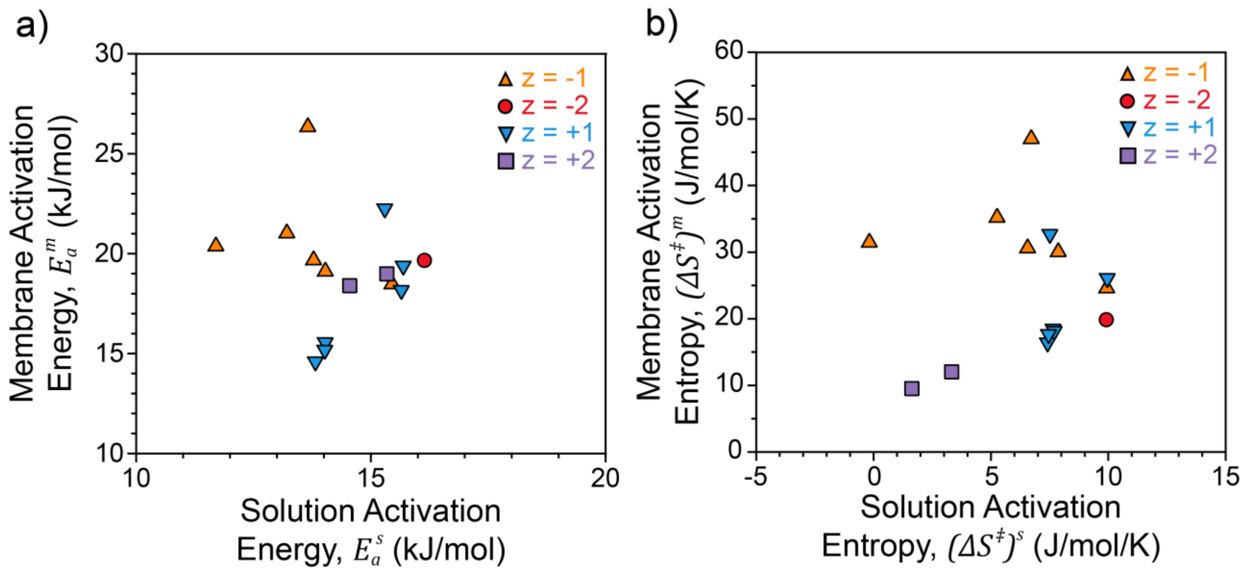

Figure S17: Independence of membrane and solution transport energetics. Correlation plots between the membrane and solution activation energies (a) and activation entropies (b) demonstrate minimal relationship between the two properties.

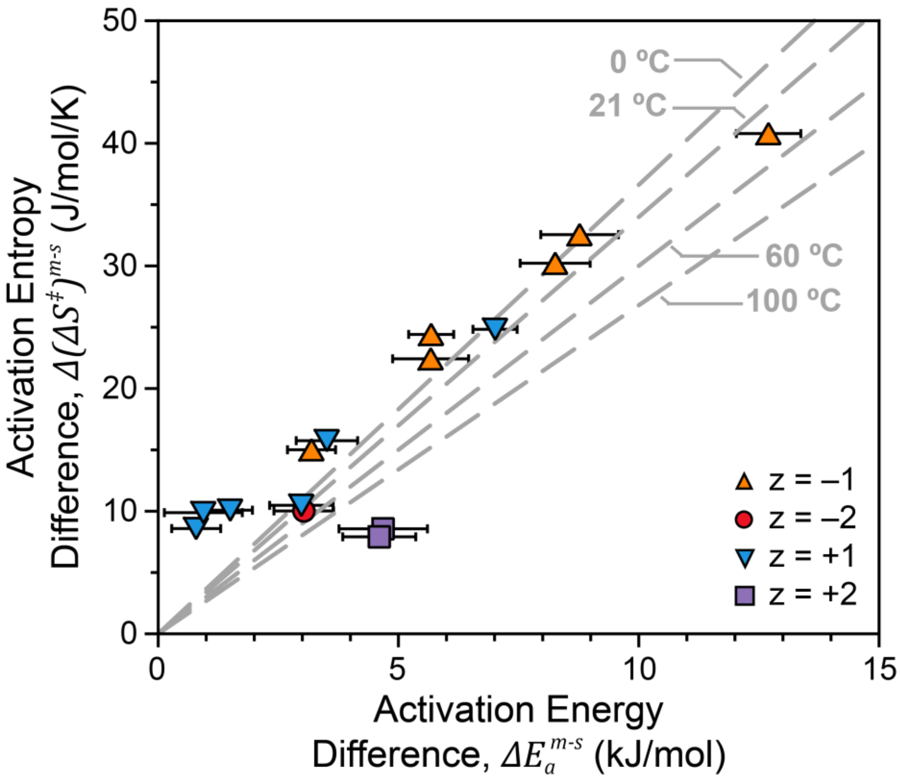

Figure S18: Compensation parity lines. The linear free energy plot of membrane effects ion transport with added parity lines for 0, 10, 60, and 100 ºC. The parity lines represent points where the free energy for ion transport ($\Delta G^{\ddagger}$) is equivalent for ions in the IEMs and in dilute aqueous solution. Data points above each parity line represent more energetically favorable transport for ions in the membrane at that temperature. Data points below each parity line represent more energetically favorable transport for ions in dilute aqueous solution at that temperature. Uncertainties were calculated from standard errors of the activation energies and entropies using standard propagation of uncertainty methods.

Notes: To the extent that the obstruction correction can be relied on, ions appear to transport more easily (i.e., experience a lower free energy barrier to transport) in the membranes than in an aqueous solution. Entropic effects are more prevalent at high temperatures, while enthalpic effects

dominate rate processes at low temperatures; thus, the slope and intercept of the linear free energy relationship in Fig. 3D dictates which effect dominates at each temperature. The isotherms in Figure S18 demonstrate the temperature at which membrane free energies of transport become lower than those of aqueous solution. They demonstrate that entropic effects in these IEMs overpower enthalpic effects at 21 ºC for many ions in this study. Using the slope and intercept calculated for monovalent ions studied here, the intersection should occur at 21 ºC for $\Delta E_a^{m-s} = 11\ kJ/mol$. A cross-section of the free energy analysis is shown below in Figure S19.

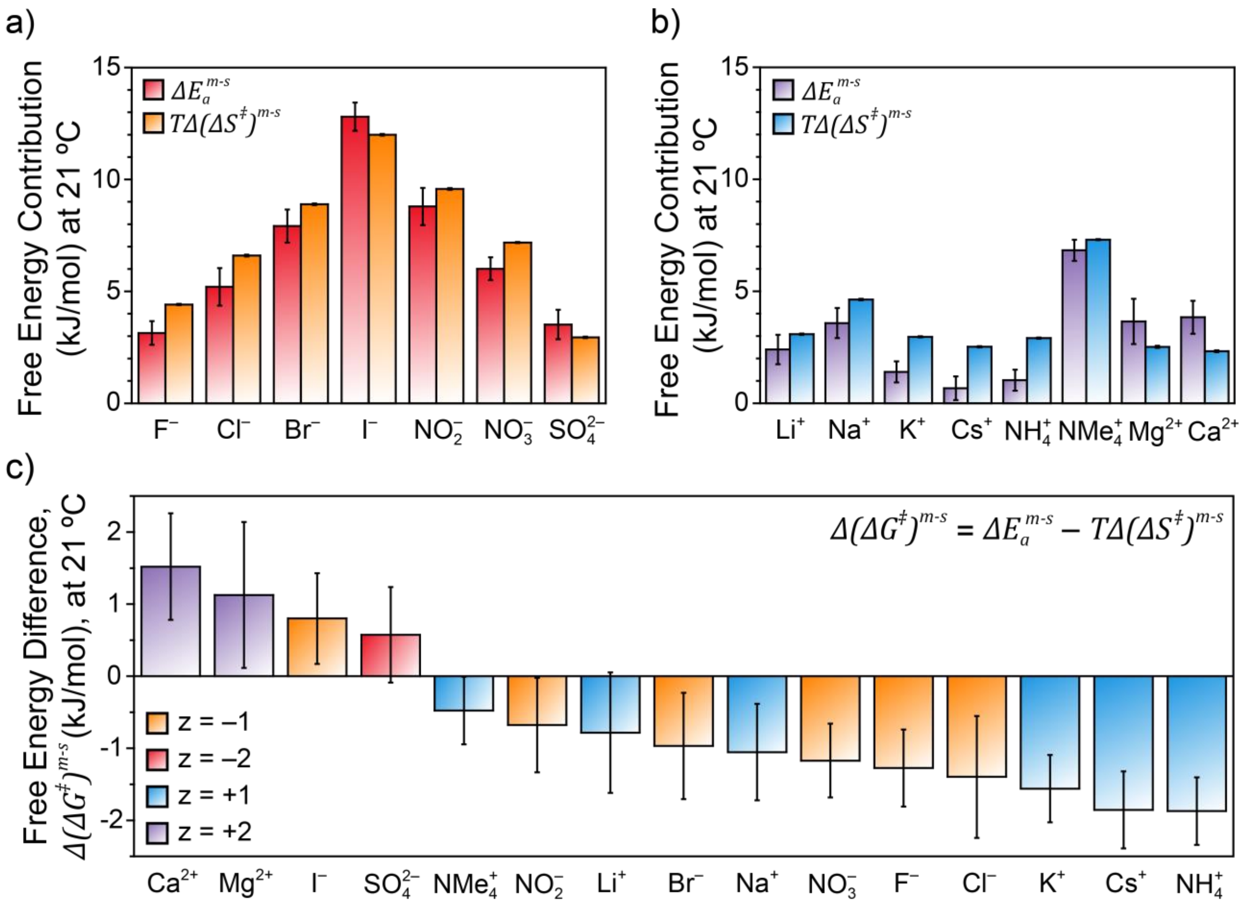

Figure S19: Room temperature free energy contributions. The difference in enthalpic free energy contributions (ΔE_a^{m-s}) compared with the difference in entropic free energy contributions ($\Delta(\Delta S^{\ddagger})^{m-s}$) between ions in the membrane and ions at infinite dilution for a) AEMs and b) CEMs. c) The difference in free energy for ion transport ($\Delta(\Delta G^{\ddagger})^{m-s}$) between ions in the IEMs and at infinite dilution. Entropic contributions and total free energies were evaluated at 21 ºC. Uncertainties were calculated from standard errors of the activation energies and entropies using standard propagation of uncertainty methods.

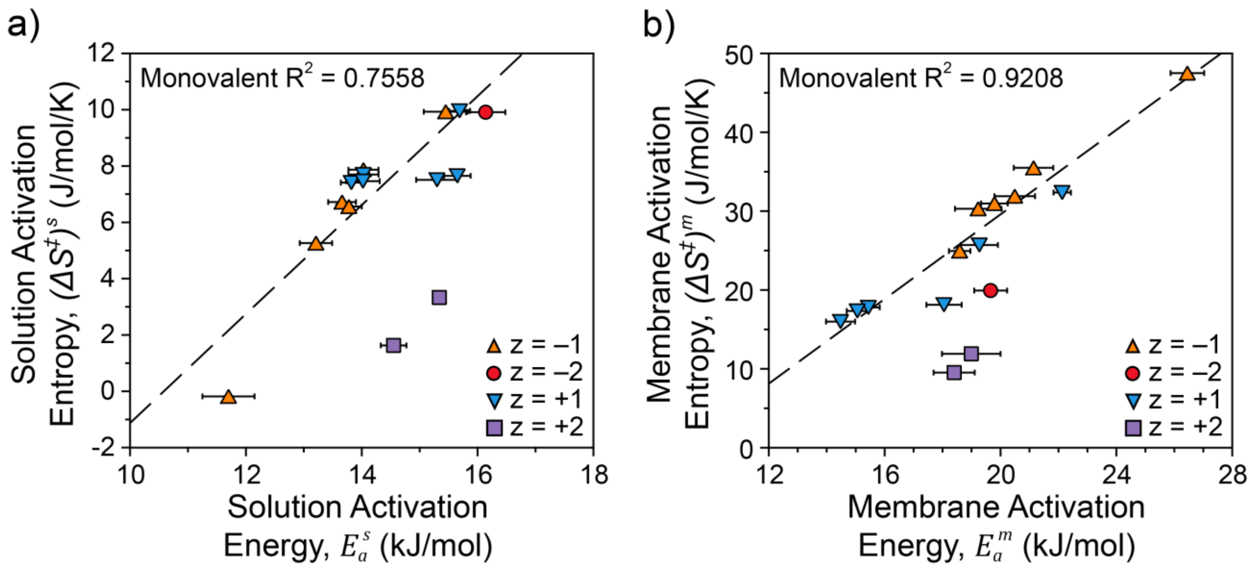

Figure S20: Linear free energy relationships. Linear free energy relationships for ion transport in a) dilute aqueous solution and b) membranes. The dashed lines represent linear fits of the monovalent ions. Divalent ion data are shown but not included in the linear regression. Uncertainties for the membrane data represent the standard error of the mean Arrhenius fit performed on four independently measured ionic conductivity data sets. Uncertainties for the aqueous solution data represent the standard error of the mean Arrhenius fit on the sampled data from the cubic fit of literature data.

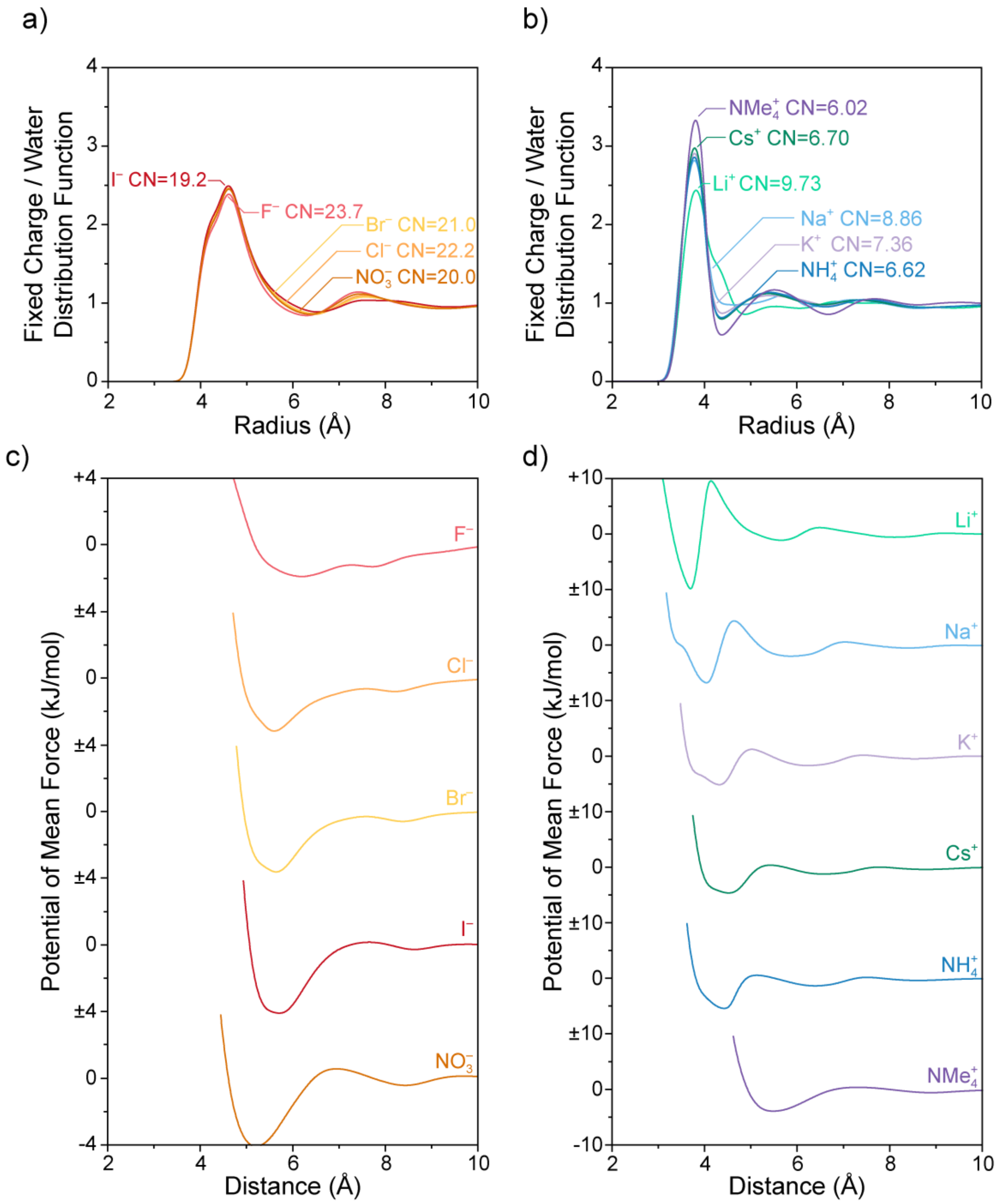

Figure S21: Fixed charge group/water interactions in the simulated IEMs. Radial distribution functions (a and b) and potential of mean force profiles (c and d) for the pair formed by the central atom of the fixed charge groups and the oxygen atom of TIP4P water molecules. AEMs are in panels a and c, CEMs in b and d. The first-shell coordination number (CN) is listed with the RDFs.

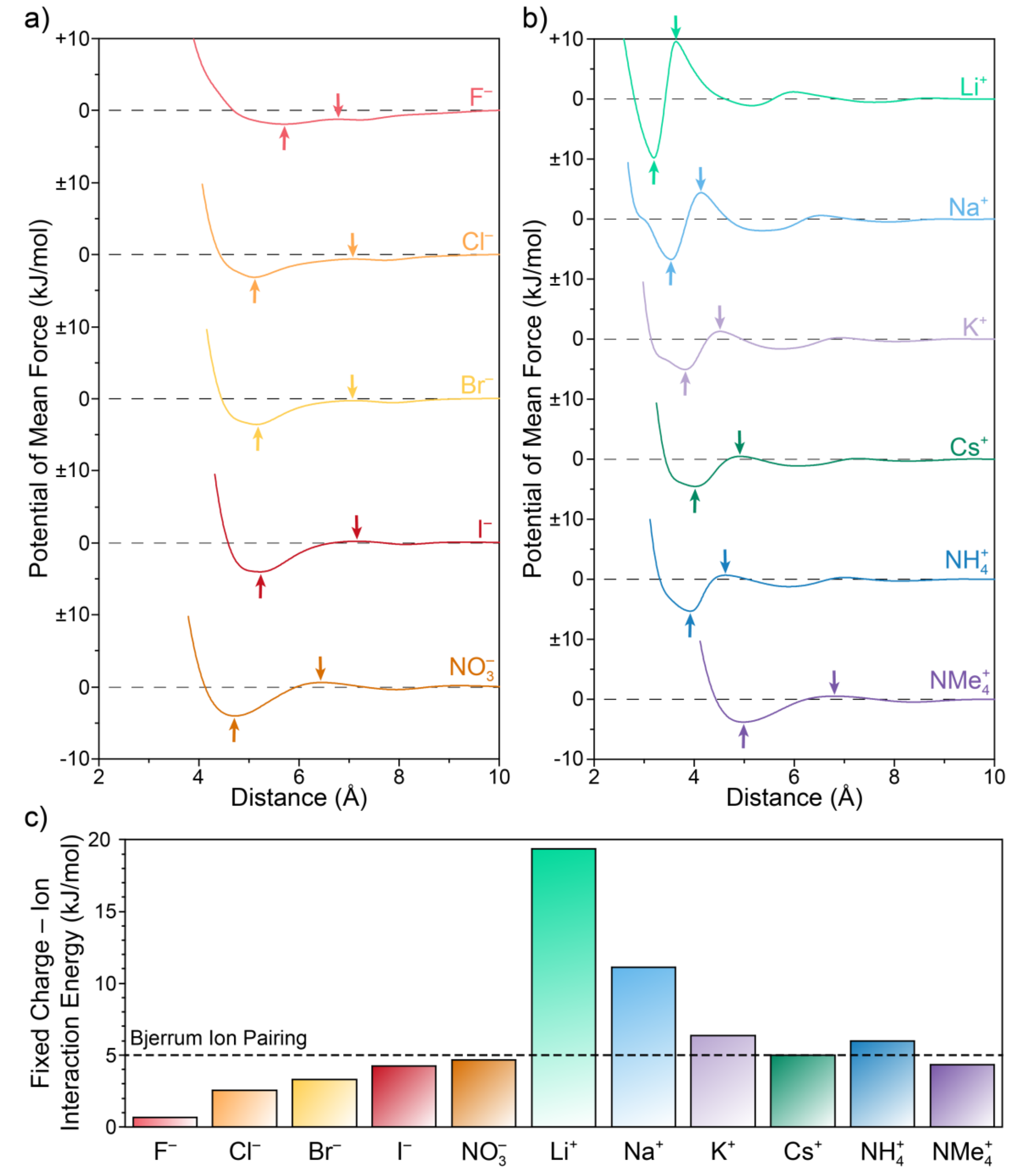

Figure S22: Potential of mean force for contact ion pairs. Potential of mean force (PMF) profiles for the interactions between the (a) anions and (b) cations with the fixed charge groups at 27 ºC as a function of separation distance. Extrema corresponding to the nearest population of mobile ions near fixed charge groups are marked with arrows. c) The ion interaction apparent in the PMF profiles compared to Bjerrum's criterion for ion pairing, $2k_BT \cong 5\ kJ/mol$.

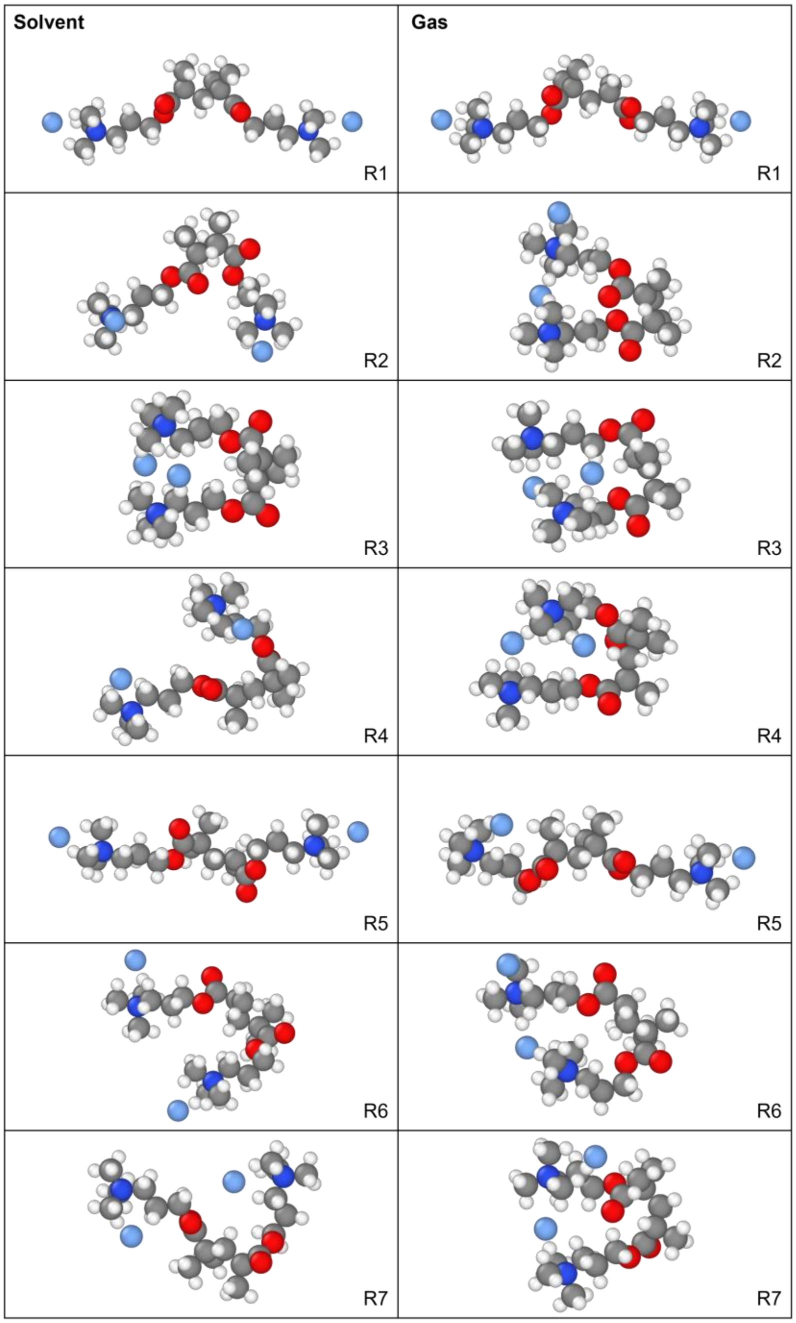

Figure S23: Anion conformations. Representative illustrations of seven final DFT optimized geometries for the MOETMA dimer with two F^- anions in the implicit solvent and gas-phase (dry) conditions. Color legend: O = red; N = dark blue; C = grey; H = beige; F = sky blue.

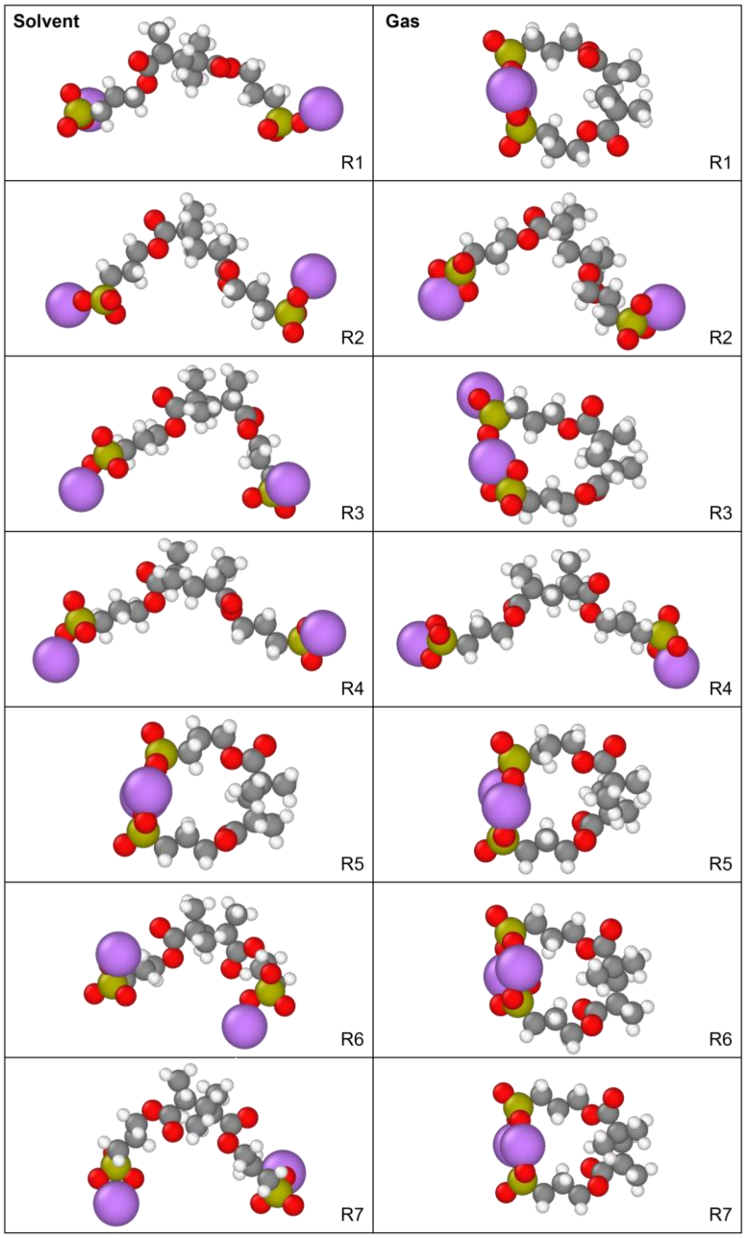

Figure S24: Cation conformations. Representative illustrations of seven final DFT optimized geometries for **t**he SPM dimer with two Li^+ cations in the implicit solvent and gas-phase (dry) conditions. Color legend: O = red; S = yellow; C = grey; H = beige; Li = purple.

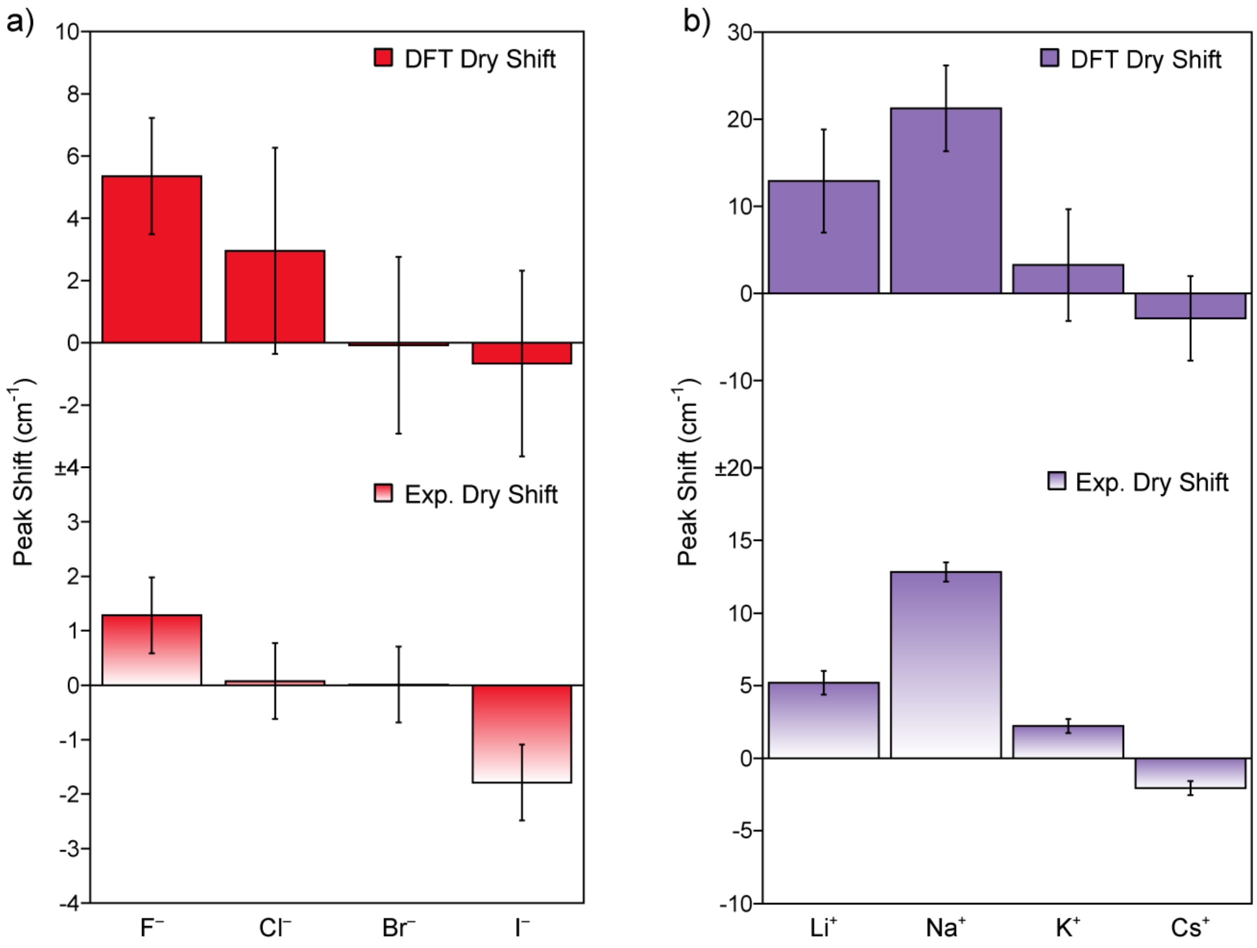

Figure S25: Simulated and observed peak shifts. DFT-predicted and experimentally measured Raman shifts for monoatomic, monovalent ions. a) The C–N stretch of $RNMe_3^+$ with F^-, Cl^-, Br^-, and I^- counter-ions in AEM. b) The symmetric S–O stretch of RSO_3^- with Li^+, Na^+, K^+, and Cs^+, counter-ions in the CEM. The centerline denotes the average peak location for hydrated systems, and the shift represents the difference incurred by drying the system. Experimental uncertainties reflect bootstrapped fits from Gaussian deconvolution of a single sample, while DFT uncertainties capture variability across seven independent initial geometries.

Note: The direction and magnitude of these peak shifts depend on the strength of interaction and the mass of the interacting species, rationalizing the counter-ion dependent energy difference in

contact ion pair formation (*100*, *172*). It is helpful to talk through examples for large and small counter-ions. For smaller bare ions, strong electrostatic interactions stiffen the spring constant of the fixed charge group bond to a greater extent than pairing affects the mass of the vibrating atoms, leading us to expect larger vibrational energies upon pairing. Conversely, for larger bare ions, the increased mass and reduced electrostatic interactions dampen the bond vibrations, leading us to expect lower vibrational energies upon pairing. In contrast to these ion-specific energetics of ion pairs, unpaired fixed charge groups in hydrated IEMs coordinate water similarly across counter-ion forms. As a result, the vibrational modes of unpaired fixed charge groups in AEMs and CEMs should exhibit vibration energies independent of counter-ion form (*100*, *172*). Li^+ form CEMs are a notable exception to these basic trends; however, the DFT conformations indicate a strong likelihood of bidentate coupling between multiple fixed charge groups. These clustered ion pairs likely are the source of this anomalous behavior.

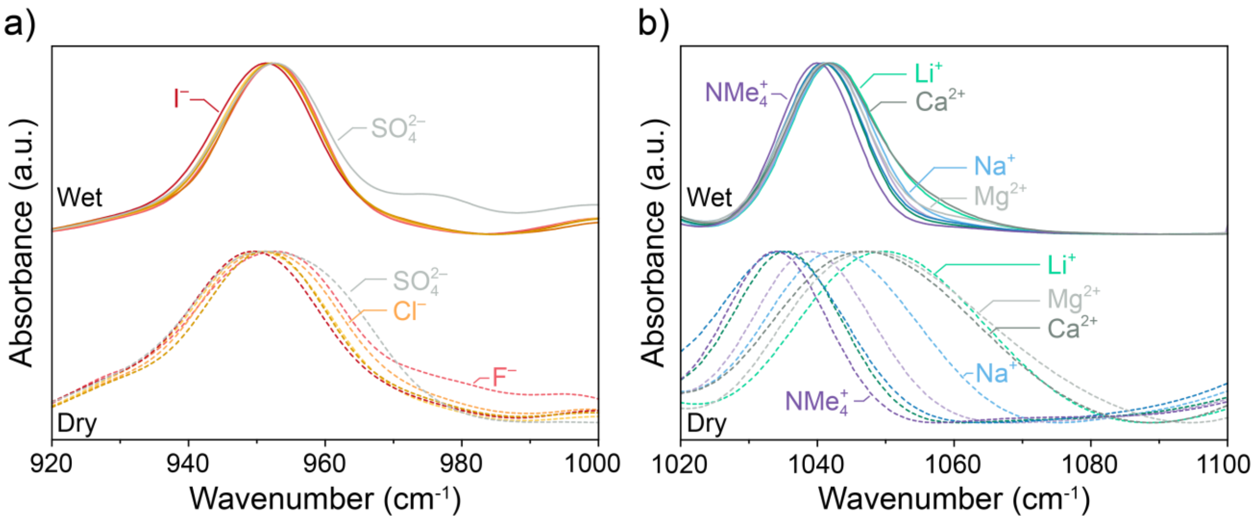

Figure S26: FTIR spectra of fixed charge group vibrations in hydrated and dried membranes. a) The C-N stretch of $RNMe_3^+$ in hydrated and dried AEMs. b) The S-O symmetric stretch of RSO_3^- in hydrated and dried CEMs. Hydrated spectra are shown as solid lines, while dried spectra are shown as dashed lines. Notable counter-ion spectra were labeled, while overlapping results were left unlabeled for clarity.

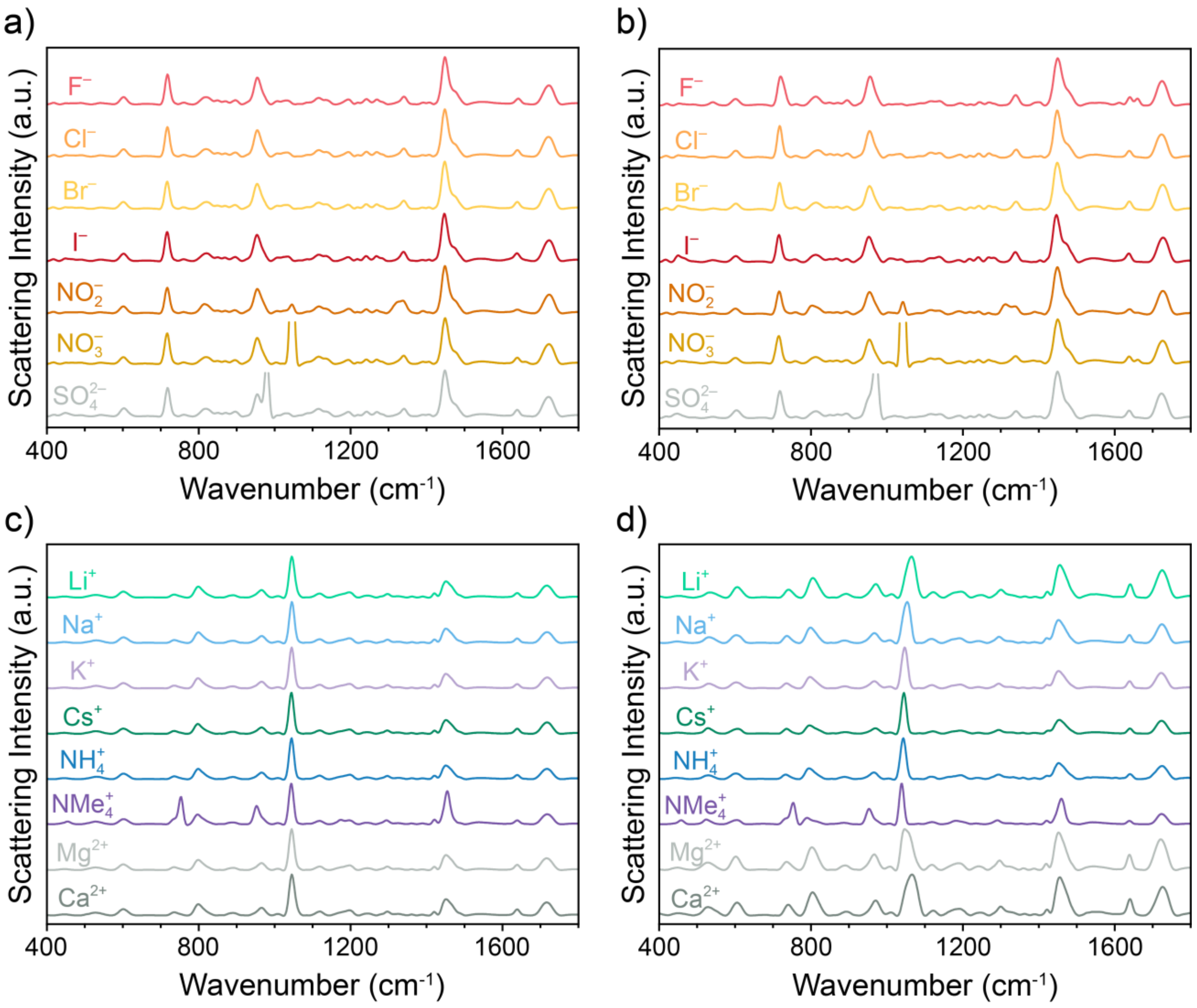

Figure S27: Raman spectra. The full Raman spectra collected for AEMs (a-b) and CEMs (c-d) in the hydrated (a, c) and dried (b, d) state. Signals for NO_3^- and SO_4^{2-} counter-ions in the AEMs were much stronger than the polymer backbone, so these peaks were truncated for clarity.

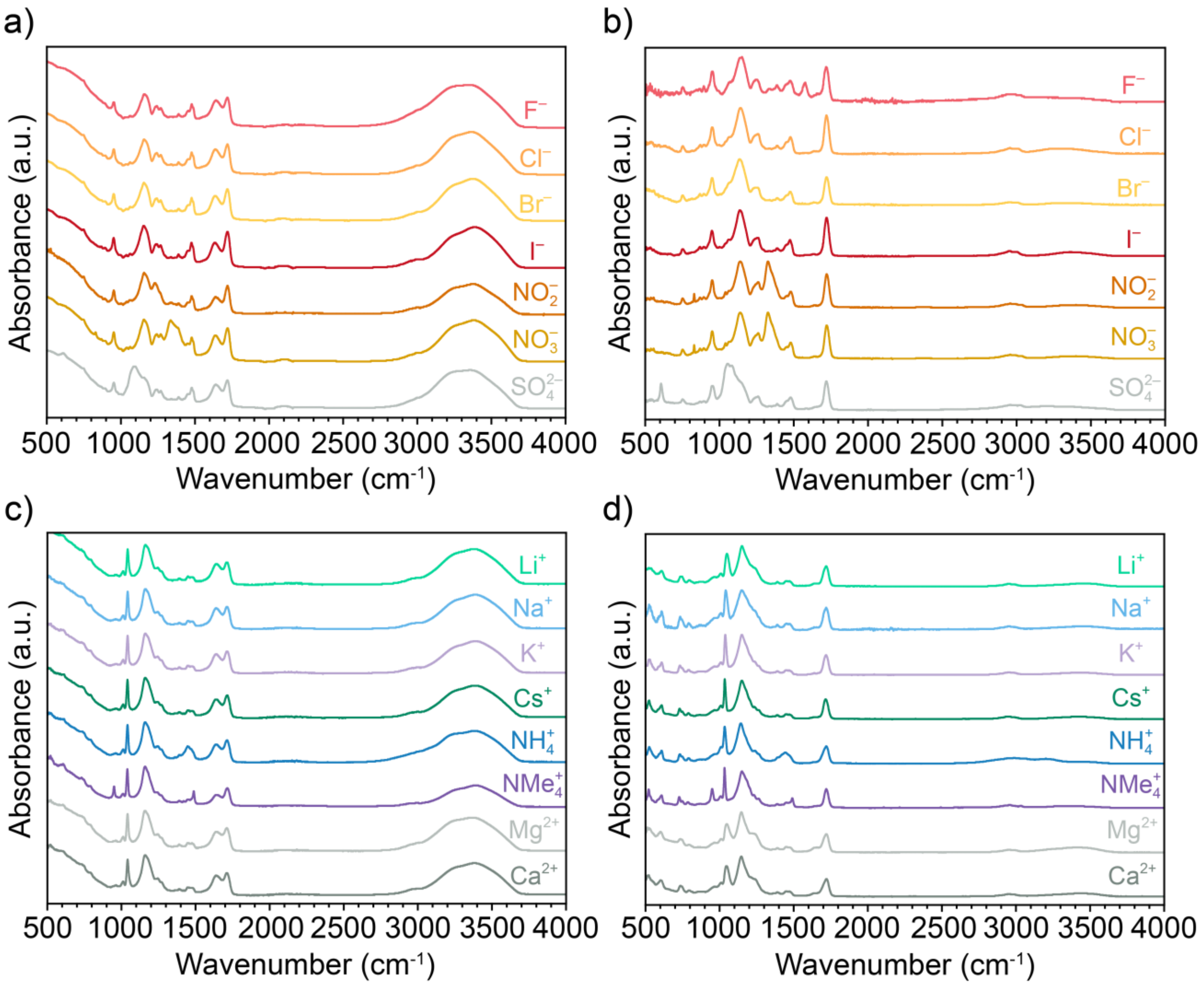

Figure S28: FTIR spectra of membranes. The full FTIR spectra collected for AEMs (a-b) and CEMs (c-d) in the hydrated (a, c) and dried (b, d) state.

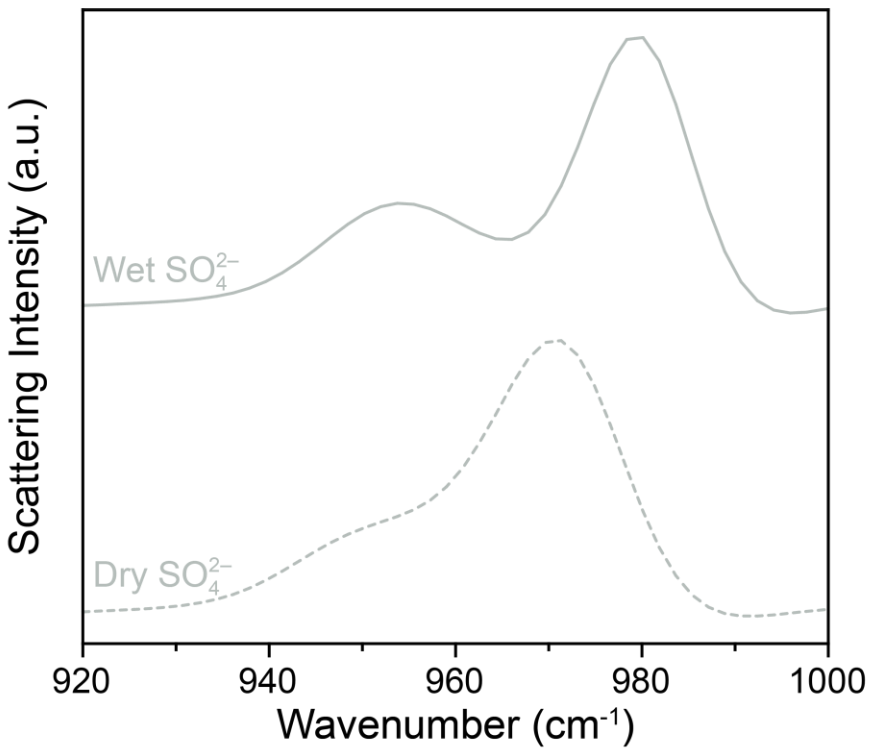

Figure S29: Sulfate Raman pairing peaks. The $RNMe_3^+$ Raman signal of AEMs in the SO_4^{2-} counter-ion form. The hydrated spectrum is shown as a solid line, while the dried spectrum is shown as a dashed line. The S-O vibration (970 – 980 cm^{-1}) dominates the C-N vibration (~954 cm^{-1}), complicating the analysis of ion pairing.

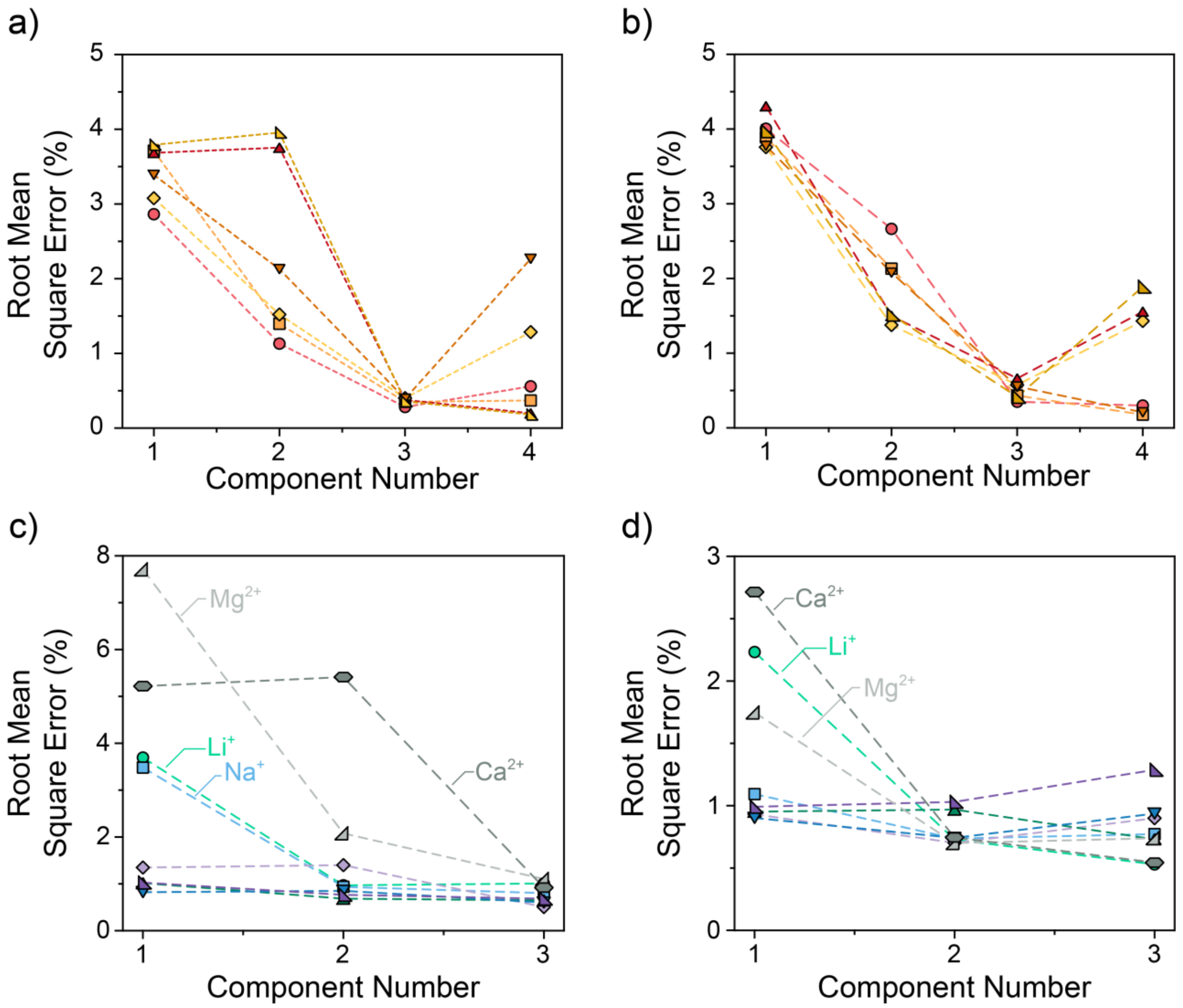

Figure S30: Deconvolution component plots. The root mean square error of Gaussian deconvolutions performed on Raman signals associated with the fixed charge groups for increasing numbers of peaks. Results are for AEMs (a-b) and CEMs (c-d) in the hydrated (a, c) and dried (b, d) state. Notable cation trends were labeled, while overlapping results were left unlabeled for clarity.

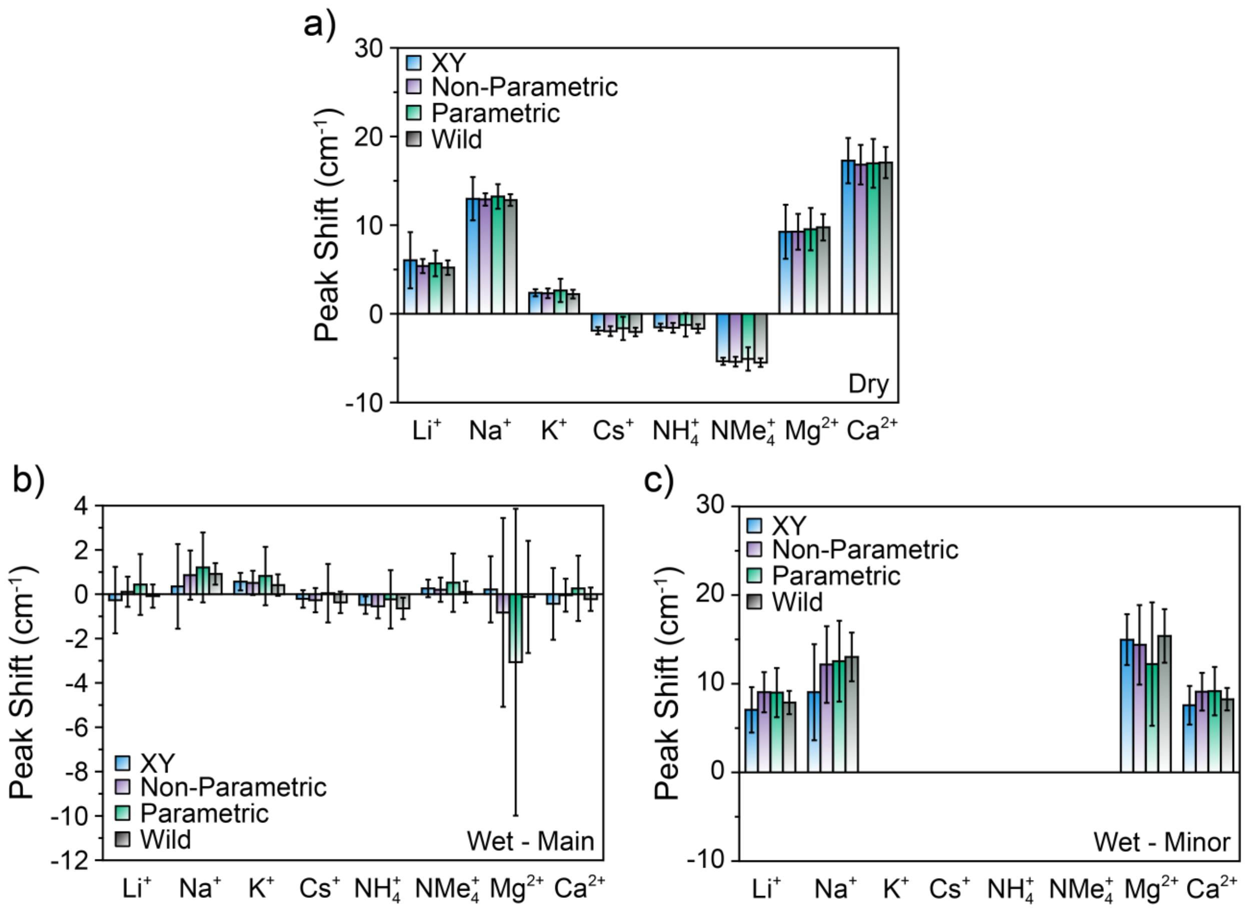

Figure S31: Bootlegging results for Gaussian deconvolutions. A comparison between the mean and standard deviation of Raman peak deconvolutions resulting from four Bootlegging algorithms, as discussed in Section S2.1.1. Representative results are shown for a) dry CEMs, b) the primary peak of hydrated CEMs, and c) the secondary peak of hydrated CEMs.

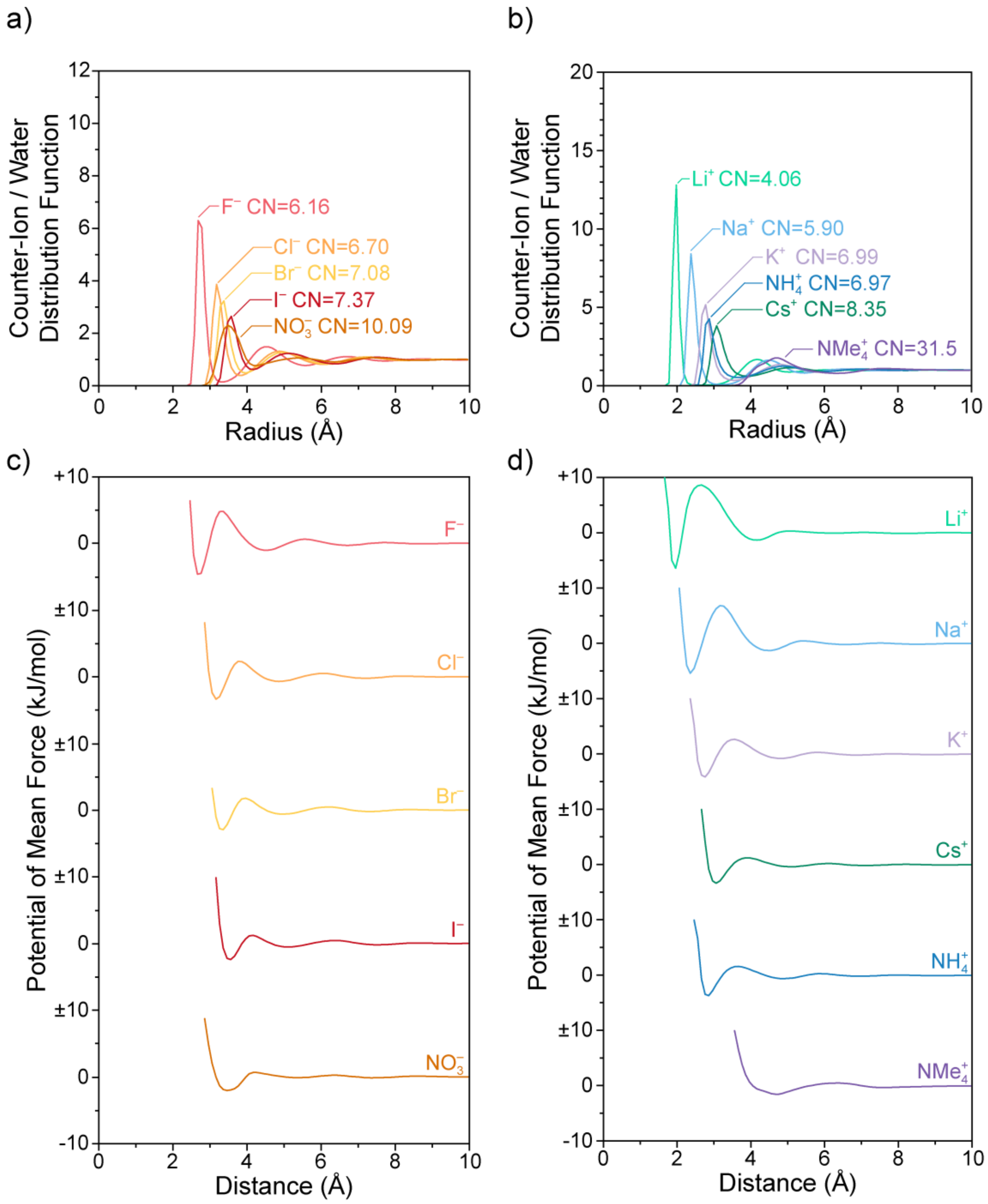

Figure S32: Counter-ion/water interactions simulated at infinite dilution. Radial distribution functions (a and b) and potential of mean force profiles (c and d) for the pair formed by the central atom of the counter-ion and the oxygen atom of TIP4P water molecules. Anions are in panels a and c, cations in b and d. The first-shell coordination number (CN) is listed with the RDFs.

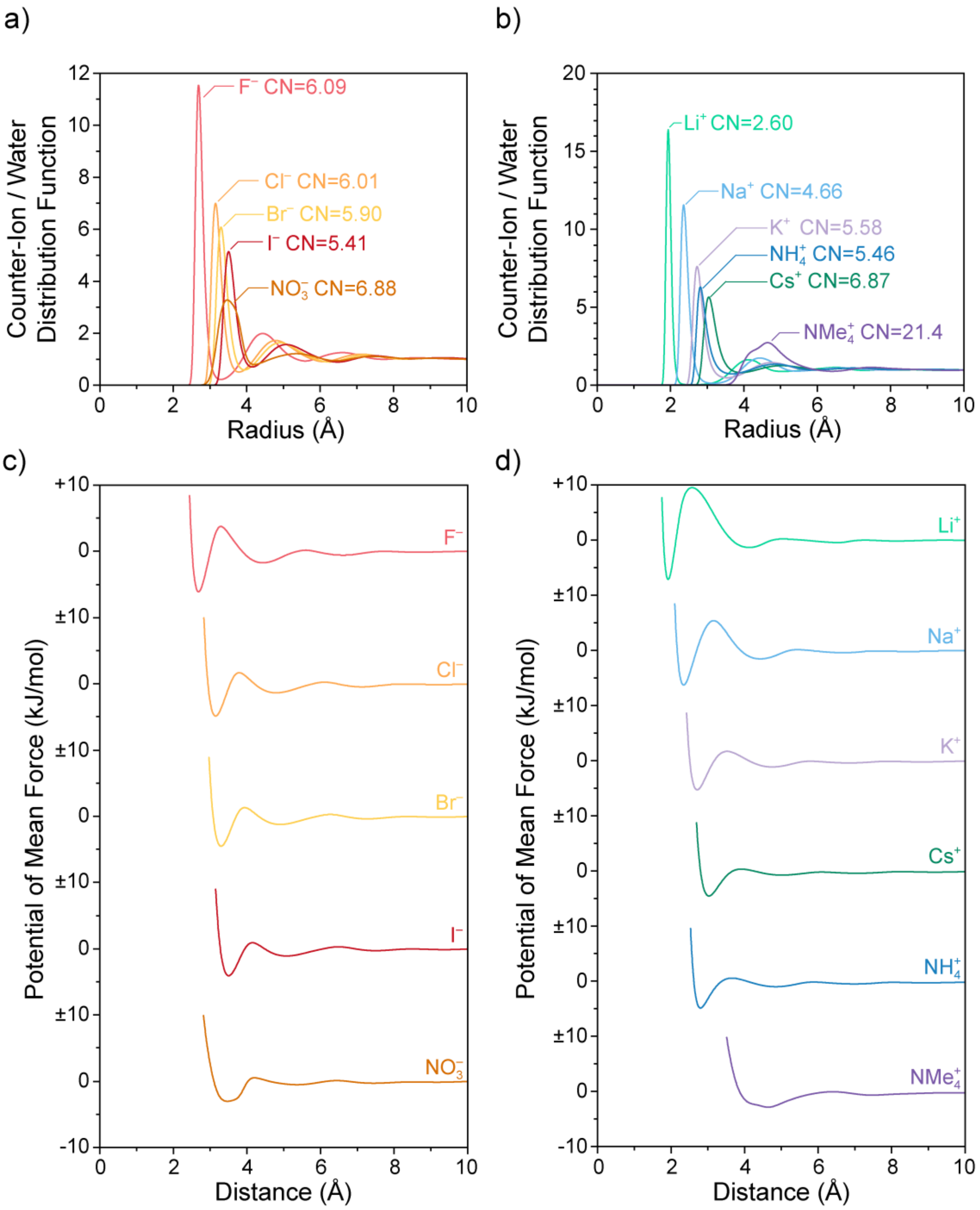

Figure S33: Counter-ion/water interactions in the simulated IEMs. Radial distribution functions (a and b) and potential of mean force profiles (c and d) for the pair formed by the central atom of the counter-ion and the oxygen atom of TIP4P water molecules in the membranes. AEMs are in panels a and c, CEMs in b and d. The first-shell coordination number (CN) is listed with the RDFs.

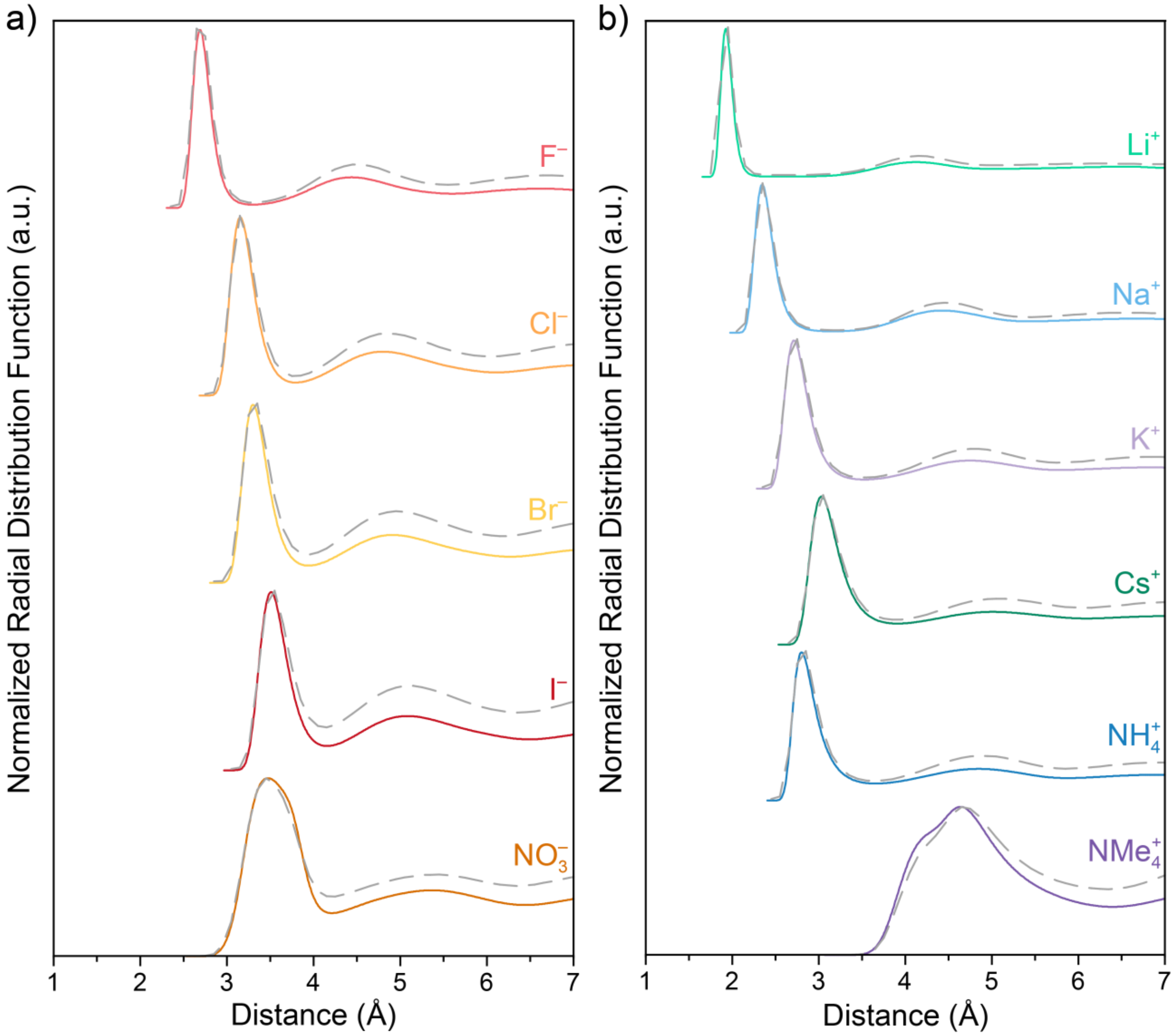

Figure S34: Overlay of ion/water distribution functions. Radial distribution functions for the pair formed by the central atom of the counter-ion and the oxygen atom of TIP4P water molecules both in the membranes (colored solid lines) and at infinite dilution (gray dashed lines) for a) anions and b) cations. Distribution functions were normalized by the maximum local density to facilitate a comparison of distances between the two profiles shown for each ion.

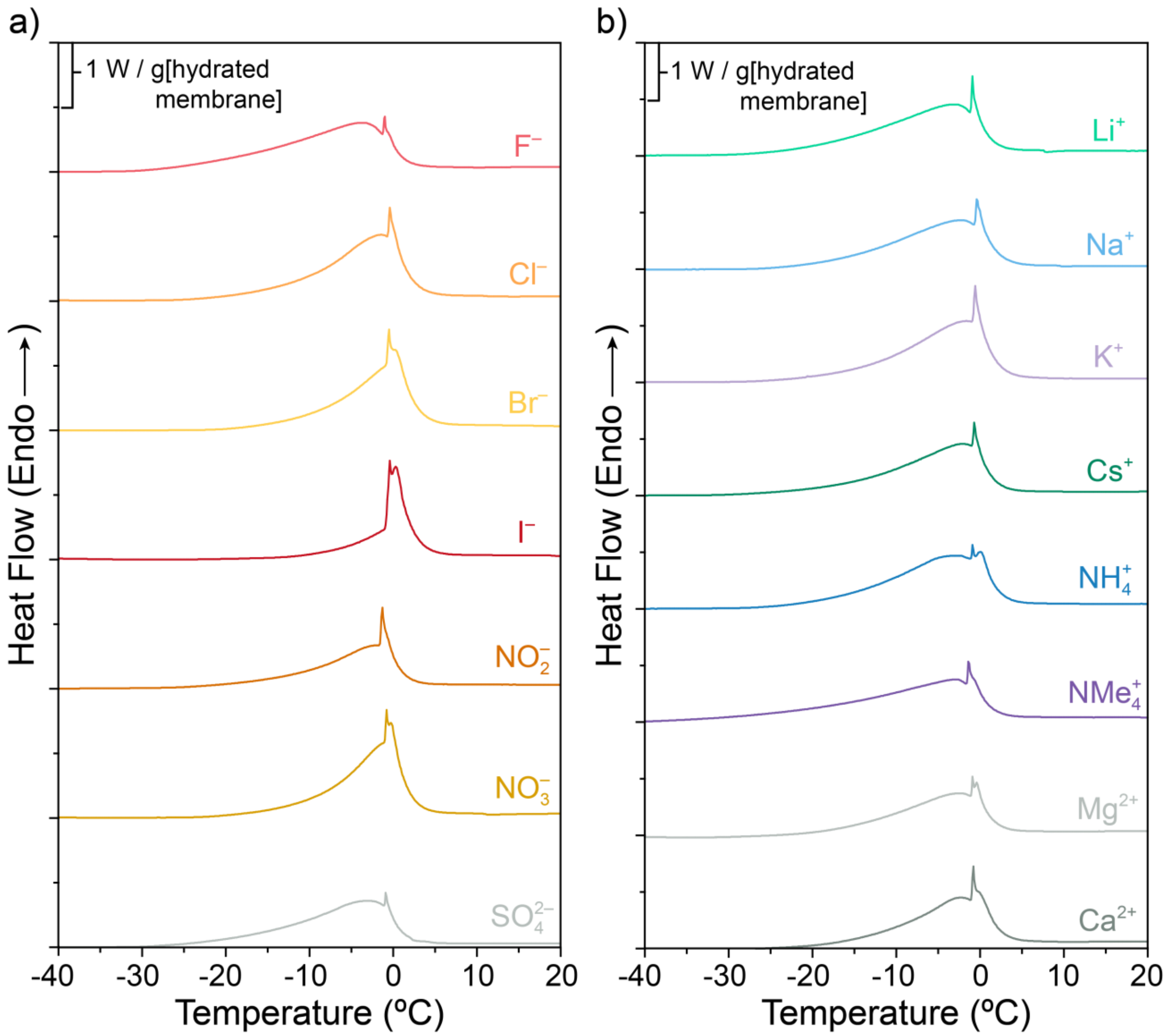

Figure S35: Differential scanning calorimetry thermograms. Representative thermograms of the water melting behavior for a) AEMs and b) CEMs.

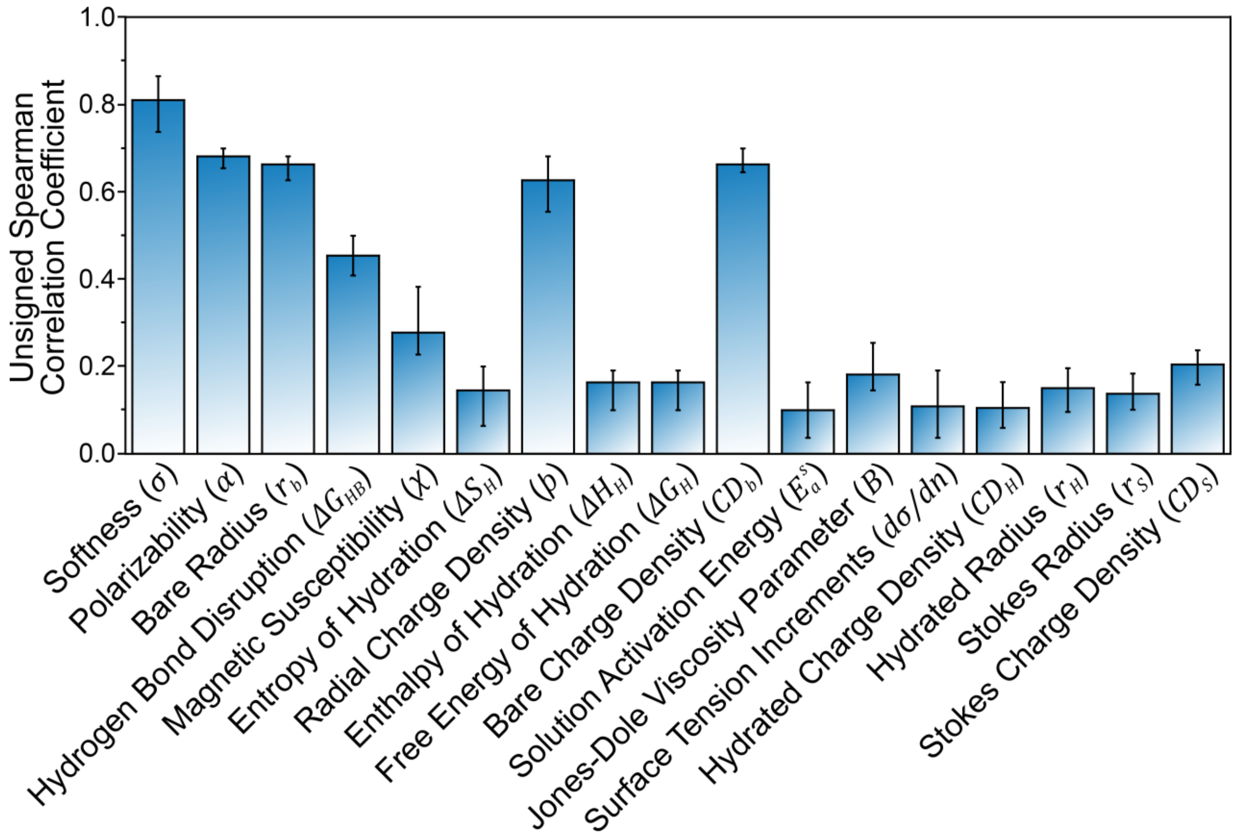

Figure S36: Spearman correlation analysis. The magnitude of Spearman correlation coefficients from a pairwise analysis of the activation energy difference, ΔE_a^{m-s}, with each ion property considered in this study (Table S15). The results are presented in descending order of the Pearson correlation coefficients shown in main body Fig. 8A for ease of comparison. Error bars display the 99% confidence interval estimated by a 10,000-point *Parametric* bootstrapping analysis (see Sections S2.6 and S2.8).

Note: Pearson coefficients quantify linear association and therefore may be modestly influenced by unequal predictor leverage (when one ion property varies more than another). Meanwhile, Spearman coefficients depend only on rank ordering and are insensitive to differences in numerical scale or spread. Comparing Fig. 8A with Figure S36, both correlation metrics produce the same qualitative ranking of predictors, indicating that the conclusions drawn from the Pearson analysis

are stable with respect to leverage and monotonic-relationship considerations. Importantly, the ion softness remains the best predictor for ΔE_a^{m-s}. Interestingly, the Spearman coefficients are enhanced for charge-density-based descriptors. This likely reflects mild non-linearities in how charge density relates to the activation energy, which are more faithfully captured by monotonic rank relationships than by purely linear fits.

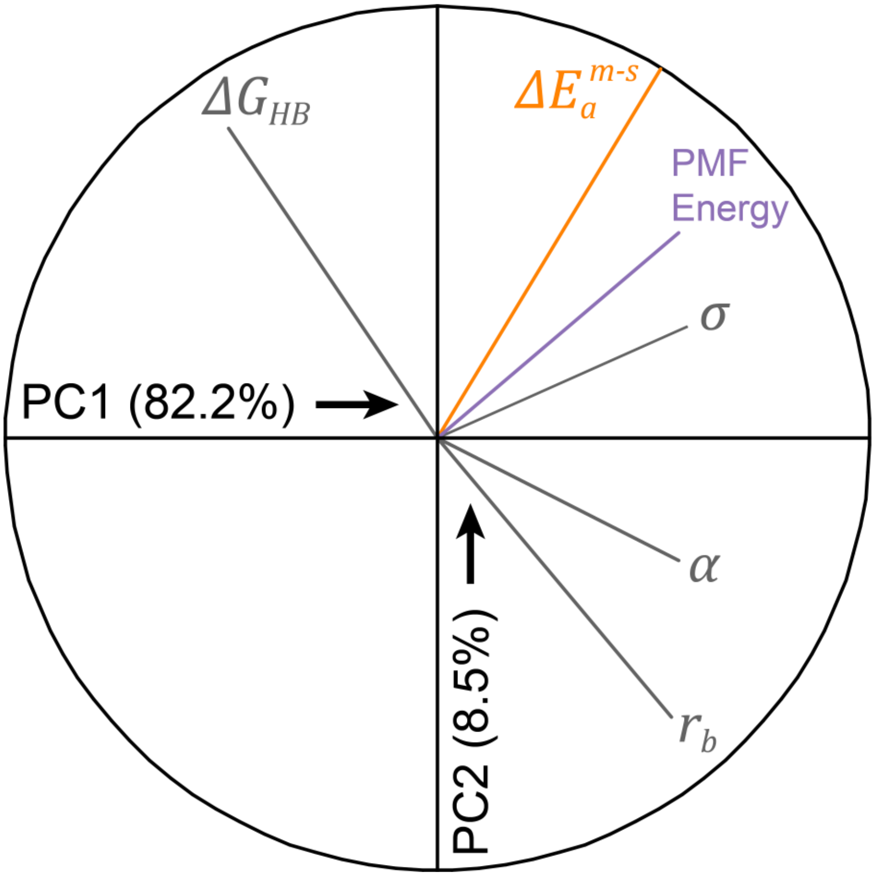

Figure S37: Principal component analysis. Biplot of significant ion properties correlated with the activation energy difference, ΔE_a^{m-s}, and the simulated ion interaction energy for solvent-mediated ion pairs extracted from the potentials of mean force (PMF). The experimental and simulated energies are shaded orange and purple, respectively. The properties include the ion softness, σ, the ion polarizability, α, the bare ionic radius, r_b, and the hydrogen bond disruption energy, ΔG_{HB}.

Note: Principal component analysis (PCA) is a powerful method of isolating the primary modes of variation within complex data sets by grouping covaried variables into principal components (PCs) that efficiently capture trends present in the data (*164*). Thus, PCA is an excellent tool to identify appropriate features for quantifying a phenomenon when many similar features are

available, as is the case for this array of ion properties. PCA helps to visualize complex information in a variety of ways, but for this analysis, the biplot shown in Figure S37 is the most helpful. Feature contributions to the most important PCs are plotted as vectors normalized in magnitude by the total variance captured by those PCs (*164*). Long vectors with near-0° or near-180° angles of separation indicate features sharing essentially all modes of variation within the data set (i.e., the features are strongly correlated or anti-correlated). Orthogonal vectors separated by approximately 90° represent features that share minimal variance (i.e., the features are weakly correlated), and short vectors correspond to features that do not vary directly with either of the plotted principal components. This biplot demonstrates how some of the ion property correlations are more helpful to consider than others. It is evident that α, r_b, and ΔG_{HB}, which exhibit vectors nearly perpendicular to that of ΔE_a^{m-s}, account for different modes of variability within this subsection of data than ΔE_a^{m-s}. Conversely, the variance of σ aligns the most closely to ΔE_a^{m-s}, sharing similar modes of variance as the simulated energy barriers for solvent-mediated ion pairs.

S5 Supplementary Tables

Table S1: Membrane synthesis recipes. Reagents used to prepare a typical batch of pre-polymer solution for the AEM and CEM.

IEM	Charged Monomer	Cross-Linker	Solvent	Initiator
AEM	9.38 g Monomer Solution (7.04 g MOETMA-Cl^-)	5.64 g GDMA	9.38 g Monomer Solution (2.35 g Water)<br>5.71 g Additional Water<br>1.04 g 1-propanol	150 mg V-50
CEM	10.00 g SPM-K^+	6.53 g GDMA	10.00 g Water	165 mg V-50

Table S2: Gravimetric membrane structural properties. The ion-exchange capacity (IEC), water uptake (WU), and dry polymer density (ρ_p) for each counter-ion form of IEMs. The fraction of theoretical IEC is included in parentheses to facilitate comparison between the counter-ion forms. Reported uncertainties represent the standard deviation of measurements made on at least five independent samples.

Counter-Ion Form	IEC (meq / g[polymer])	WU (g[water] / g[polymer])	ρ_p (g/cm^3)
F^-	2.546 ± 0.055 (90.8 ± 1.9 %)	1.117 ± 0.051	1.232 ± 0.006
Cl^-	2.460 ± 0.064 (91.8 ± 2.4 %)	0.883 ± 0.013	1.243 ± 0.002
Br^-	2.173 ± 0.056 (90.7 ± 2.3 %)	0.717 ± 0.004	1.356 ± 0.004
I^-	1.972 ± 0.049 (91.6 ± 2.3 %)	0.519 ± 0.005	1.453 ± 0.006
NO_2^-	2.286 ± 0.030 (87.7 ± 1.2 %)	0.800 ± 0.011	1.263 ± 0.005
NO_3^-	2.230 ± 0.014 (89.1 ± 0.5 %)	0.723 ± 0.005	1.288 ± 0.010
SO_4^{2-}	2.230 ± 0.035 (86.0 ± 1.4 %)	0.888 ± 0.004	1.309 ± 0.011
Li^+	2.371 ± 0.113 (90.0 ± 4.3 %)	0.954 ± 0.014	1.399 ± 0.013
Na^+	2.327 ± 0.080 (92.0 ± 3.1 %)	0.892 ± 0.008	1.417 ± 0.008
K^+	2.230 ± 0.132 (91.8 ± 5.4 %)	0.813 ± 0.009	1.442 ± 0.008
Cs^+	1.834 ± 0.028 (92.7 ± 1.4 %)	0.644 ± 0.001	1.701 ± 0.006
NH_4^+	2.852 ± 0.095 (111.4 ± 3.7 %)	0.866 ± 0.008	1.350 ± 0.009
NMe_4^+	2.342 ± 0.066 (104.6 ± 3.0 %)	0.703 ± 0.003	1.257 ± 0.005
Mg^{2+}	2.537 ± 0.036 (97.6 ± 1.4 %)	0.806 ± 0.016	1.427 ± 0.005
Ca^{2+}	2.357 ± 0.089 (82.5 ± 3.5 %)	0.790 ± 0.011	1.428 ± 0.007

Table S3: Volumetric membrane structural properties. The water volume fraction (ϕ_w), fixed charge density ($C_A^{m,t}$), fixed charge concentration ($C_A^{m,w}$), maximum charge density (C_A^{max}), and membrane hydration number (λ) for each counter-ion form of IEMs. Reported uncertainties represent the standard deviation of measurements made on at least five independent samples.

Counter-Ion Form	ϕ_w (L[water] / L [membrane])	$C_A^{m,t}$ (mol / L [membrane])	$C_A^{m,w}$ (mol / L[water])	C_A^{max} (mol / L[dry polymer])	λ (mol[water] / mol[charge])
F^-	0.611 ± 0.027	1.392 ± 0.093	2.279 ± 0.115	3.137 ± 0.069	24.4 ± 1.2
Cl^-	0.552 ± 0.026	1.537 ± 0.086	2.785 ± 0.083	3.057 ± 0.079	19.9 ± 0.6
Br^-	0.500 ± 0.015	1.514 ± 0.061	3.029 ± 0.079	2.947 ± 0.076	18.3 ± 0.5
I^-	0.445 ± 0.007	1.688 ± 0.052	3.796 ± 0.100	2.865 ± 0.072	14.6 ± 0.4
NO_2^-	0.525 ± 0.017	1.498 ± 0.055	2.856 ± 0.055	2.887 ± 0.040	19.4 ± 0.4
NO_3^-	0.489 ± 0.018	1.508 ± 0.058	3.083 ± 0.028	2.873 ± 0.028	18.0 ± 0.2
SO_4^{2-}	0.566 ± 0.019	1.419 ± 0.054	2.509 ± 0.041	2.919 ± 0.052	22.1 ± 0.4
Li^+	0.605 ± 0.020	1.504 ± 0.091	2.486 ± 0.124	3.318 ± 0.162	22.3 ± 1.1
Na^+	0.613 ± 0.021	1.599 ± 0.080	2.608 ± 0.093	3.297 ± 0.114	21.3 ± 0.8
K^+	0.568 ± 0.019	1.558 ± 0.107	2.742 ± 0.165	3.217 ± 0.191	20.3 ± 1.2
Cs^+	0.540 ± 0.006	1.537 ± 0.030	2.849 ± 0.044	3.121 ± 0.049	19.5 ± 0.3
NH_4^+	0.545 ± 0.012	1.796 ± 0.073	3.294 ± 0.114	3.849 ± 0.131	16.9 ± 0.6
NMe_4^+	0.483 ± 0.009	1.607 ± 0.054	3.329 ± 0.095	2.943 ± 0.084	16.7 ± 0.5
Mg^{2+}	0.567 ± 0.021	1.785 ± 0.078	3.146 ± 0.076	3.620 ± 0.054	17.7 ± 0.4
Ca^{2+}	0.567 ± 0.016	1.691 ± 0.083	2.984 ± 0.119	3.366 ± 0.128	18.6 ± 0.7

Table S4: Polymerization simulation conditions. The size and constituent molecules of the pre-polymer solution domains used to prepare the AEM and CEM *in silico*. The densities of the simulated and experimental pre-polymer solutions are provided for comparison.

Parameter	AEM	CEM
Number of GDMAs	2,100	2,100
Number initiated	47	52
Number of MOETMAs	2,900	---
Number initiated	33	---
Number of SPMs	---	2,900
Number initiated	---	36
Number of waters	38,500	40,000
Number of atoms	328,500	299,700
Domain side length (Å)	143.1	138.4
Simulated ρ_{pp} (g/cm^3)	1.057	1.208
Experimental ρ_{pp} (g/cm^3)	1.069 ± 0.031	1.193 ± 0.053

Table S5: Network incorporation properties. Post-polymerization properties of the simulated AEM and CEM, including the total number of unreacted methacrylate groups, the number of unreacted methacrylate groups corresponding to charged monomers (MOETMA and SPM), and the number of unreacted methacrylate groups corresponding to GDMA cross-linkers. The gel fraction (GF) and as-synthesized IEC (the Cl^- form for the AEM and the K^+ form for the CEM) are compared with the corresponding experimental values.

Properties	AEM	CEM
Number of unreacted methacrylate groups	265	199
Number of unreacted charged monomers	209	163
Number of unreacted GDMA methacrylate groups	56	36
Simulated GF (%)	96.0	95.9
Experimental GF (%)	96.0 ± 1.5	96.1 ± 1.2
Simulated IEC (meq / g[dry polymer])	2.59	2.39
Experimental IEC (meq / g[dry polymer])	2.460 ± 0.064	2.230 ± 0.132

Table S6: AEM conversion and hydration properties. Experimentally determined water uptakes and hydrated densities are reproduced alongside the number of water molecules added to the AEM simulations to match the experimental water uptake. Hydrated membrane densities (ρ_m) collected after the simulation equilibrated with the new ions and waters are compared to the corresponding experimental values.

Properties	F^-	Cl^-	Br^-	I^-	NO_3^-
Experimental WU (g[water] / g[dry polymer])	1.117 ± 0.051	0.883 ± 0.013	0.717 ± 0.004	0.519 ± 0.005	0.723 ± 0.005
Water Molecules Added Post-Polymerization	26109	12691	7944	-1149	6353
Simulated ρ_m (g/cm^3)	1.129	1.121	1.179	1.249	1.172
Experimental ρ_m (g/cm^3)	1.155 ± 0.032	1.205 ± 0.035	1.194 ± 0.038	1.298 ± 0.025	1.163 ± 0.040

Table S7: CEM conversion and hydration properties. Experimentally determined water uptakes and hydrated densities are reproduced alongside the number of water molecules added to the CEM simulations to match the experimental water uptake. Hydrated membrane densities (ρ_m) collected after the simulation equilibrated with the new ions and waters are compared to the corresponding experimental values.

Properties	Li^+	Na^+	K^+	Cs^+	NH_4^+	NMe_4^+
Experimental WU (g[water] / g[dry polymer])	0.954 ± 0.014	0.892 ± 0.008	0.813 ± 0.009	0.644 ± 0.001	0.866 ± 0.008	0.703 ± 0.003
Water Molecules Added Post-Polymerization	15997	14535	11696	10135	11871	8448
Simulated ρ_m (g/cm^3)	1.201	1.241	1.257	1.396	1.207	1.161
Experimental ρ_m (g/cm^3)	1.208 ± 0.011	1.226 ± 0.047	1.238 ± 0.042	1.375 ± 0.017	1.173 ± 0.027	1.167 ± 0.021

Table S8: Validation of equivalent circuits at high temperatures. In-plane ionic conductivities (κ) measured at elevated temperature using the modified Randles equivalent circuit fitting technique and the varied length technique. The varied length method was relied upon at high temperatures until the two methods agreed, then all lower temperatures were collected automatically using the equivalent circuit technique.

Counter-Ion Form	Temperature (ºC)	κ (mS/cm) Equivalent Circuit	κ (mS/cm) Varied Length
F^-	60	53.12 ± 2.94	52.56 ± 0.71
Cl^-	60	55.88 ± 4.67	54.71 ± 0.50
Br^-	60	44.16 ± 2.05	43.54 ± 0.44
I^-	60	25.76 ± 2.34	24.70 ± 0.84
NO_2^-	60	45.72 ± 6.85	43.65 ± 4.79
NO_3^-	60	42.04 ± 1.31	41.95 ± 1.54
SO_4^{2-}	60	37.47 ± 1.05	34.73 ± 1.94
Li^+	60	30.35 ± 0.87	29.61 ± 1.49
Na^+	60	46.53 ± 2.06	46.47 ± 0.89
K^+	60	54.49 ± 5.83	62.57 ± 1.17
Cs^+	60	64.77 ± 4.41	60.54 ± 2.60
	60	---†	76.16 ± 2.57
NH_4^+	50	---†	61.18 ± 2.67
	40	55.43 ± 0.40	53.10 ± 0.69
NMe_4^+	60	21.92 ± 0.85	21.27 ± 1.47
Mg^{2+}	60	14.81 ± 0.83	15.04 ± 0.27
Ca^{2+}	60	16.97 ± 0.19	17.04 ± 0.45

†Equivalent circuit fitting did not converge.

Table S9: Membrane ionic conductivities. The ionic conductivity (mS/cm) of each counter-ion form IEM as a function of temperature. Uncertainties represent the standard deviation of measurements made on four independent samples.

Counter-Ion Form	10 ºC	20 ºC	30 ºC	40 ºC	50 ºC	60 ºC
F^-	15.96 ± 0.34	21.97 ± 0.52	28.06 ± 1.04	35.64 ± 1.74	44.11 ± 2.62	52.56 ± 0.71
Cl^-	15.87 ± 1.10	21.87 ± 1.97	29.96 ± 1.96	38.62 ± 2.21	47.84 ± 2.73	54.71 ± 0.50
Br^-	11.21 ± 0.41	16.35 ± 0.85	22.39 ± 1.11	28.55 ± 0.89	36.90 ± 1.08	43.54 ± 0.44
I^-	4.55 ± 0.15	7.84 ± 1.02	10.07 ± 0.25	14.18 ± 0.69	18.97 ± 1.16	24.70 ± 0.84
NO_2^-	12.31 ± 1.11	18.28 ± 2.08	23.67 ± 3.36	30.31 ± 3.92	36.98 ± 4.14	45.72 ± 6.85
NO_3^-	12.06 ± 0.24	16.61 ± 0.26	22.07 ± 0.60	28.44 ± 1.22	35.55 ± 1.69	41.95 ± 1.54
SO_4^{2-}	10.33 ± 0.44	14.35 ± 0.64	18.49 ± 1.05	23.65 ± 1.58	30.62 ± 0.61	34.73 ± 1.94
Li^+	9.60 ± 0.42	12.92 ± 0.45	16.90 ± 0.51	21.41 ± 0.76	26.04 ± 0.65	29.61 ± 1.49
Na^+	13.79 ± 0.45	19.13 ± 0.74	25.03 ± 0.93	32.24 ± 0.84	41.23 ± 2.23	46.47 ± 0.89
K^+	23.21 ± 1.01	30.60 ± 1.41	36.12 ± 1.71	45.89 ± 3.47	54.37 ± 4.58	62.57 ± 1.17
Cs^+	23.50 ± 1.64	30.99 ± 2.35	37.95 ± 2.70	45.64 ± 3.05	53.78 ± 4.11	60.54 ± 2.60
NH_4^+	28.36 ± 1.25	36.76 ± 2.54	44.20 ± 2.43	53.10 ± 0.69	61.18 ± 2.67	76.16 ± 2.57
NMe_4^+	5.32 ± 0.10	7.66 ± 0.17	10.13 ± 0.20	13.51 ± 0.43	17.54 ± 0.46	21.92 ± 0.85
Mg^{2+}	4.31 ± 0.32	5.87 ± 0.79	8.34 ± 0.45	10.76 ± 0.37	12.82 ± 0.17	15.04 ± 0.27
Ca^{2+}	5.38 ± 0.11	7.53 ± 0.25	9.33 ± 0.44	12.29 ± 0.42	15.28 ± 0.53	17.04 ± 0.45

Table S10: Cubic fits of limiting equivalent conductances of ions in aqueous solution. The limiting equivalent conductance of ions as a function of the temperature in Celsius (t).

Ion	Range (ºC)	λ_i^0 $(S \cdot cm^2/eq)$						
F^-	5 – 90	$0.32\times10^{-5}t^3$	+	$2.85\times10^{-3}t^2$	+	$1.06t$	+	26.67
Cl^-	0 – 100	$0.58\times10^{-5}t^3$	+	$3.43\times10^{-3}t^2$	+	$1.30t$	+	40.50
Br^-	0 – 90	$4.25\times10^{-5}t^3$	+	$-1.68\times10^{-3}t^2$	+	$1.42t$	+	41.58
I^-	0 – 65	$-0.42\times10^{-5}t^3$	+	$4.34\times10^{-3}t^2$	+	$1.26t$	+	42.26
NO_2^-	0 – 30	$-0.57\times10^{-5}t^3$	+	$-8.13\times10^{-3}t^2$	+	$1.34t$	+	44.00
NO_3^-	0 – 100	$-5.97\times10^{-5}t^3$	+	$10.18\times10^{-3}t^2$	+	$0.99t$	+	40.65
SO_4^{2-}	0 – 100	$-5.97\times10^{-5}t^3$	+	$13.12\times10^{-3}t^2$	+	$1.36t$	+	41.18
Li^+	0 – 90	$7.65\times10^{-5}t^3$	+	$-3.51\times10^{-3}t^2$	+	$0.84t$	+	18.34
Na^+	0 – 100	$-2.79\times10^{-5}t^3$	+	$8.45\times10^{-3}t^2$	+	$0.72t$	+	26.60
K^+	0 – 100	$0.57\times10^{-5}t^3$	+	$3.36\times10^{-3}t^2$	+	$1.28t$	+	39.70
Cs^+	0 – 90	$0.98\times10^{-5}t^3$	+	$4.45\times10^{-3}t^2$	+	$1.25t$	+	43.44
NH_4^+	0 – 100	$2.34\times10^{-5}t^3$	+	$1.00\times10^{-3}t^2$	+	$1.33t$	+	38.01
NMe_4^+	0 – 90	$2.34\times10^{-5}t^3$	+	$0.22\times10^{-3}t^2$	+	$0.88t$	+	21.03
Mg^{2+}	0 – 90	$5.66\times10^{-5}t^3$	+	$1.29\times10^{-3}t^2$	+	$0.88t$	+	27.29
Ca^{2+}	0 – 90	$13.39\times10^{-5}t^3$	+	$-8.36\times10^{-3}t^2$	+	$1.29t$	+	28.71
Pic^-	0 – 100	$4.25\times10^{-5}t^3$	+	$-1.68\times10^{-3}t^2$	+	$1.42t$	+	41.58

Table S11: Transport energetics in the membranes and in aqueous solution. The activation energy (E_a^j) and activation entropy ($(\Delta S^{\ddagger})^j$) for ions in the membranes ($j = m$) and in dilute aqueous solutions ($j = s$). Uncertainties for the membrane results represent the standard error of the mean for the linear fit performed on tortuosity-corrected equivalent ionic conductance data. Uncertainties for the solution results represent the standard error of the mean for the linear fit performed on sampled data points calculated using literature equivalent ionic conductance data.

Ion	E_a^m (kJ/mol)	$(\Delta S^{\ddagger})^m$ (J/mol/K)	E_a^s (kJ/mol)	$(\Delta S^{\ddagger})^s$ (J/mol/K)
F^-	18.59 ± 0.37	24.95 ± 0.06	15.45 ± 0.38	9.93 ± 0.07
Cl^-	19.23 ± 0.80	30.38 ± 0.14	14.03 ± 0.26	7.87 ± 0.05
Br^-	21.14 ± 0.68	35.53 ± 0.12	13.21 ± 0.28	5.26 ± 0.05
I^-	26.45 ± 0.58	47.35 ± 0.13	13.66 ± 0.24	6.72 ± 0.04
NO_2^-	20.49 ± 0.70	31.80 ± 0.13	11.70 ± 0.45	-0.18 ± 0.05
NO_3^-	19.79 ± 0.46	30.96 ± 0.08	13.78 ± 0.22	6.56 ± 0.04
SO_4^{2-}	19.66 ± 0.57	19.88 ± 0.10	16.14 ± 0.34	9.91 ± 0.06
Li^+	18.05 ± 0.61	18.12 ± 0.11	15.65 ± 0.23	7.65 ± 0.04
Na^+	19.27 ± 0.64	25.72 ± 0.11	15.69 ± 0.18	9.96 ± 0.03
K^+	15.44 ± 0.39	17.80 ± 0.07	14.03 ± 0.26	7.70 ± 0.05
Cs^+	14.48 ± 0.50	16.05 ± 0.09	13.82 ± 0.18	7.41 ± 0.03
NH_4^+	15.06 ± 0.37	17.32 ± 0.07	14.02 ± 0.29	7.46 ± 0.05
NMe_4^+	22.13 ± 0.30	32.35 ± 0.05	15.30 ± 0.36	7.51 ± 0.07
Mg^{2+}	18.99 ± 1.01	12.03 ± 0.16	15.34 ± 0.08	3.33 ± 0.01
Ca^{2+}	18.40 ± 0.71	9.53 ± 0.13	14.55 ± 0.22	1.63 ± 0.04

Table S12: Bootstrap sensitivity analysis of Arrhenius fitting parameters. The percent error in Arrhenius fitting parameters is calculated by 5000 point bootstraps of the linear fit of the Corrected Equivalent Ionic Conductance Arrhenius plot data (Figure S15). The parametric results resampled the data using the standard errors of the mean to estimate the effect of measurement uncertainty on fit parameters. The non-parametric results resampled and redistributed the residual of experimental data from the linear fit to estimate the effect of non-linearities on the fit parameters. The ratio between the summed slope and intercept uncertainties calculated by each method are shown in the final column. This approximately assess the importance of measurement uncertainty vs. linear deviations in the Arrhenius analysis.

Ion	Parametric Slope Uncertainty (%)	Parametric Intercept Uncertainty (%)	Non-Parametric Slope Uncertainty (%)	Non-Parametric Intercept Uncertainty (%)	Measurement to Non-Linearity Uncertainty Ratio
F^-	7.67	4.66	1.99	1.21	3.85
Cl^-	8.37	5.09	3.87	2.36	2.16
Br^-	5.11	3.20	3.30	2.08	1.54
I^-	2.89	2.02	3.89	2.72	0.74
NO_2^-	7.47	4.66	3.46	2.15	2.16
NO_3^-	6.53	3.97	2.44	1.48	2.68
SO_4^{2-}	5.95	3.74	2.93	1.84	2.04
Li^+	6.48	4.08	3.47	2.19	1.87
Na^+	5.98	3.75	3.32	2.08	1.80
K^+	8.18	4.46	2.69	1.47	3.04
Cs^+	4.84	2.50	3.78	1.98	1.27
NH_4^+	5.95	3.14	2.54	1.34	2.34
NMe_4^+	3.47	2.34	1.29	0.87	2.68
Mg^{2+}	6.70	4.61	4.53	3.13	1.48
Ca^{2+}	5.38	3.57	3.90	2.59	1.38

Note: Only I^- shows larger uncertainty from non-linearities than measurement (ratio = 0.74), suggesting that the effect of curvature in the Arrhenius fit plots is negligible in this work.

Table S13: Water coordination of ions in dilute aqueous solution. Ion-water coordination numbers for ions at infinite dilution (CN_{g-w}^{ID}) in our simulations are compared with accepted literature values compiled by Marcus (*18*). Literature values for thermodynamic hydration numbers of each ion at infinite dilution (HN_{g-w}^{ID}) are also included.

Ion	CN_{g-w}^{ID} (Our Simulation)	CN_{g-w}^{ID} (Literature Simulations)	CN_{g-w}^{ID} (Literature Scattering)	HN_{g-w}^{ID} (Literature Thermodynamics)
F^-	6.2	4.6	4.0 – 6.0	4.2
Cl^-	6.7	5.6	5.3 – 8.0	2.0
Br^-	7.1	---	4.2 – 6.5	1.3
I^-	7.4	8.0	4.2 – 9.6	0.9
NO_2^-	---	---	---	2.3
NO_3^-	10.1	12.8	3.0 – 4.3	1.6
SO_4^{2-}	---	11.3	7.6 – 9.6	6.4
Li^+	4.1	11.3	4.0 – 6.0	4.2
Na^+	5.9	---	4.0 – 8.0	3.3
K^+	7.0	4.2	6.0 – 8.0	2.3
Cs^+	8.3	5.4	6.0 – 8.0	1.7
NH_4^+	7.0	---	4.0 – 8.0	1.9
NMe_4^+	31.5	---	---	2.1
Mg^{2+}	---	6.0	6.0	6.0
Ca^{2+}	---	7.0	6.0 – 7.0	7.0

Table S14: States of water in the membranes. The water enthalpy of melting (ΔH_m) measured for each counter-ion form IEM alongside the calculated fraction of freezable water (f_f), freezable hydration number (λ_f), and non-freezable hydration number (λ_{nf}). Uncertainties represent the standard deviation calculated using standard propagation of uncertainty techniques from experimental data averaged over at least 5 samples.

Ion	ΔH_m^m (J / g[water])	f_f	λ_f	λ_{nf}
F^-	119.2 ± 2.8	0.358 ± 0.008	8.72 ± 0.49	15.65 ± 0.82
Cl^-	138.1 ± 5.3	0.415 ± 0.016	8.27 ± 0.40	11.68 ± 0.47
Br^-	140.8 ± 5.7	0.423 ± 0.017	7.76 ± 0.37	10.58 ± 0.42
I^-	104.2 ± 3.5	0.313 ± 0.010	4.58 ± 0.19	10.06 ± 0.31
NO_2^-	160.7 ± 4.1	0.483 ± 0.006	9.39 ± 0.21	10.06 ± 0.22
NO_3^-	149.2 ± 2.0	0.448 ± 0.006	8.07 ± 0.13	9.95 ± 0.14
SO_4^{2-}	119.7 ± 3.0	0.359 ± 0.009	7.96 ± 0.42	14.18 ± 0.31
Li^+	145.9 ± 2.8	0.438 ± 0.008	9.79 ± 0.52	12.56 ± 0.65
Na^+	146.8 ± 1.6	0.441 ± 0.005	9.39 ± 0.35	11.91 ± 0.43
K^+	168.1 ± 3.7	0.505 ± 0.011	10.23 ± 0.65	10.03 ± 0.64
Cs^+	167.2 ± 1.6	0.502 ± 0.005	9.79 ± 0.18	9.71 ± 0.18
NH_4^+	167.9 ± 4.1	0.504 ± 0.012	8.50 ± 0.36	8.36 ± 0.36
NMe_4^+	165.8 ± 3.4	0.498 ± 0.010	8.31 ± 0.29	8.38 ± 0.29
Mg^{2+}	122.6 ± 2.0	0.368 ± 0.006	6.50 ± 0.19	11.16 ± 0.29
Ca^{2+}	130.8 ± 3.1	0.393 ± 0.009	7.31 ± 0.34	11.31 ± 0.48

Table S15: Tabulated ion parameters. The enthalpy of hydration (*18*) (ΔH_H), entropy of hydration (*18*) (ΔS_H), free energy of hydration (*18*) (ΔG_H), magnetic susceptibility (*18*) (χ), polarizability (*18*) (α), softness (*18*) (σ), Jones-Dole viscosity parameter (*18*) (B), surface tension increments (*18*) ($d\sigma/dn$), and hydrogen bond disruption factor (*161*) (ΔG_{HB}) of each ion under study.

Ion	ΔH_H $\left(\frac{kJ}{mol}\right)$	ΔS_H $\left(\frac{J}{mol \cdot K}\right)$	ΔG_H $\left(\frac{kJ}{mol}\right)$	-χ $\left(\frac{mm^3}{kmol}\right)$	α $(Å^3)$	σ $(-)$	B $\left(\frac{L}{mol}\right)$	$d\sigma/dn$ $\left(\frac{mN \cdot L}{m \cdot mol}\right)$	ΔG_{HB} $(-)$
F^-	−510	−137.2	−469	13.0	0.88	−0.36	0.127	1.10	0.08
Cl^-	−367	−75.7	−344	28.0	3.42	0.21	−0.005	1.20	−0.61
Br^-	−336	−58.8	−318	39.0	4.85	0.47	−0.033	0.95	−0.80
I^-	−291	−35.9	−280	56.7	7.51	0.80	−0.073	0.35	−1.09
NO_2^-	−412	−93.7	−384	15.0	3.45	0.45	−0.024	---	−0.52
NO_3^-	−312	−83.4	−286	23.0	4.13	0.33	−0.045	0.45	−0.68
SO_4^{2-}	−1035	−200.4	−975	40.0	5.47	−0.08	0.206	1.15	−0.21
Li^+	−531	−141.8	−489	3.0	0.03	−1.32	0.146	0.65	0.28
Na^+	−416	−111.2	−383	2.3	0.26	−0.90	0.085	0.90	−0.03
K^+	−334	−74.3	−312	11.2	1.07	−0.88	−0.009	0.80	−0.52
Cs^+	−283	−58.6	−266	34.0	2.73	−0.84	−0.047	0.50	−0.69
NH_4^+	−329	−111.6	−301	65.0	1.86	−0.90	−0.008	0.40	−0.18
NMe_4^+	−209	−144.1	−166	65.0	9.08	0.51†	0.123	−0.40	−0.47
Mg^{2+}	−1949	−331.2	−1837	5.0	-0.28	−0.71	0.385	1.65	0.78
Ca^{2+}	−1602	−252.4	−1527	8.0	0.63	−0.96	0.298	1.50	0.34

†The value of 0.51 is corrected from a reported value of 0.11 in Marcus (*18*), following from values in the original source (*86*, *173*).

Table S15 continued: The bare radius (*18*) (r_b), hydrated radius (*162*) (r_H), Stokes radius (*162*) (r_S), the corresponding ion charge densities (CD_b, CD_H, and CD_S, respectively), and the radial charge density (*21*) (ρ) of each ion under study.

Ion	r_b (Å)	r_H (Å)	r_S (Å)	CD_b $\left(\frac{nC}{m^3}\right)$	CD_H $\left(\frac{nC}{m^3}\right)$	CD_S $\left(\frac{nC}{m^3}\right)$	ρ $\left(\frac{nC}{m \cdot mol}\right)$
F^-	1.33	1.66	3.52	−16.26	−8.36	−0.88	−0.864
Cl^-	1.81	1.21	3.32	−6.45	−21.60	−1.05	−0.625
Br^-	1.96	1.18	3.30	−5.08	−23.29	−1.06	−0.563
I^-	2.20	1.19	3.31	−3.59	−22.71	−1.06	−0.490
NO_2^-	1.92	---	---	−5.41	---	---	−0.648
NO_3^-	2.00	1.29	3.35	−4.78	−17.82	−1.02	−0.626
SO_4^{2-}	2.30	2.30	3.79	−6.29	−6.29	−1.41	−1.025
Li^+	0.69	2.38	3.82	116.47	2.84	0.69	3.161
Na^+	1.02	1.84	3.58	36.06	6.14	0.83	1.524
K^+	1.38	1.25	3.31	14.56	19.59	1.06	0.924
Cs^+	1.70	1.19	3.29	7.79	22.71	1.07	0.608
NH_4^+	1.48	1.25	3.31	11.80	19.59	1.06	0.960
NMe_4^+	2.80	2.05	3.67	1.74	4.44	0.77	0.540
Mg^{2+}	0.72	3.47	4.28	205.03	1.83	0.98	3.579
Ca^{2+}	1.00	3.10	4.12	76.53	2.57	1.09	2.094

REFERENCES

1. R. M. DuChanois, C. J. Porter, C. Violet, R. Verduzco, M. Elimelech, Membrane materials for selective ion separations at the water–energy nexus. *Adv. Mater.* **33**, 1–18 (2021).

2. T. Luo, S. Abdu, M. Wessling, Selectivity of ion exchange membranes: A review. *J. Membr. Sci.* **555**, 429–454 (2018).

3. R. M. DuChanois, N. J. Cooper, B. Lee, S. K. Patel, L. Mazurowski, T. E. Graedel, M. Elimelech, Prospects of metal recovery from wastewater and brine. *Nat. Water* **1**, 37–46 (2023).

4. IEA, "World Energy Outlook 2023" (IEA, 2023); https://iea.org/reports/world-energy-outlook-2023.

5. Z. H. Foo, J. B. Thomas, S. M. Heath, J. A. Garcia, J. H. Lienhard, Sustainable lithium recovery from hypersaline salt-lakes by selective electrodialysis: Transport and thermodynamics. *Environ. Sci. Technol.* **57**, 14747–14759 (2023).

6. Y. Zhang, L. Wang, W. Sun, Y. Hu, H. Tang, Membrane technologies for Li^{+}/Mg^{2+} separation from salt-lake brines and seawater: A comprehensive review. *J. Ind. Eng. Chem.* **81**, 7–23 (2020).

7. J. Zhang, Z. Cheng, X. Qin, X. Gao, M. Wang, X. Xiang, Recent advances in lithium extraction from salt lake brine using coupled and tandem technologies. *Desalination* **547**, 116225 (2023).

8. A. Razmjou, M. Asadnia, E. Hosseini, A. Habibnejad Korayem, V. Chen, Design principles of ion selective nanostructured membranes for the extraction of lithium ions. *Nat. Commun.* **10**, 5793 (2019).

9. C. Vautier, T. Kolbe, T. Babey, J. Marçais, B. W. Abbot, A. M. Laverman, Z. Thomas, L. Aquilina, G. Pinay, J.-R. de Dreuzy, What do we need to predict groundwater nitrate recovery trajectories? *Sci. Total Environ.* **788**, 147661 (2021).

10. M. Mohapatra, S. Anand, B. K. Mishra, D. E. Giles, P. Singh, Review of fluoride removal from drinking water. *J. Environ. Manage.* **91**, 67–77 (2009).

11. IAEA, "New developments and improvements in processing of 'problematic' radioactive waste" (IAEA-TECDOC-1579, International Atomic Energy Agency, 2007).

12. IAEA, "Remediation of Large Contaminated Areas Off-Site the Fukushima Dai-ichi NPP" (2011); https://iaea.org/sites/default/files/reportonfukushima2011.pdf.

13. D. S. Sholl, R. P. Lively, Seven chemical separations to change the world. *Nature* **532**, 435–437 (2016).

14. H. Fan, Y. Huang, N. Y. Yip, Advancing ion-exchange membranes to ion-selective membranes: Principles, status, and opportunities. *Front. Environ. Sci. Eng.* **17**, 25 (2023).

15. S. Jiang, H. Sun, H. Wang, B. P. Ladewig, Z. Yao, A comprehensive review on the synthesis and applications of ion exchange membranes. *Chemosphere* **282**, 130817 (2021).

16. K. P. Gregory, G. R. Elliott, H. Robertson, A. Kumar, E. J. Wanless, G. B. Webber, V. S. J. Craig, G. G. Andersson, A. J. Page, Understanding specific ion effects and the Hofmeister series. *Phys. Chem. Chem. Phys.* **24**, 12682–12718 (2022).

17. K. D. Collins, The behavior of ions in water is controlled by their water affinity. *Q. Rev. Biophys.* **52**, e11 (2019).

18. Y. Marcus, *Ions in Solution and Their Solvation* (John Wiley & Sons Inc., 2015).

19. S. Z. Moghaddam, E. Thormann, The Hofmeister series: Specific ion effects in aqueous polymer solutions. *J. Colloid Interface Sci.* **555**, 615–635 (2019).

20. F. Hofmeister, Zur Lehre von der Wirkung der Salze. *Arch. Exp. Path. Pharm.* **24**, 247–260 (1888).

21. K. P. Gregory, E. J. Wanless, G. B. Webber, V. S. J. Craig, A. J. Page, The electrostatic origins of specific ion effects: Quantifying the Hofmeister series for anions. *Chem. Sci.* **12**, 15007–15015 (2021).

22. K. D. Collins, Charge density-dependent strength of hydration and biological structure. *Biophys. J.* **72**, 65–76 (1997).

23. B. Tansel, Significance of thermodynamic and physical characteristics on permeation of ions during membrane separation: Hydrated radius, hydration free energy and viscous effects. *Sep. Purif. Technol.* **86**, 119–126 (2012).

24. K. D. Collins, Ion hydration: Implications for cellular function, polyelectrolytes, and protein crystallization. *Biophys. Chem.* **119**, 271–281 (2006).

25. R. G. Pearson, Hard and soft acids and bases. *J. Am. Chem. Soc.* **85**, 3533–3539 (1963).

26. H. J. Cassady, E. C. Cimino, M. Kumar, M. A. Hickner, Specific ion effects on the permselectivity of sulfonated poly(ether sulfone) cation exchange membranes. *J. Membr. Sci.* **508**, 146–152 (2016).

27. G. M. Geise, H. J. Cassady, D. R. Paul, B. E. Logan, M. A. Hickner, Specific ion effects on membrane potential and the permselectivity of ion exchange membranes. *Phys. Chem. Chem. Phys.* **16**, 21673–21681 (2014).

28. Y. Ji, H. Luo, G. M. Geise, Effects of fixed charge group physicochemistry on anion exchange membrane permselectivity and ion transport. *Phys. Chem. Chem. Phys.* **22**, 7283–7293 (2020).

29. Y. Li, T. Xu, Permselectivities of monovalent anions through pyridine-modified anion-exchange membranes. *Sep. Purif. Technol.* **61**, 430–435 (2008).

30. T. Sata, T. Yamaguchi, K. Matsusaki, Effect of hydrophobicity of ion exchange groups of anion exchange membranes on permselectivity between two anions. *J. Phys. Chem.* **99**, 12875–12882 (1995).

31. B. A. Soldano, G. E. Boyd, Self-diffusion of cations in hetero-ionic cation exchangers. *J. Am. Chem. Soc.* **75**, 6107–6110 (1953).

32. B. A. Soldano, G. E. Boyd, Self-diffusion of anions in strong-base anion exchangers. *J. Am. Chem. Soc.* **75**, 6099–6104 (1953).

33. G. E. Boyd, B. A. Soldano, Self-diffusion of water molecules and mobile anions in cation exchangers. *J. Am. Chem. Soc.* **75**, 6105–6107 (1953).

34. G. E. Boyd, B. A. Soldano, Self-diffusion of cations in and through sulfonated polystyrene cation-exchange polymers. *J. Am. Chem. Soc.* **75**, 6091–6099 (1953).

35. R. S. Kingsbury, M. A. Baird, J. Zhang, H. D. Patel, M. J. Baran, B. A. Helms, E. M. V. Hoek, Kinetic barrier networks reveal rate limitations in ion-selective membranes. *Matter* **7**, 2161–2183 (2024).

36. J. C. Díaz, J. Park, A. Shapiro, H. Patel, L. Santiago-Pagán, D. Kitto, J. Kamcev, Understanding monovalent cation diffusion in negatively charged membranes and the role of membrane water content. *Macromolecules* **57**, 2468–2481 (2024).

37. V. I. Volkov, A. V. Chernyak, D. V. Golubenko, V. A. Tverskoy, G. A. Lochin, E. S. Odjigaeva, A. B. Yaroslavtsev, Hydration and diffusion of H^+, Li^+, Na^+, Cs^+ ions in cation-exchange membranes based on polyethylene- and sulfonated-grafted polystyrene studied by NMR technique and ionic conductivity measurements. *Membranes* **10**, 272 (2020).

38. T. Badessa, V. Shaposhnik, The electrodialysis of electrolyte solutions of multi-charged cations. *J. Membr. Sci.* **498**, 86–93 (2016).

39. R. Epsztein, E. Shaulsky, M. Qin, M. Elimelech, Activation behavior for ion permeation in ion-exchange membranes: Role of ion dehydration in selective transport. *J. Membr. Sci.* **580**, 316–326 (2019).

40. C. L. Ritt, M. Liu, T. A. Pham, R. Epsztein, H. J. Kulik, M. Elimelech, Machine learning reveals key ion selectivity mechanisms in polymeric membranes with subnanometer pores. *Sci. Adv.* **8**, eabl5771 (2022).

41. S. Ahrland, "Thermodynamics of complex formation between hard and soft acceptors and donors" in *Structure and Bonding* (Springer Berlin Heidelberg, 1968), vol. 5, pp. 118–149.

42. A. P. Thompson, H. M. Aktulga, R. Berger, D. S. Bolintineanu, W. M. Brown, P. S. Crozier, P. J. in't Veld, A. Kohlmeyer, S. G. Moore, T. D. Nguyen, R. Shan, M. J. Stevens, J. Tranchida, C. Trott, S. J. Plimpton, LAMMPS—A flexible simulation tool for particle-based materials modeling at the atomic, meso, and continuum scales. *Comput. Phys. Commun.* **271**, 108171 (2022).

43. J. R. Gissinger, B. D. Jensen, K. E. Wise, Modeling chemical reactions in classical molecular dynamics simulations. *Polymer* **128**, 211–217 (2017).

44. J. R. Gissinger, B. D. Jensen, K. E. Wise, REACTER: A heuristic method for reactive molecular dynamics. *Macromolecules* **53**, 9953–9961 (2020).

45. J. L. F. Abascal, C. Vega, A general purpose model for the condensed phases of water: TIP4P/2005. *J. Chem. Phys.* **123**, 234505 (2005).

46. I. S. Joung, T. E. Cheatham, Determination of Alkali and Halide monovalent ion parameters for use in explicitly solvated biomolecular simulations. *J. Phys. Chem. B* **112**, 9020–9041 (2008).

47. M. F. Döpke, O. A. Moultos, R. Hartkamp, On the transferability of ion parameters to the TIP4P/2005 water model using molecular dynamics simulations. *J. Chem. Phys.* **152**, 024501 (2020).

48. W. L. Jorgensen, D. S. Maxwell, J. Tirado-Rives, Development and testing of the OPLS all-atom force field on conformational energetics and properties of organic liquids. *J. Am. Chem. Soc.* **118**, 11225–11236 (1996).

49. L. S. Dodda, I. Cabeza de Vaca, J. Tirado-Rives, W. L. Jorgensen, LigParGen web server: An automatic OPLS-AA parameter generator for organic ligands. *Nucleic Acids Res.* **45**, W331–W336 (2017).

50. B. J. Zwolinski, H. Eyring, C. E. Reese, Diffusion and membrane permeability. *J. Phys. Chem.* **53**, 1426–1453 (1949).

51. S. Glasstone, K. J. Laidler, H. Eyring, *The Theory of Rate Processes* (McGraw-Hill, 1941).

52. J. C. Díaz, D. Kitto, J. Kamcev, Accurately measuring the ionic conductivity of membranes via the direct contact method. *J. Membr. Sci.* **669**, 121304 (2023).

53. J. O. Bockris, A. K. N. Reddy, *Modern Electrochemistry 1: Ionics* (Kluwer Academic Publishers, ed. 1, 1998).

54. I. Shefer, K. Lopez, A. P. Straub, R. Epsztein, Applying transition-state theory to explore transport and selectivity in salt-rejecting membranes: A critical review. *Environ. Sci. Technol.* **56**, 7467–7483 (2022).

55. I. Shefer, O. Peer-Haim, R. Epsztein, Limited ion-ion selectivity of salt-rejecting membranes due to enthalpy-entropy compensation. *Desalination* **541**, 116041 (2022).

56. S. Yadav, A. Chandra, Solvation shell of the nitrite ion in water: An ab initio molecular dynamics study. *J. Phys. Chem. B* **124**, 7194–7204 (2020).

57. E. B. Starikov, B. Nordén, Enthalpy−entropy compensation: A phantom or something useful? *J. Phys. Chem. B* **111**, 14431–14435 (2007).

58. R. S. Prabhakar, R. Raharjo, L. G. Toy, H. Lin, B. D. Freeman, Self-consistent model of concentration and temperature dependence of permeability in rubbery polymers. *Ind. Eng. Chem. Res.* **44**, 1547–1556 (2005).

59. K. M. Diederichsen, H. G. Buss, B. D. McCloskey, The compensation effect in the Vogel-Tammann-Fulcher (VTF) equation for polymer-based electrolytes. *Macromolecules* **50**, 3831–3840 (2017).

60. Y. Marcus, G. Hefter, Ion pairing. *Chem. Rev.* **106**, 4585–4621 (2006).

61. R. Sujanani, O. Nordness, A. Miranda, L. E. Katz, J. F. Brennecke, B. D. Freeman, Accounting for ion pairing effects on sulfate salt sorption in cation exchange membranes. *J. Phys. Chem. B* **127**, 1842–1855 (2023).

62. A. A. Chialvo, J. M. Simonson, Solvation behavior of short-chain polystyrene sulfonate in aqueous electrolyte solutions: A molecular dynamics study. *J. Phys. Chem. B* **109**, 23031–23042 (2005).

63. A. A. Chialvo, J. M. Simonson, Ion pairing and counterion condensation in aqueous electrolyte and polyelectrolyte solutions: Insights from molecular simulation. *J. Mol. Liq.* **134**, 15–22 (2007).

64. T. Megyes, I. Bakó, S. Bálint, T. Grósz, T. Radnai, Ion pairing in aqueous calcium chloride solution: Molecular dynamics simulation and diffraction studies. *J. Mol. Liq.* **129**, 63–74 (2006).

65. R. A. Robinson, R. H. Stokes, *Electrolyte Solutions* (Dover Publications Inc., ed. 2, revised, 1965).

66. C. J. Fennell, A. Bizjak, V. Vlachy, K. A. Dill, Ion pairing in molecular simulations of aqueous alkali halide solutions. *J. Phys. Chem. B* **113**, 6782–6791 (2009).

67. N. Marioni, A. Rajesh, Z. Zhang, B. D. Freeman, V. Ganesan, What is the influence of ion aggregation and counterion condensation on salt transport in ion exchange membranes? *J. Membr. Sci.* **701**, 122713 (2024).

68. Z. R. Kann, J. L. Skinner, A scaled-ionic-charge simulation model that reproduces enhanced and suppressed water diffusion in aqueous salt solutions. *J. Chem. Phys.* **141**, 104507 (2014).

69. Z. O. Memar, M. Moosavi, Assessing OPLS-based force fields for investigating the characteristics of imidazolium-based dicationic ionic liquids: A comparative study with AIMD simulations and experimental findings. *J. Chem. Phys.* **159**, 244504 (2023).

70. B. Doherty, X. Zhong, S. Gathiaka, B. Li, O. Acevedo, Revisiting OPLS force field parameters for ionic liquid simulations. *J. Chem. Theory Comput.* **13**, 6131–6145 (2017).

71. K. M. Callahan, N. N. Casillas-Ituarte, M. Roeselová, H. C. Allen, D. J. Tobias, Solvation of magnesium dication: Molecular dynamics simulation and vibrational spectroscopic study of magnesium chloride in aqueous solutions. *J. Phys. Chem. A* **114**, 5141–5148 (2010).

72. R. Buchner, T. Chen, G. Hefter, Complexity in "simple" electrolyte solutions: Ion pairing in $MgSO_4$(aq). *J. Phys. Chem. B* **108**, 2365–2375 (2004).

73. F. P. Daly, C. W. Brown, D. R. Kester, Sodium and magnesium sulfate ion pairing. Evidence from Raman spectroscopy. *J. Phys. Chem.* **76**, 3664–3668 (1972).

74. G. Hefter, When spectroscopy fails: The measurement of ion pairing. *Pure Appl. Chem.* **78**, 1571–1586 (2006).

75. C. D. Alcorn, J. S. Cox, L. M. S. G. A. Applegarth, P. R. Tremaine, Investigation of uranyl sulfate complexation under hydrothermal conditions by quantitative raman spectroscopy and density functional theory. *J. Phys. Chem. B* **123**, 7385–7409 (2019).

76. W. Rudolph, G. Irmer, Raman and infrared spectroscopic investigation of contact ion pair formation in aqueous cadmium sulfate solutions. *J. Solution Chem.* **23**, 663–684 (1994).

77. V. Pavluchkov, I. Shefer, O. Peer-Haim, J. Blotevogel, R. Epsztein, Indications of ion dehydration in diffusion-only and pressure-driven nanofiltration. *J. Membr. Sci.* **648**, 120358 (2022).

78. C. Lu, C. Hu, C. L. Ritt, X. Hua, J. Sun, H. Xia, Y. Liu, D.-W. Li, B. Ma, M. Elimelech, J. Qu, In situ characterization of dehydration during ion transport in polymeric nanochannels. *J. Am. Chem. Soc.* **143**, 14242–14252 (2021).

79. T. Tran, C. Lin, S. Chaurasia, H. Lin, Elucidating the relationship between states of water and ion transport properties in hydrated polymers. *J. Membr. Sci.* **574**, 299–308 (2019).

80. B. Vondrasek, C. Wen, S. Cheng, J. S. Riffle, J. J. Lesko, On the nature of freezing/melting water in ionic polysulfones. *Macromolecules* **54**, 6477–6488 (2021).

81. C. Espinoza, J. Díaz, D. Kitto, H. Kim, J. Kamcev, Bound water enhances the ion selectivity of highly charged polymer membranes. *ACS Appl. Mater. Interfaces* **16**, 45433–45446 (2024).

82. A. Higuchi, T. Ijima, DSC Investigation of the states of water in poly(vinyl alcohol) membranes. *Polymer* **26**, 1207–1211 (1985).

83. R. M. Hodge, G. H. Edward, G. P. Simon, Water absorption and states of water in semicrystalline poly(vinyl alcohol) films. *Polymer* **37**, 1371–1376 (1996).

84. N. F. A. Van Der Vegt, K. Haldrup, S. Roke, J. Zheng, M. Lund, H. J. Bakker, Water-mediated ion pairing: Occurrence and relevance. *Chem. Rev.* **116**, 7626–7641 (2016).

85. S. Ahrland, Scales of softness for acceptors and donors. *Chem. Phys. Lett.* **2**, 303–306 (1968).

86. Y. Marcus, *Ion Properties* (CRC Press, ed. 1, 1997).

87. S. Ahrland, J. Chatt, N. R. Davies, The relative affinities of ligand atoms for acceptor molecules and ions. *Q. Rev. Chem. Soc.* **12**, 265 (1958).

88. R. G. Pearson, Hard and soft acids and bases, HSAB, part 1: Fundamental principles. *J. Chem. Educ.* **45**, 581 (1968).

89. R. G. Pearson, Acids and bases: Hard acids prefer to associate with hard bases, and soft acids prefer to associate with soft bases. *Science* **151**, 172–177 (1966).

90. R. G. Pearson, The principle of maximum hardness. *Acc. Chem. Res.* **26**, 250–255 (1993).

91. Y. Marcus, The softness parameters of ions. prediction of the occurrence of miscibility gaps in molten salt mixtures. *Isr. J. Chem.* **10**, 659–683 (1972).

92. Y. Marcus, On enthalpies of hydration, ionization potentials, and the softness of ions. *Thermochim. Acta* **104**, 389–394 (1986).

93. T. J. Boerner, S. Deems, T. R. Furlani, S. L. Knuth, J. Towns, "ACCESS: Advancing Innovation: NSF's Advanced Cyberinfrastructure Coordination Ecosystem: Services & Support," in *Practice and Experience in Advanced Research Computing* (ACM, 2023), pp. 173–176.

94. J. Kamcev, D. R. Paul, B. D. Freeman, Effect of fixed charge group concentration on equilibrium ion sorption in ion exchange membranes. *J. Mater. Chem. A* **5**, 4638–4650 (2017).

95. P. H. C. Eilers, H. F. M. Boelens, "Baseline Correction with Asymmetric Least Squares Smoothing" (Leiden Univ. Medical Centre, 2005).

96. E. Grau-Luque, F. Atlan, I. Becerril-Romero, A. Perez-Rodriguez, M. Guc, V. Izquierdo-Roca, spectrapepper: A Python toolbox for advanced analysis of spectroscopic data for materials and devices. *J. Open Source Softw.* **6**, 3781 (2021).

97. D. Brownstone, R. Valletta, The bootstrap and multiple imputations: Harnessing increased computing power for improved statistical tests. *J. Econ. Perspect.* **15**, 129–141 (2001).

98. S. J. Choquette, E. S. Etz, W. S. Hurst, D. H. Blackburn, S. D. Leigh, Relative intensity correction of Raman spectrometers: NIST SRMs 2241 through 2243 for 785 nm, 532 nm, and 488 nm/514.5 nm excitation. *Appl. Spectrosc.* **61**, 117–129 (2007).

99. N. J. Silva, D. Tunega, C. Korzeniewski, H. Lischka, A. J. A. Aquino, Microhydration of polymer electrolyte membranes: A comparison of hydrogen-bonding networks and spectral properties of nafion and Bis[(perfluoroalkyl)sulfonyl] imide. *J. Phys. Chem. B* **123**, 9899–9911 (2019).

100. W. Kujawski, Q. T. Nguyen, J. Neel, Infrared investigations of sulfonated ionomer membranes. I. Water–alcohol compositions and counterions effects. *J. Appl. Polym. Sci.* **44**, 951–958 (1992).

101. C. K. Byun, I. Sharif, D. D. DesMarteau, S. E. Creager, C. Korzeniewski, Infrared spectroscopy of bis[(perfluoroalkyl)sulfonyl] imide ionomer membrane materials. *J. Phys. Chem. B* **113**, 6299–6304 (2009).

102. G. Socrates, *Infrared and Raman Characteristic Group Frequencies: Tables and Charts* (John Wiley & Sons Inc., ed. 3, 2001).

103. NIST, Tetramethylammonium chloride, https://webbook.nist.gov/cgi/inchi/InChI%3D1S/C4H12N.ClH/c1-5(2%2C3)4%3B/h1-4H3%3B1H/q%2B1%3B/p-1.

104. M. J. Frisch, G. W. Trucks, H. B. Schlegel, G. E. Scuseria, M. A. Robb, J. R. Cheeseman, G. Scalmani, V. Barone, G. A. Petersson, H. Nakatsuji, X. Li, M. Caricato, A. V. Marenich, J. Bloino, B. G. Janesko, R. Gomperts, B. Mennucci, H. P. Hratchian, J. V. Ortiz, A. F. Izmaylov, J. L. Sonnenberg, D. Williams-Young, F. Ding, F. Lipparini, F. Egidi, J. Goings, B. Peng, A. Petrone, T. Henderson, D. Ranasinghe, V. G. Zakrzewski, J. Gao, N. Rega, G. Zheng, W. Liang, M. Hada, M. Ehara, K. Toyota, R. Fukuda, J. Hasegawa, M. Ishida, T. Nakajima, Y. Honda, O. Kitao, H. Nakai, T. Vreven, K. Throssell, J. A. Montgomery Jr., J. E. Peralta, F. Ogliaro, M. J. Bearpark, J. J. Heyd, E. N. Brothers, K. N. Kudin, V. N. Staroverov, T. A. Keith, R. Kobayashi, J. Normand, K. Raghavachari, A. P. Rendell, J. C. Burant, S. S. Iyengar, J. Tomasi, M. Cossi, J. M. Millam, M. Klene, C. Adamo, R. Cammi, J. W. Ochterski, R. L. Martin, K. Morokuma, O. Farkas, J. B. Foresman, D. J. Fox, Gaussian 16 Revision C.01 (2016); https://gaussian.com/.

105. A. D. Becke, Density-functional thermochemistry. III. The role of exact exchange. *J. Chem. Phys.* **98**, 5648–5652 (1993).

106. E. Bodo, M. Bonomo, A. Mariani, Assessing the structure of protic ionic liquids based on triethylammonium and organic acid anions. *J. Phys. Chem. B* **125**, 2781–2792 (2021).

107. J. Malenfant, L. Kuster, Y. Gagné, K. Signo, M. Denis, S. Canesi, M. Frenette, Towards routine organic structure determination using Raman microscopy. *Chem. Sci.* **15**, 701–709 (2024).

108. F. Weigend, R. Ahlrichs, Balanced basis sets of split valence, triple zeta valence and quadruple zeta valence quality for H to Rn: Design and assessment of accuracy. *Phys. Chem. Chem. Phys.* **7**, 3297–3305 (2005).

109. E. Cancès, B. Mennucci, J. Tomasi, A new integral equation formalism for the polarizable continuum model: Theoretical background and applications to isotropic and anisotropic dielectrics. *J. Chem. Phys.* **107**, 3032–3041 (1997).

110. A. Silverman, B. R. Goldsmith, J. Kamcev, Specific ion effects on ion transport in charged polymer membranes: Simulated Raman spectra from density functional theory, Zenodo (2025); https://doi.org/10.5281/zenodo.17273515.

111. R. B. McCleskey, Electrical conductivity of electrolytes found in natural waters from (5 to 90) °C. *J. Chem. Eng. Data* **56**, 317–327 (2011).

112. A. C. F. Ribeiro, M. C. F. Barros, A. S. N. Teles, A. J. M. Valente, V. M. M. Lobo, A. J. F. N. Sobral, M. A. Esteso, Diffusion coefficients and electrical conductivities for calcium chloride aqueous solutions at 298.15 K and 310.15 K. *Electrochim. Acta* **54**, 192–196 (2008).

113. T. L. Broadwater, D. F. Evans, The conductance of divalent ions in H_2O at 10 and 25°C and in D_2O. *J. Solution Chem.* **3**, 757–769 (1974).

114. H. S. Dunsmore, S. K. Jalota, R. Paterson, Irreversible thermodynamic parameters for isothermal vectorial transport processes in aqueous caesium chloride solutions. *J. Chem. Soc. A* , 1061–1065 (1969).

115. C. G. Swain, D. F. Evans, Conductance of ions in light and heavy water at 25°. *J. Am. Chem. Soc.* **88**, 383–390 (1966).

116. D. G. Miller, Application of irreversible thermodynamics to electrolyte solutions. I. Determination of ionic transport coefficients l_{ij} for isothermal vector transport processes in binary electrolyte systems. *J. Phys. Chem.* **70**, 2639–2659 (1966).

117. A. N. Campbell, E. Bock, The limiting equivalent conductances of ammonium chloride, ammonium bromide, and ammonium nitrate at 35.00°C. *Can. J. Chem.* **36**, 330–338 (1958).

118. N. G. Foster, E. S. Amis, The equivalent conductance of electrolytes in mixed solvents. I. Potassium chloride in the water-methanol system. *Z. Phys. Chem.* **3**, 365–381 (1955).

119. H. M. Daggett Jr., E. J. Bair, C. A. Kraus, Properties of electrolytic solutions. XLVII. Conductance of some quaternary ammonium and other salts in water at low concentration. *J. Am. Chem. Soc.* **73**, 799–803 (1951).

120. M. J. McDowell, C. A. Kraus, Properties of electrolytic solutions. XLIX. Conductance of some salts in water at 25°. *J. Am. Chem. Soc.* **73**, 2170–2173 (1951).

121. I. L. Jenkins, C. B. Monk, The conductances of sodium, potassium and lanthanum sulfates at 25°. *J. Am. Chem. Soc.* **72**, 2695–2698 (1950).

122. B. B. Owen, H. Zeldes, The conductance of potassium chloride, potassium bromide and potassium iodide in aqueous solutions from 5 to 55°. *J. Chem. Phys.* **18**, 1083–1085 (1950).

123. G. C. Benson, A. R. Gordon, The conductance of aqueous solutions of calcium chloride at temperatures from 15° to 45°C. *J. Chem. Phys.* **13**, 470–472 (1945).

124. G. C. Benson, A. R. Gordon, A reinvestigation of the conductance of aqueous solutions of potassium chloride, sodium chloride, and potassium bromide at temperatures from 15° to 45°C. *J. Chem. Phys.* **13**, 473–474 (1945).

125. H. E. Gunning, A. R. Gordon, The conductance of aqueous solutions of potassium bromide at temperatures from 15° to 45°C, and the limiting mobility of bromide ion. *J. Chem. Phys.* **11**, 18–20 (1943).

126. H. E. Gunning, A. R. Gordon, The conductance and ionic mobilities for aqueous solutions of potassium and sodium chloride at temperatures from 15° to 45°C. *J. Chem. Phys.* **10**, 126–131 (1942).

127. N. C. C. Li, W. Brüll, Conductivity studies. IV. The limiting ionic mobilities of several univalent ions at Temperatures between 15 and 45°. *J. Am. Chem. Soc.* **64**, 1635–1637 (1942).

128. N. C. C. Li, H. Fang, Conductivity studies. III. The limiting equivalent conductances of potassium chloride in water at temperatures between 15 and 40°. *J. Am. Chem. Soc.* **64**, 1544–1547 (1942).

129. J. Lange, Zur Leitfähigkeit starker Elektrolyte. *Z. Phys. Chem.* **188A**, 284–315 (1941).

130. N. C. Sen-Gupta, S. K. Mitra, Variation of the absolute rates of migration and transport number of electrolytic ions with dilution. *J. Indian Chem. Soc.* **14**, 645–652 (1937).

131. G. Jantsch, H. Grubitsch, E. Lischka, Über die elektrolytischen Leitfähigkeiten wässeriger Lösungen der Halogenide der seltenen Erden. *Z. Elektrochem. Angew. Phys. Chem.* **43**, 293–296 (1937).

132. T. Shedlovsky, A. S. Brown, The electrolytic conductivity of alkaline earth chlorides in water at 25°. *J. Am. Chem. Soc.* **56**, 1066–1071 (1934).

133. C. J. B. Clews, The electrical conductivity of strong electrolytes and its variation with temperature. *Proc. Phys. Soc.* **46**, 764–771 (1934).

134. G. Jones, C. F. Bickford, The conductance of aqueous solutions as a function of the concentration. I. Potassium bromide and lanthanum chloride. *J. Am. Chem. Soc.* **56**, 602–611 (1934).

135. G. Scatchard, S. S. Prentiss, The freezing points of aqueous solutions. IV. Potassium, sodium and lithium chlorides and bromides. *J. Am. Chem. Soc.* **55**, 4355–4362 (1933).

136. P. Ekwall, Die Leitfähigkeit alkylierter Ammoniumpikrate in wässeriger Lösung bei 0°, 25° und 90°. III. Über die Hydrolyse der untersuchten Salze. *Z. Phys. Chem.* **165A**, 331–337 (1933).

137. A. Klemenc, E. Hayek, Zur Kenntnis der Dissoziationskonstante der salpetrigen Säure. *Montash. Chem.* **53**, 407–412 (1929).

138. R. T. Lattey, XXIII. The influence of the solvent on the mobility of electrolytic ions. *Lond. Edinb. Dubl. Philos. Mag. J. Sci.* **6**, 258–270 (1928).

139. A. Ferguson, I. Vogel, XIX. The calculation of the equivalent conductivity of strong electrolytes.—Part I. Aqueous solutions. (ii.) Application to data at 0°, 18°, and 25° C. *Lond. Edinb. Dubl. Philos. Mag. J. Sci.* **4**, 233–242 (1927).

140. A. Ferguson, I. Vogel, CV. The calculation of the equivalent conductivity of strong electrolytes at infinite dilution. Part I.—Aqueous solutions. *Lond. Edinb. Dubl. Philos. Mag. J. Sci.* **50**, 971–985 (1925).

141. A. A. Noyes, K. G. Falk, The properties of salt solutions in relation to the ionic theory. III. Electrical conductance. *J. Am. Chem. Soc.* **34**, 454–485 (1912).

142. H. C. Jones, "*The Electrical Conductivity, Dissociation, and Temperature Coefficients of Conductivity from Zero to Sixty-Five Degrees of Aqueous Solutions of a Number of Salts and Organic Acids*" (Carnegie Institution of Washington, 1912), vol. 170.

143. A. A. Noyes, A. C. Melcher, H. C. Cooper, G. W. Eastman, The conductivity and ionization of salts, acids, and bases in aqueous solutions at high temperatures. *Z. Phys. Chem.* **70U**, 335–377 (1910).

144. J. Johnston, The change of the equivalent conductance of ions with the temperature. *J. Am. Chem. Soc.* **31**, 1010–1020 (1909).

145. A. A. Noyes, A. C. Melcher, H. C. Cooper, G. W. Eastman, Y. Kato, The conductivity and ionization of salts, acids, and bases in aqueous solutions at high temperatures. *J. Am. Chem. Soc.* **30**, 335–353 (1908).

146. R. T. Lattey, LXXV. On the dilution law for strong electrolytes. *Lond. Edinb. Dubl. Philos. Mag. J. Sci.* **4**, 831–836 (1927).

147. H. S. Harned, B. B. Owen, “Chapter 4” in *The Physical Chemistry of Electrolytic Solutions* (Reinhold Pub. Corp., ed. 3, 1958), pp. 233–234.

148. R. W. Allgood, D. J. Le Roy, A. R. Gordon, The variation of the transference numbers of potassium chloride in aqueous solution with temperature. *J. Chem. Phys.* **8**, 418–422 (2004).

149. J. Crank, G. S. Park, *Diffusion in Polymers* (Academic Press Inc., ed. 1, 1968).

150. N. Lakshminarayanaiah, *Transport Phenomena in Membranes* (Academic Press Inc., ed. 1, 1969).

151. F. Helfferich, *Ion Exchange* (Dover Science Books, 1995).

152. H. Strathmann, *Ion-Exchange Membrane Separation Processes* (Elsevier, 2004).

153. A. C. F. Ribeiro, A. J. M. Valente, A. J. F. N. Sobral, V. M. M. Lobo, H. D. Burrows, M. A. Esteso, Diffusion coefficients of aluminium chloride in aqueous solutions at 298.15, 303.15 and 315.15 K. *Electrochim. Acta* **52**, 6450–6455 (2007).

154. N. Marioni, Z. Zhang, E. S. Zofchak, H. S. Sachar, S. Kadulkar, B. D. Freeman, V. Ganesan, Impact of ion–ion correlated motion on salt transport in solvated ion exchange membranes. *ACS Macro Lett.* **11**, 1258–1264 (2022).

155. L. Masaro, X. X. Zhu, Physical models of diffusion for polymer solutions, gels, and solids. *Prog. Polym. Sci.* **24**, 731–775 (1999).

156. J. S. Mackie, P. Meares, The diffusion of electrolytes in a cation-exchange resin membrane. *Proc. R. Soc. London Ser. A* **232**, 498–509 (1955).

157. A. Kusoglu, A. Z. Weber, New insights into perfluorinated sulfonic-acid ionomers. *Chem. Rev.* **117**, 987–1104 (2017).

158. N. Brauner, M. Shacham, Statistical analysis of linear and nonlinear correlation of the Arrhenius equation constants. *Chem. Eng. Process. Process Intensif.* **36**, 243–249 (1997).

159. D. York, Least-squares fitting of a straight line. *Can. J. Phys.* **44**, 1079–1086 (1966).

160. C. F. J. Wu, Jackknife, bootstrap and other resampling methods in regression analysis. *Ann. Statist.* **14**, 1261–1295 (1986).

161. Y. Marcus, Viscosity*B*-coefficients, structural entropies and heat capacities, and the effects of ions on the structure of water. *J. Solution Chem.* **23**, 831–848 (1994).

162. E. R. Nightingale Jr., Phenomenological theory of ion solvation. Effective Radii of hydrated ions. *J. Phys. Chem.* **63**, 1381–1387 (1959).

163. B. Efron, R. Tibshirani, *An Introduction to the Bootstrap*, Monographs on Statistics and Applied Probability (Chapman & Hall, 1998).

164. E. R. Malinowski, *Factor Analysis in Chemistry* (Wiley, ed. 3, 2002).

165. L. T. Biegler, J. J. Damiano, G. E. Blau, Nonlinear parameter estimation: A case study comparison. *AIChE J.* **32**, 29–45 (1986).

166. S. P. Kadaoluwa Pathirannahalage, N. Meftahi, A. Elbourne, A. C. G. Weiss, C. F. McConville, A. Padua, D. A. Winkler, M. Costa Gomes, T. L. Greaves, T. C. Le, Q. A. Besford, A. J. Christofferson, Systematic comparison of the structural and dynamic properties of commonly used water models for molecular dynamics simulations. *J. Chem. Inf. Model.* **61**, 4521–4536 (2021).

167. W. Shinoda, M. Shiga, M. Mikami, Rapid estimation of elastic constants by molecular dynamics simulation under constant stress. *Phys. Rev. B* **69**, 134103 (2004).

168. R. Vickers, A. Silverman, B. R. Goldsmith, J. Kamcev, Specific Ion Effects on Ion Transport in Charged Polymer Membranes: Molecular Dynamics Parameter, Molecule, and Input Files, Zenodo (2025); https://doi.org/10.5281/zenodo.17274528.

169. R. Vickers, T. M. Weigand, C. T. Miller, O. Coronell, Molecular methods for assessing the morphology, topology, and performance of polyamide membranes. *J. Membr. Sci.* **644**, 120110 (2022).

170. G. M. Torrie, J. P. Valleau, Nonphysical sampling distributions in Monte Carlo free-energy estimation: Umbrella sampling. *J. Comput. Phys.* **23**, 187–199 (1977).

171. S. Kumar, J. M. Rosenberg, D. Bouzida, R. H. Swendsen, P. A. Kollman, The weighted histogram analysis method for free-energy calculations on biomolecules. I. The method. *J. Comput. Chem.* **13**, 1011–1021 (1992).

172. P. Sipos, L. Bolden, G. Hefter, P. M. May, Raman spectroscopic study of ion pairing of alkali metal ions with carbonate and sulfate in aqueous solutions. *Aust. J. Chem.* **53**, 887 (2000).

173. S. Glikberg, Y. Marcus, Relation of the Gibbs free energy of transfer of ions from water to polar solvents to the properties of the solvents and the ions. *J. Solution Chem.* **12**, 255–270 (1983).
